# Knowledge support for optimising antibiotic prescribing for common infections in general practices: evaluation of the effectiveness of periodic feedback, decision support during consultations and peer comparisons in a cluster randomised trial (BRIT2) – study protocol

**DOI:** 10.1136/bmjopen-2023-076296

**Published:** 2023-08-22

**Authors:** Tjeerd van Staa, Anita Sharma, Victoria Palin, Ali Fahmi, Harriet Cant, Xiaomin Zhong, Francine Jury, Natalie Gold, William Welfare, Darren Ashcroft, Jung Yin Tsang, Rachel Ann Elliott, Christopher Sutton, Chris Armitage, Philip Couch, Georgina Moulton, Edward Tempest, Iain Edward Buchan

**Affiliations:** 1 Centre for Health Informatics, The University of Manchester, Manchester, UK; 2 Chadderton South Health Centre, Oldham, UK; 3 Faculty of Biology, Medicine and Health, The University of Manchester, Manchester, UK; 4 Faculty of Philosophy, University of Oxford, Oxford, UK; 5 Regions Directorate, UK Health Security Agency, London, UK; 6 NIHR School for Primary Care Research, Centre for Primary Care and Health Services Research, The University of Manchester, Manchester, UK; 7 Centre for Primary Care and Health Services Research, University of Manchester, Manchester, UK; 8 Manchester Centre for Health Economics, The University of Manchester, Manchester, UK; 9 Division of Population Health, Health Services Research & Primary Care, University of Manchester, Manchester, UK; 10 Manchester Centre for Health Psychology, University of Manchester, Manchester, UK; 11 Public Health and Policy, University of Liverpool, Liverpool, UK

**Keywords:** Primary Care, Electronic Health Records, INFECTIOUS DISEASES, Randomized Controlled Trial

## Abstract

**Introduction:**

This project applies a Learning Healthcare System (LHS) approach to antibiotic prescribing for common infections in primary care. The approach involves iterations of data analysis, feedback to clinicians and implementation of quality improvement activities by the clinicians. The main research question is, can a knowledge support system (KSS) intervention within an LHS implementation improve antibiotic prescribing without increasing the risk of complications?

**Methods and analysis:**

A pragmatic cluster randomised controlled trial will be conducted, with randomisation of at least 112 general practices in North-West England. General practices participating in the trial will be randomised to the following interventions: periodic practice-level and individual prescriber feedback using dashboards; or the same dashboards plus a KSS. Data from large databases of healthcare records are used to characterise heterogeneity in antibiotic uses, and to calculate risk scores for clinical outcomes and for the effectiveness of different treatment strategies. The results provide the baseline content for the dashboards and KSS. The KSS comprises a display within the electronic health record used during the consultation; the prescriber (general practitioner or allied health professional) will answer standard questions about the patient’s presentation and will then be presented with information (eg, patient’s risk of complications from the infection) to guide decision making. The KSS can generate information sheets for patients, conveyed by the clinicians during consultations. The primary outcome is the practice-level rate of antibiotic prescribing (per 1000 patients) with secondary safety outcomes. The data from practices participating in the trial and the dashboard infrastructure will be held within regional shared care record systems of the National Health Service in the UK.

**Ethics and dissemination:**

Approved by National Health Service Ethics Committee IRAS 290050. The research results will be published in peer-reviewed journals and also disseminated to participating clinical staff and policy and guideline developers.

**Trial registration number:**

ISRCTN16230629.

**STRENGTHS AND LIMITATIONS OF THIS STUDY**

- This protocol describes a pragmatic cluster randomised controlled trial with randomisation of general practices to periodic practice-level and individual prescriber feedback using dashboards only compared with dashboards plus a knowledge support system (KSS) that can be activated during consultations.
- These interventions will be applied to antibiotic prescribing for common infections in primary care, an important area for clinical improvement given rising antimicrobial resistance.
- The design of the KSS was informed by two mixed-methods codesign workshops in which clinicians identified: key information to extract from care records (such as antibiotic prescribing history); recommended actions; personalised treatments; risk indicators and content for patient information sheets.
- The primary research question is what is the effect on antibiotic prescribing of the KSS intervention within a learning health system implementation?
- A pilot phase will be initially conducted, with the recruitment target of 20 practices across two regions to examine feasibility and acceptability.

## Introduction

Translating research findings into routine clinical practice is a major challenge. The Learning Healthcare System (LHS) approach has been proposed to better integrate research into clinical practice.^1^ It involves iterations of data analysis (data to knowledge), feedback to clinicians (knowledge to performance) and implementation of quality improvement activities by clinicians (performance to data).^2 3^ In the LHS, feedback can either be provided periodically through for example online dashboards or during consultations with patients using a knowledge support System (KSS). We use the term KSS as distinct from a Clinical Decision Support System because the information provided is about the condition, common complications and treatment options in general rather than specific decisions. Activation of KSS linked to electronic health records (EHRs) has been proposed for augmenting clinicians’ knowledge during consultations with patients.^4^ An icon on the clinicians’ computer screen provides access to information on ‘patients like mine’ (eg, the risk of developing clinical complications in similar patients), recommendations (eg, best not prescribe amoxicillin given this patient’s frequent prior use) and a patient information sheet tailored to their condition and treatment. The information on similar patients could be drawn from several sources such as historic data on clinical outcomes from comparable patient groups.^4^ Thus, the KSS provides contextual information where a Clinical Decision Support System would point to a desired decision.^5^

In this project, the LHS approach will be applied to antibiotic prescribing for common infections in primary care. Primary care accounts for 72% of antibiotic prescribing in England.^6^ Overuse of antibiotics is a major public health concern as it increases antimicrobial resistance. The National Health Service (NHS) 2019 Long Term Plan pledged action to optimise antibiotic uses, reducing inappropriate prescribing. The LHS approach will also include personalised patient information sheets that the clinician can provide to patients during consultation after KSS activation. A recent Cochrane review found that people exposed to decision aids feel more knowledgeable, better informed and clearer about their values. It also found improved knowledge and accurate risk perceptions when decision aids are used within the consultation.^7^

### Research questions

The research question is whether a KSS intervention within an LHS implementation can improve antibiotic prescribing without increasing the risk of complications? Practices that have implemented the KSS will be compared with practices that have not (ie, randomised cluster trial). The LHS approach will involve detailed data analysis followed by feedback to clinicians.

## Methods and analysis

### Prior development of study interventions

#### Periodic feedback

A previous project piloted and implemented the IT infrastructure for the LHS (ie, data analytics, feedback to clinicians) on antibiotic prescribing care in UK primary care. This project (BRIT: Building Rapid Interventions to reduce AMR and over-prescribing of antibiotics) was part of the £20 million Department of Health and Social Care funded Connected Health Cities programme (https://www.connectedhealthcities.org/). This initial BRIT project developed practice-level feedback on antibiotic together with feedback on individual prescribers.^8^ These dashboards comprise a variety of antibiotic prescribing measures that were developed with clinical stakeholders.^9^ They currently provide information at practice level on the drivers of antibiotic prescribing (overall and by indication), analyses of prescribing of inappropriate types of antibiotics (deviating from guideline) and extent of risk-based prescribing. Importantly, the dashboards will allow clinicians to review data at different levels, including for example prescribing rates of a specific antibiotic to the individual patients prescribed that antibiotic, and comparison to other practices in the region.^9^

#### KSS during consultation

Our KSS intervention was developed based on the results of two mixed-methods codesign workshops to gauge the acceptability of a prototype.^10^ Clinicians identified the following key requirements: ease and efficiency of use, integration of systems, patient-centeredness, personalisation and training. The KSS needs to include extraction of pertinent information from the care record (such as antibiotic prescribing history), recommended actions, personalised treatment, risk indicators and electronic patient information leaflets. The anticipated acceptability and intention to use the KSS, was moderate to high. While time was identified as a cost/burden, this was outweighed if the KSS improved patient outcomes and increased prescribing confidence. All the main requested features were implemented in the KSS except personalised treatment recommendations because current treatment guidelines do not cover all of the frequently encountered clinical challenges, such as frequent repeat antibiotic prescribing shortly after the first one.

### Study design, participating sites and overview of interventions

The study will be a pragmatic cluster randomised controlled trial with 1:1 randomisation of practices to:

1. Periodic feedback to general practices using practice-level+individual prescriber feedback dashboards. Online supplemental appendix 1 provides examples of different dashboards.
  or
2. Practice-level+individual prescriber feedback dashboards+KSS during consultation with the clinician providing content (such as an information sheet) that is personalised to the patient.

10.1136/bmjopen-2023-076296.supp1 Supplementary data

The study sites will include general practices located in the North-West of England that provide patient-level data to a regional shared care record system (https://www.graphnethealth.com), which is deployed in Greater Manchester, Wirral and Cheshire/Merseyside regions, together covering a 5.4 million general population. The shared care record collates data from various NHS organisations to support direct patient care, service planning and research. Researchers access anonymised patient-level data through a secure data analytic environment linked to the shared care records.

### Details of study interventions

#### Content of KSS for clinicians

**Figure 1:**
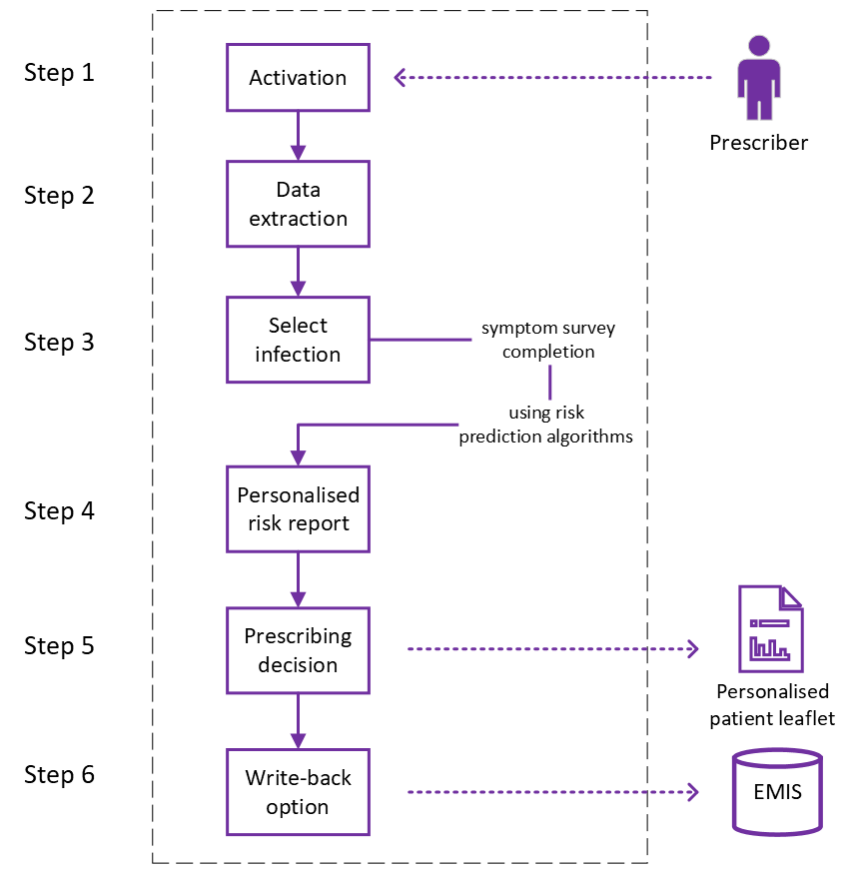
Architecture of the knowledge support system.

**Figure 2:**
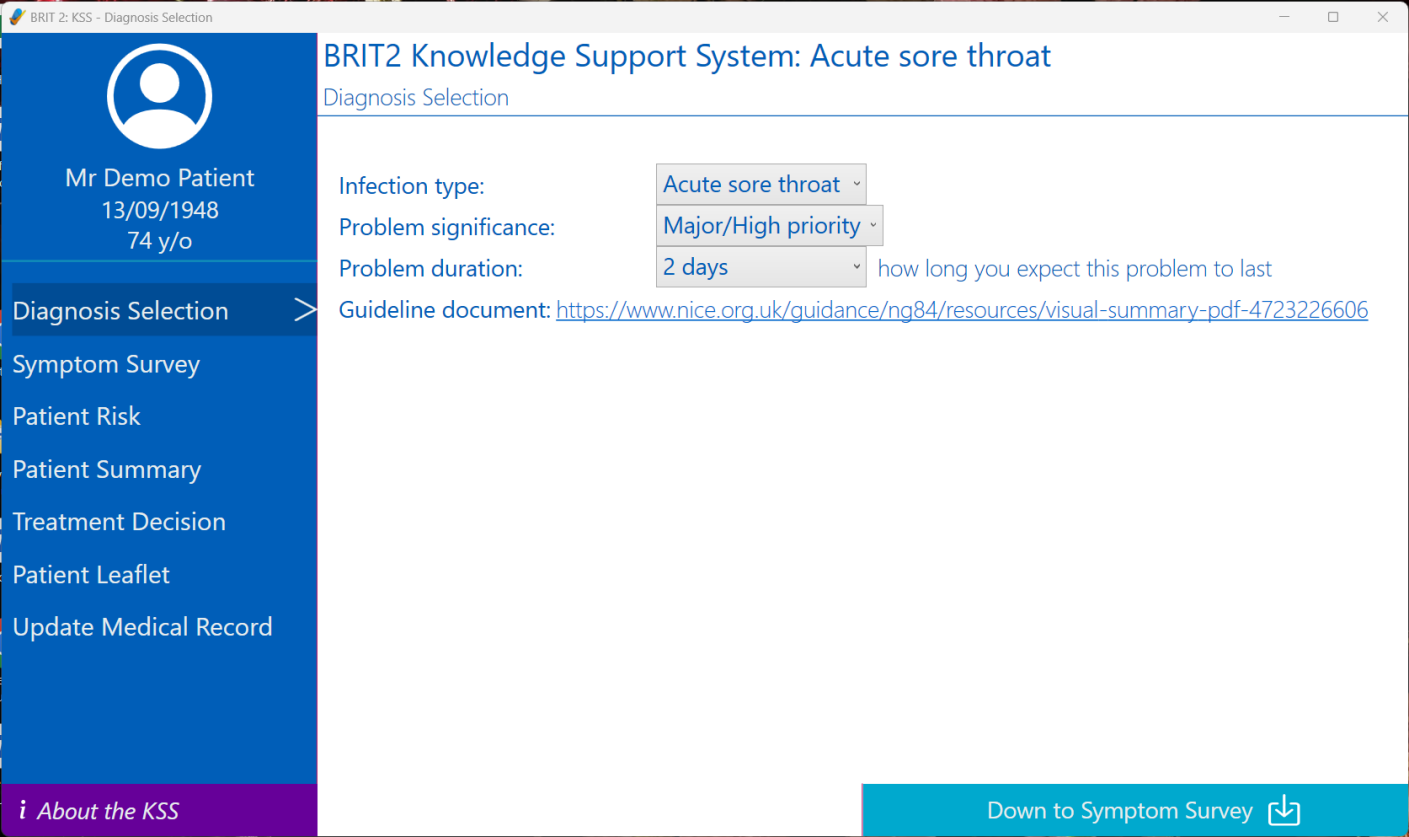
Example of a KSS screen after activation within the care record system during consultation highlighting the patient’s details (fictional) with the active record and the various screen available to the clinician. KSS, knowledge support system.

Figure 1 shows the architecture of the KSS including data extracted from the patient’s EHR, ability for clinicians to enter details on infection severity, estimation and presentation of risk scores, generation of personalised patient leaflet and write-back of codes to the EHR. The KSS will be available for all clinicians in the practices randomised into the KSS arm of the study. Eight screens are accessible within the KSS. Figure 2 shows screen 1 of the KSS after activation (and online supplemental appendix 2 shows additional examples of KSS screenshots). This provides for the selection of the type of common infection and indicators of problem significance and duration. Screen 2 is the symptom survey which provides a survey with list of symptoms to capture infection severity. An example is the Feverpain score for acute sore throat, including questions such as fever in past 24 hours, presence of cough or coryza and physical examination findings.^11^ Screen 3 (patient summary) includes an extraction of relevant information as recorded in the patient’s EHR: the data on recent antibiotic prescribing (dates and types) and information on presence of allergies. This screen is populated automatically after KSS activation. Screen 4 (patient risks) gives personalised information including risks of:

10.1136/bmjopen-2023-076296.supp2 Supplementary data

1. Developing infection-related complications that lead to hospital admission.
2. Resistance based on number of prior antibiotics prescribed to the patient in the previous 12 months.
3. Antibiotic failure indicating probability may be prescribed another antibiotic within 30 days
4. Severe adverse outcomes such as hospital admission for renal failure.

These personalised risk scores are based on risk prediction models including characteristics such as age, gender, clinical and medication risk factors, ethnicity and socioeconomic status. These risk prediction models were developed and tested in the OpenSAFELY platform (https://opensafely.org) or the Clinical Practice Research Datalink GOLD and Aurum (https://cprd.com/).^12 13^ Risks are calculated using these algorithms which are combined with the patient’s specific characteristics as recorded in the EHR. The clinician is given links to the relevant National Institute for Health and Care Excellence (NICE) guideline for the infection in screen 5 (treatment guidelines). The clinician can then consider these risk scores alongside other information when considering whether to prescribe an antibiotic, line up a back-up antibiotic or discuss with the patient alternative care pathways without antibiotics (treatment decision is recorded in screen 6). Once the shared decision-making process is complete, the clinician may provide an information sheet for the patient, particularly where an antibiotic is not being prescribed to advise the patient of the decision and provide the personalised risk scores (screen 7 with patient leaflet). Screen 8 allows the clinician to initiate a write-back function which will write back to the care record the diagnosis, symptoms and treatment as SNOMED codes. The KSS has been developed for use with EMIS which is the most common primary care record system in England.^14^ Patient records remain confidential, and all data flows happen within the NHS.

#### KSS content for patients

We developed personalised patient information sheets that show tailored risk/benefit information based on patient-specific estimates of for example the risk of being admitted to hospital for infection-related complications. These information sheets are populated by the results of the KSS activation. The clinician can provide these to patients after consultation, in addition to any existing generic patient information sheets (TARGET) which also include information on illness duration, self-care advice, prevention advice and advice on when to re-consult (https://www.rcgp.org.uk/clinical-and-research/resources/toolkits/amr/target-antibiotics-toolkit/leaflets-to-share-with-patients.aspx). Our advisory group including members of the public were involved in discussing and reviewing draft information sheets.

### Sample size

The KSS arm will be limited to a maximum of 62 practices as this arm involves time and effort of clinicians during consultation. Sample size calculation has been computed based on the primary outcome of practice rate of antibiotic prescribing (per 1000 patients). In CPRD, the mean practice list size was 7981 and the overall rate of the number of antibiotic prescriptions (for any indication) was 598 per 1000 patients per year (SD 155). Assuming a reduction of 10% in the low-intensity KSS arm, its antibiotic prescribing rate will be around 538 per 1000 patients per year. Assuming an unchanged SD of 155 and practice attrition rate of 5%, randomising 62 practices to each of the two arms will provide 90% power to detect a 10% (54 per 1000 patients per year) between-arm difference in the overall rate of antibiotic prescribing (two-sided α=0.05), assuming a correlation of 0.82 between baseline and outcome antibiotic prescribing rate and analysing using analysis of covariance with baseline prescribing rate as covariate and 5% drop-out rate. For 80% power, 47 practices are needed in each arm. Sample size calculations were performed in PASS 2019 software. Our primary target is 112 practices although we will recruit up to 124 practices, given the limitation on numbers in the KSS arm. However, determination of the total sample size of practices will depend on practice consent to take part in the KSS evaluation and passing an assessment of IT system capability for the KSS installation.

### Study outcomes

The unit of analysis will be general practice. The primary clinical outcome will be the overall rate of antibiotic prescribing. One secondary outcome, relating to safety, will be infection-related complications as recorded in the primary care record, including pneumonia and lower respiratory tract infections, peritonsillar abscess, mastoiditis, intracranial abscess, empyema, scarlet fever, pyelonephritis, septic arthritis, osteomyelitis, meningitis, toxic shock syndrome and septicaemia, and Lemierre syndrome (as defined by Gulliford *et al*^15^). BRIT found that clinician-recorded infection-related complications provided comparable results as those recorded in linked hospital admission data.^16^ Another safety outcome will be hospital admissions for infection-related complications (linked hospital data are likely to be available within the shared care-record systems). The primary and safety-related secondary outcomes are equivalent to those used in a recent trial in UK primary care of a decision support system plus patient information sheets.^17^ Another secondary outcome will be the level of risk-based prescribing (ie, the proportion of antibiotic prescribing in patients with different risks) as based on the risk prediction scores as developed and validated in the BRIT project.^16^

A Statistical Analysis Plan (SAP), to be approved by the Steering Committee, will detail the planned analyses. Data analysts will have access to anonymised data from participating practices within a secure data analytic environment. Rates will be analysed using appropriate models with Poisson or normal error structures, weighted by practice size, and with adjustment for practice-level covariates including region (Greater Manchester, Wirral and Merseyside/Cheshire), study quarter, period of randomisation and baseline antibiotic prescribing, socioeconomic status, case-mix of patients and ethnicity distribution in each practice (including Charlson comorbidity score^18^), consultation rates for common infections, coding propensity and characteristics of patients with common infections. The coding propensity will be the proportion of antibiotic prescriptions with recent clinical record for common infection. The characteristics of patients with common infections will include the averages in each practice for mean age, sex, and predictors for infection-related complication (including clinical and medication risk factors^16^). Body mass index and smoking history will also be considered as potential predictors for infection-related complications; these will be discussed with the Study Steering Committee and will each be included in either the primary analysis, a sensitivity analysis or not included in the analysis. The primary analysis will be a complete case analysis (ie, excluding practices who drop out of the trial), unless attrition is higher than expected (ie, >10% overall or differential between-arm attrition of >10%).

#### Cost-effectiveness

NICE has produced guidance on the type of economic analysis needed for digital health technologies, depending on the level of financial risk to the NHS.^19 20^ For a digital health technology like KSS that has the potential to be cost-saving, the economic analysis level could be defined as ‘low financial commitment’ requiring at least a cost-consequence analysis (CCA) and a budget impact analysis (BIA). We will estimate the economic impact of the KSS, from the perspective of the NHS and partner social care services The primary analysis will be a within-trial CCA and BIA where the outcomes are overall rate of antibiotic prescribing and level of risk-based prescribing. As we do not have access to infection-related hospital admissions, we need to use a proxy for this aspect of resource use. Therefore, we will also carry out an indicative BRIT2 algorithm-based economic analysis where we will use the data summarised above plus estimates of the expected rate of infection-related hospital admissions based on the validated BRIT2 risk algorithms, to provide indicative estimates of overall costs associated with KSS.

In the primary CCA, we will use the primary outcome, overall level of antibiotic prescribing, level of risk-based prescribing, infection-related hospital admissions and infection-related complications, to estimate economic impact of KSS.

### Pilot phase

A pilot phase will be conducted, with the initial recruitment of 20 practices in two regions. In case of the trial not meeting this target, the study is to be terminated writing up feasibility and lessons learnt without major statistical or pharmacoeconomic analyses. If the pilot target is met but the study recruits less than 94 practices, statistical or pharmacoeconomic analyses will be limited due to power.

### Allocation and blinding

Allocation to interventions will use anonymised practice identifiers with randomisation stratified by region (Greater Manchester, Wirral and Merseyside/Cheshire) and baseline rate of antibiotic prescribing. The project manager will recruit practices and randomly allocate using https://www.sealedenvelope.com. The data analysts will not be blinded to intervention allocation. However, the SAP for the end-of-study analyses will be developed by the Lead Statistician from the Centre for Biostatistics who will not have access to study data and practice names throughout the study, and he will also perform the checking of statistical results to ensure that they are performed in accordance with the approved SAP.

### Other study-related activities

#### Online community of practice and professional training

An online community of practice (OCoP), also known as a virtual community of practice, will also be implemented for interested practice prescribing advisors and clinicians to share knowledge through education on the challenges within their clinics and the opportunities to improve prescribing. The OCoP will provide a critical resource to professionals who want and need recommendations, pointers, tips and tricks, best practices, insights and innovations for optimising prescribing.^21 22^ The purpose of this OCoP is to facilitate dialogue among experts and clinical stakeholders, present analysis results in order to collectively discuss the local challenges and opportunities for improving antibiotic prescribing that is relevant to the user.

The OCoP will focus on discussion of complex cases (eg, patients who frequently but intermittently are prescribed antibiotics). It will also discuss the analytics of the local data and any actions that need to be implemented and evaluated locally—this will help researchers to understand the interpretation of the dashboard and provide feedback on the dashboard. The target users will be health professionals in the participating general practices as well as local healthcare (NHS) and public health organisations. The staff will include general practitioners (GPs), GP research leads, practice managers, quality and safety pharmacists, medicines optimisations pharmacists and public health consultants. We have already spoken to some of the community to develop this and when developing the OCoP we will build on this to provide a platform that will suit the way this community works. We need some champions to encourage widespread adoption of the resource. We will include some workshops using video conferencing to gain some of the input for the OCoP as well as provide training.

### Ethics and dissemination

#### Ethics approval

Research Ethics Committee approval has been obtained (IRAS 290050). The study will be conducted in full conformance with all relevant legal requirements and the principles of the Declaration of Helsinki, Good Clinical Practice and the UK Policy Framework for Health and Social Care Research 2017.

### Publication policy

Dissemination of research outputs to participating clinical staff and policy and guideline developers will be an integral part of this project. In addition, we will promote dissemination to national policy makers, managers and clinical leaders, through project summaries and policy briefings. All study results will be reported in accordance to this study protocol and to the Consolidated Standards of Reporting Trials statement extended to cluster randomised trials.^23^ The study sponsor and funder do not have any role in data collection, management, analysis and interpretation of data; writing of the report; and the decision to submit the report for publication.

### Patient and public involvement

Patients and/or the public were involved in the design, or conduct, or reporting, or dissemination plans of this research. Participatory workshops, where stakeholders are brought together to seek their opinions, extract knowledge and solve problems, were used to inform the design of the protocol. In addition, members of the public interested in health data research were invited through advertisement to the National Institute for Health Research (NIHR) patient research ambassador network, to form an advisory group. This group has been meeting four times throughout the course of the research with a focus on patient risk communications. Members were also invited to rotate into the Trial Steering Committee and research team meetings to add value and report back to the public advisory group.

### Steering Committee

We have established an oversight committee, the Study Steering Committee, including senior representatives independent of the trial, comprising stakeholders in general practice, pharmacy, commissioning groups and patient representatives (6-monthly meetings) and statistician. Professor Janusz Jankowski, clinician and healthcare policy expert, chairs this committee. We follow NIHR guidance for programme steering committees (Research Governance Guidelines (nihr.ac.uk)).

### Consent

General practices will need to agree with study participation (information sheet is provided in online supplemental appendix 3) and clinicians will need to activate the KSS within the care record system. Patient consent for use of the KSS will not be sought and is not required as approved by the Ethics Committee. The reason for this is that the KSS intervention is focused on clinicians—the KSS will provide clinicians with further information. It will be up the clinician to decide whether to access or not the KSS, prescribe an antibiotic or not. The KSS makes information easily accessible to the clinicians and will not provide treatment recommendations. Studies very similar to our proposal also did not seek informed consent from patients, but, like our study, required consent by participating clinicians.^17 24^

10.1136/bmjopen-2023-076296.supp3 Supplementary data

### Device classification of the KSS

The UK regulator Medicines and Healthcare products Regulatory Agency (MHRA) produced guidance on how to comply with the legal requirements for interventions including software. Based on a reasonable interpretation of the MHRA guidance, BRIT2 is not considered a device for the following reasons: the KSS will not apply ‘automated reasoning’ to the clinician’s decision to prescribe an antibiotic or not. There will be no ‘if then reasoning’, no direct inferences can be drawn from the BRIT2 information provided. There will be no provision of a treatment threshold; it will be up to the clinician to decide whether a BRIT2 risk estimate of infection-related complications of for example, 1% is relevant to the patient and whether this would require an antibiotic. BRIT2 will provide ‘reference information to enable a clinician to make a clinician decision’.

### Technical description of the KSS

The initial version of the KSS has been written to target the EMIS Health’s EMIS Web EHR system as EMIS Health provide EHR services to the majority of general practices in the trial region. This was a strategic decision aimed at controlling the complexity of the software and managing the delivery schedule while maximising the potential targets for the KSS randomisation. Future iterations of the KSS will be platform agnostic and present data in the presentation layer of the application in a consistent way regardless of EHR software running on-site and early design decisions support this future requirement.

### Compliance to Information Standards (Data Coordination Board [DCB] standards)

As the KSS is to be used to support the real-time direct care of patients, a rigorous clinical risk assessment was conducted to ensure compliance with the DCB0129 standards. As developed by NHS Digital, this standard ‘provides a set of requirements suitably structured to promote and ensure the effective application of clinical risk management by those organisations that are responsible for the development and maintenance of Health IT Systems for use within the health and care environment’ (https://digital.nhs.uk/data-and-information/information-standards/information-standards-and-data-collections-including-extractions/publications-and-notifications/standards-and-collections/dcb0129-clinical-risk-management-its-application-in-the-manufacture-of-health-it-systems). On evidence of compliance with the DCB0129 standards, approval was granted by EMIS to establish an API connection between KSS and the live patient record.

## Supplementary Material

Reviewer comments

Author's manuscript
