# Supplementary material for: Knowledge support for optimising antibiotic prescribing for common infections in general practices: evaluation of the effectiveness of periodic feedback, decision support during consultations and peer comparisons in a cluster randomised trial (BRIT2) – study protocol
Source: BMJ Open. 2023 Aug 22;13(8):e076296. doi: 10.1136/bmjopen-2023-076296 (PMC10445367; doi:10.1136/bmjopen-2023-076296)
Supplement: Supplementary data [file bmjopen-2023-076296supp001.pdf]

Appendix 1: Examples of dashboards as developed in BRIT project

Benchmarking

Data from large national data sources have been and will be used to get better understanding of the drivers for heterogeneity in care and for the effectiveness of different treatment strategies. The results will provide the baseline content for the dashboards.

A previous project piloted and implemented the IT infrastructure for the Learning Healthcare System (i.e., data analytics, feedback to clinicians) on antibiotic prescribing care in UK primary care, This project (BRIT: Building Rapid Interventions to reduce AMR and over-prescribing of antibiotics) was part of the £20 million Department of Health & Social Care (DHSC) funded Connected Health Cities programme (https://www.connectedhealthcities.org/). The data analytics activities in BRIT consisted of benchmarking current practice in primary care, evaluating the levels of suboptimal antibiotic prescribing and identifying opportunities for improvement. Large variability in antibiotic prescribing was observed between practices and within practices: Change points in prescribing did not reflect updates to national guidelines. Prescribing levels within practices were not consistent for different infectious conditions. BRIT also found high levels of prescribing of potentially inappropriate type of antibiotics which were highest for otitis externa (67.3%) and upper respiratory tract infection (38.7%). BRIT found that that over the last 15 years antibiotic prescribing in primary care was not risk-based: patients with very low risk of infection-related hospital admissions were as likely to receive an antibiotic as patients with higher risks. BRIT also evaluated the effectiveness of treating common infections with antibiotics. The findings also indicate that incidental use of antibiotics is effective in reducing infection-related hospital admissions while repeated courses of antibiotics may have limited benefit and be indicative of adverse outcomes. Of 5.1 million antibiotics prescribed in UK primary care, only 14.8% were given to patients without any antibiotic prescribing in the previous three years and 43.6% are for patients who already received 5+ antibiotic prescriptions in the previous three years. These BRIT findings indicate that optimal antibiotic prescribing in primary care is a complex interplay of a patient's symptoms, age and co-morbidity and previous history of antibiotic use. While incidental use of an antibiotic may reduce the risk of infection-related complications, it may also decrease the effectiveness of the antibiotic for future infections (possibly due to the development of resistance in the patient [1]). This highlights the importance for patient-specific communication, information and algorithms that recommend best course of action.

Recent research found considerable variability between GPs in the case mix of patients consulting and large variability in antibiotic prescribing habits. The majority of clinicians (> 95%) prescribed at least one antibiotic measure that was above the medians of their peers. It concluded that there is a need for a wider range of objectives (using a variety of measures without ranking of clinicians based on a single metric), varying engagement strategies with feedback tailored to each clinician, local context including bespoke

recommendations that could be implemented and proactive support from colleagues and local organisations [2].

The LHS approach is not just about analysis of data and feedback of results to clinicians. An important aspect is around understanding and changing behaviours which is a focus of behavioural sciences [3]. An example of this is the provision of social norm practice-level feedback (i.e., comparing a clinician to other peers). In a UK cluster randomised controlled trial (cRCT), this approach was found to substantially reduce antibiotic prescribing. Every GP in the feedback intervention group was sent a letter from England's Chief Medical Officer [4]. A US cRCT tested the effects of several behavioural interventions and found that peer comparisons resulted in lower rates of inappropriate antibiotic prescribing for acute respiratory tract infection. These comparisons consisted of emails sent to clinicians that compared their antibiotic prescribing rates with those with the lowest inappropriate prescribing rates [5].

The BRIT dashboard summarises information on antibiotic prescribing by infection type and where these prescriptions may deviate from the recommended guidelines, overall prescribing rates with comparision to peers, as well as how a practice may prescribe based on a pratients risk of an infection-related complication. On the hompage users have the option to clickthe lage icons or use the menu bar across the top to navigete to each dashbord.

The first group of visualisations (Supplementary Figure 1A) shows the general practices (GPs) inforation about their antibiotic prescribing by infectious conditions. The first part of the dashboard shows colourful notification boxes with the number of infection-related consultations in a given time period (yellow box), the number of antibiotic prescriptions issued for these consultations (blue box), the number of these prescriptions issed that deviated from the national recommended guidelines (red box) and a nudging notification box (in green) that suggests areas for improvement in their prescribing based on which condition saw more deviating prescriptions. Users reviewing the dashboard, such as prescribing advisor may decide to concentrate their efforts when auditing the practices antibiotic prescribing in a given month by delving deeper into the prescriptions issued for consultations were the system flagged the most inappropriate prescriptions (in this example upper respiratory tract infections; URTI). Next the user will see a box and whisker plot showing national prescribing rates by infection, i.e. for each practice the percentage of consultations that resulted in an antibiotic prescription by infection type (Supplementary Figure 1B). The users of the BRIT dashboard can also see where their practice sits (the black dots overlaid on this plot) compared to the national prescribing rates. For example, Supplementary Figure 1 shows the practice has a higher prescribing rate for breast-related infections compared to the national average but have a lover prescribing rate for ear-related infections (otitis externa and otitis media), highlighting which infections warrant further investigation in their practice. The practice can also see what prescriptions are being prescribed to each infectious condition as a percentage in a sunburst plot, where Supplementary figure 1C shows that when selecting lower respiratiory tract infections

(LRTI) over three quarters of the prescriptions their practice prescribed for this condition were fore amoxicillian, followed by doxycycline and clarithromycin as well as other antibiotic less commonly prescribed to these conditionsn in their practice in a given time period.

The practice can also see how their practice is performing in terms of prescribing appropriateness (Figure 1D). The analysis behind these plots assess wheather each antibioitc prescription in agreement (blue) or disagreement (red) with the recommended first- second- or alternative- medicine. For example this plot shows a hypothetical practice were 24% of the consultations for sinusitis resulted in an antibiotic prescription that may have been inappropriate because it deviated form the recommended guidelines. They can also see for each condition what antibiotic type was issued and flagged as potentially inappropriate (figure not shown). If they were to scroll down on the sape dashboard there are snapshots of the most recent antibiotic guidelines , enabiling a quick comparision of the antibiotics they have prescribed deviating from recommendations and refreshing what antibiotics are currently recommended appropriate for each condition. Again, this analysis highlights areas for review/improvement specific to each practice that may instigate discussion in a practice manegement meeting ensuring prescribers have refreshed their knowledge on the current prescribing guidelines for a particular infection.

A second topic of analysis is looking at how well a practice prescribed based on a patients predicted risk of a poor outcome (e.g., infection-related hospitalisation). The analysis is based on a validated risk prediction model using two national datasets . Supplementary Figure 2 shows the predicted risk (using the prediction model) for every patient presenting with a particular infection (in this case LRTI) in a given time period (6 months), separated into categories from very low predicted risk to very high predicted risk of complication. For each category the actual prescribing rate is caluculated as a percentage and displayed by the bule bars. Practices prescribing according to patients risk of complication would expect to see the height of the blue bars match the red line here, where very low risk patients received prescriptions on occasion, perhapse due to the severity of their symptom and the very high risk patients are receiving a prescription more often. However, this figure shows a flat line across all risk categories again suggesting prescribing for this infection is not well targeted to high risk patients and there is room for improvement. The dashboard also has a built in a risk prediction calculator, allowing users modify various patient characteristics and see which effects the predicted risk more, again adding a training element to the dashboard that can help to identify ways to further optimise their prescribing.

The final topic of analysis is prescribing benchmarking. The traffic-light visulisation (Supplementary figure 3A) shows STAR-PU adjusted prescriing rates for each practice. This is an age-sex adjusted prescribing rate allowing fair comparison between practices. The yellow dots show bottom 25% of practices with a relatively low prescribing rate, the red dots show the top 25% of high prescribing practices and the green is the middle 50% percent of practices with an average prescribing rate. The black dot then shows where the individual

practice sits compared to their peers. The bottom figure (Supplementary figure 3B) shows the practices prescribing rate over time. This is important compare how a practice is prescribing to their peerd but to also observe any rapid changes to their prescribing rates following an intervention; allowing rapid review and uptake of interventions that work and removal of those that do not. The practice can also use these sort of comparison plots to see seasonality trends as well as changes in prescribing due to an outbreak of a pandemic disease, such as COVID-19. Here we can see the prescribing rate in April 2018 and April 2019 for this demostrative practice was 31.5 units of antibiotics for every 1000 registered patients; however following the outbreak of COVID-19 the prescribing rate in April 2020 has reduced to 23.85 units of antibiotics prescribed per 1000 registered patients.

The data in the dashboard is regularly updated providing practices with frequent incited to their own prescribing and how changes they make can alter and optimise their prescribing. The content of the dahboard visualisations also evolving in an iterative manner based on feedback from users and key stakholders, creating a learning health care system that is evolving with the health care our patner practices provide.

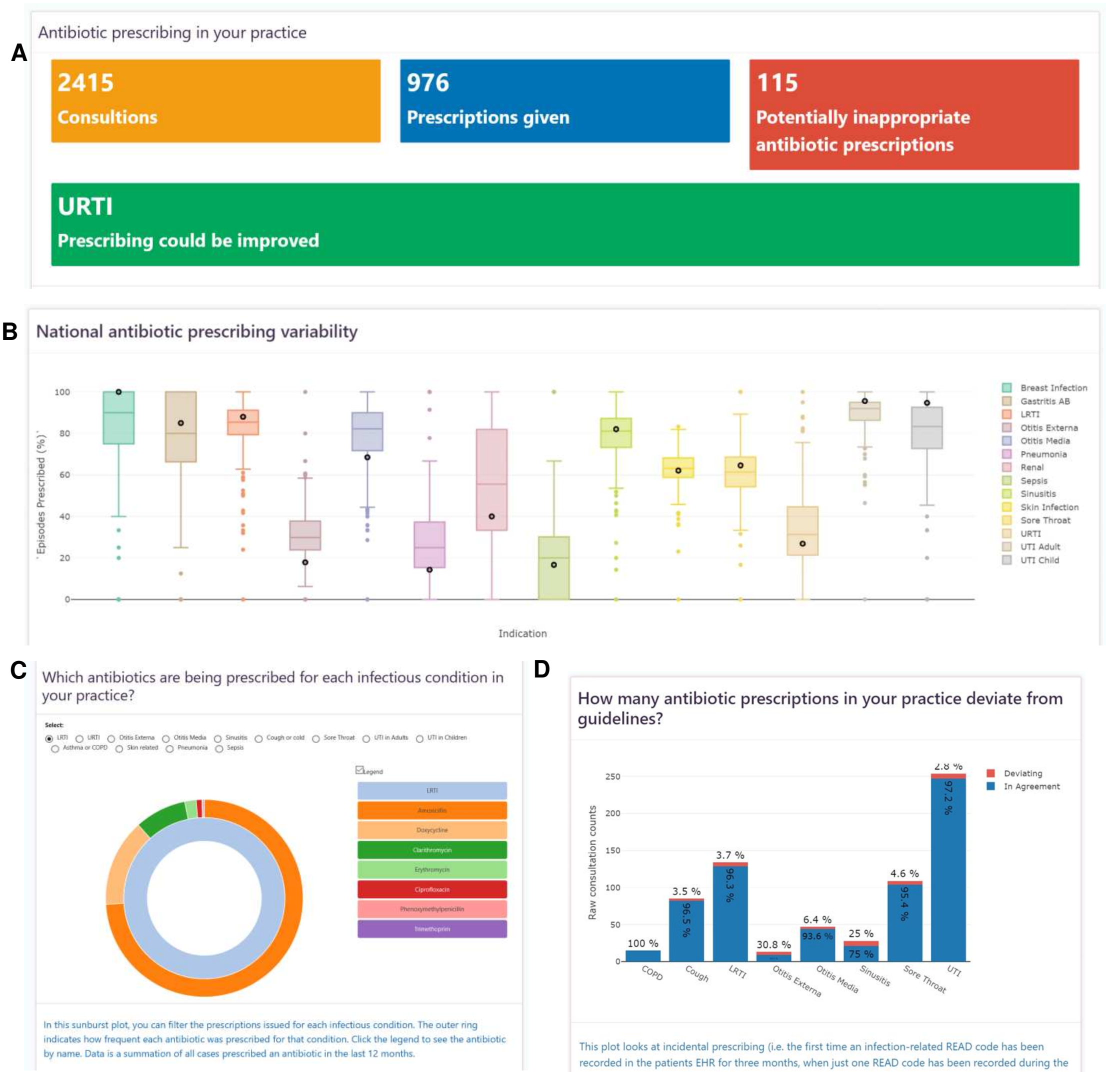

Supplementary Figure 1: Examples of dashboards providing periodic feedback on antibiotic prescribing of practices

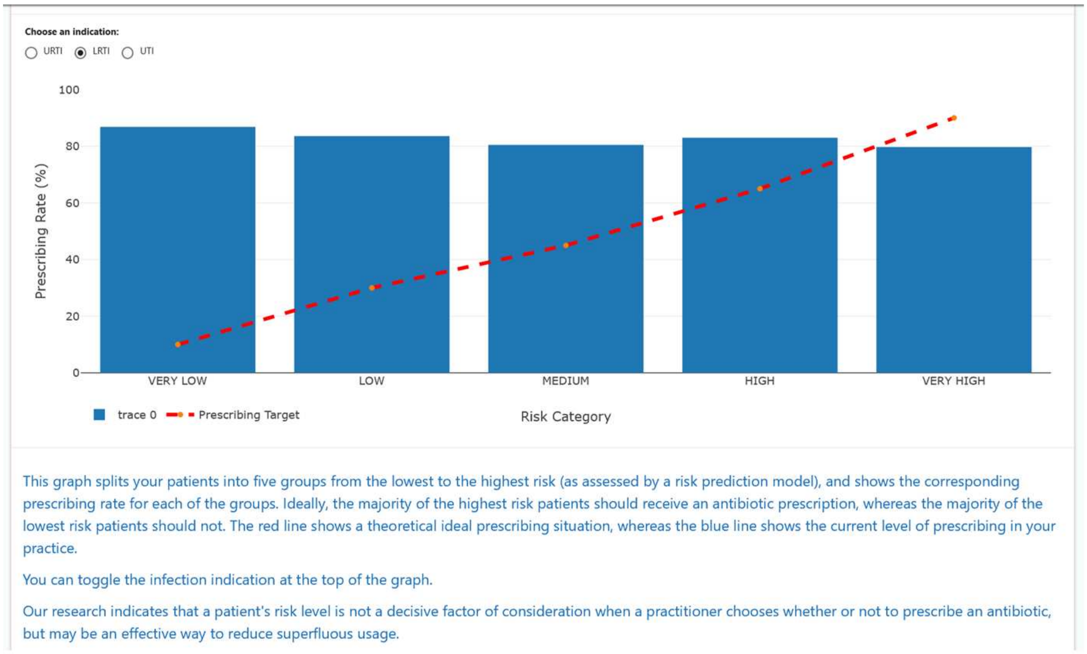

Supplementary Figure 2: dashboards of antibiotic prescribing by patient risk profile

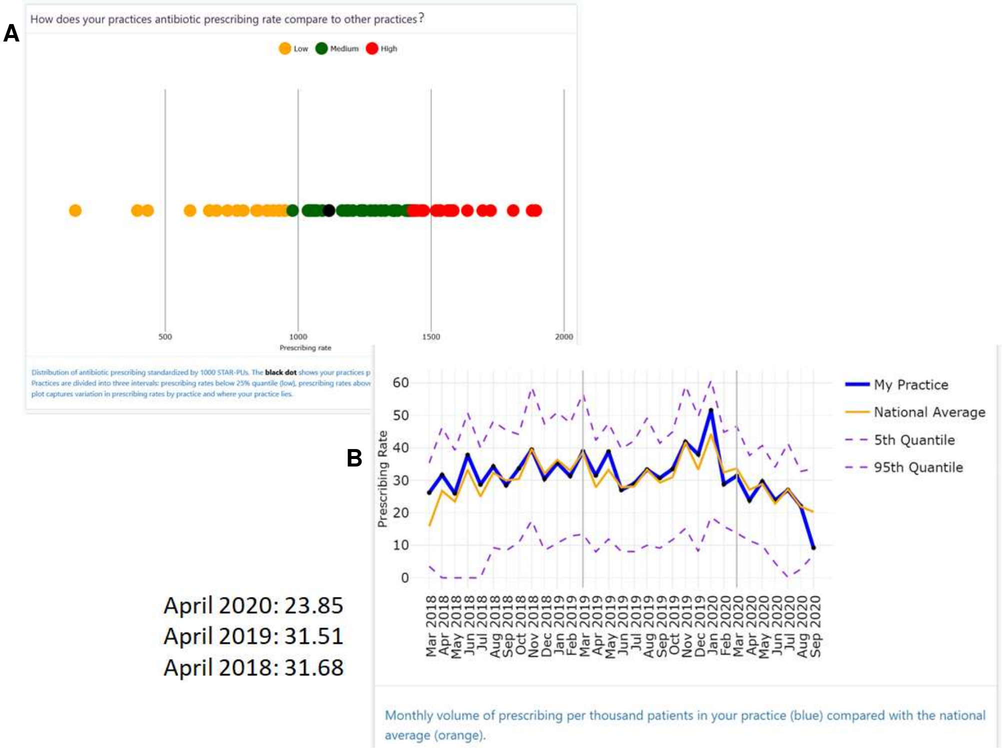

Supplementary Figure 3: Dashboards of practice-level antibiotic prescribing rates

References

1. Costelloe C, Metcalfe C, Lovering A, Mant D, Hay AD. Effect of antibiotic prescribing in primary care on antimicrobial resistance in individual patients: systematic review and meta-analysis. BMJ [Internet]. 2010 [cited 2018 Sep 15];340:c2096. Available from: http://www.ncbi.nlm.nih.gov/pubmed/20483949
2. Van Staa T, Li Y, Gold N, Chadborn T, Welfare W, Palin V, et al. Comparing antibiotic prescribing between clinicians in UK primary care: an analysis in a cohort study of eight different measures of antibiotic prescribing. BMJ Qual Saf [Internet]. BMJ Qual Saf; 2022 [cited 2022 Sep 6];bmjqs-2020-012108. Available from: https://pubmed.ncbi.nlm.nih.gov/35241573/
3. Improving people's health: Applying behavioural and social sciences to improve population health and wellbeing in England [Internet]. 2018. Available from: www.facebook.com/PublicHealthEngland
4. Hallsworth M, Chadborn T, Sallis A, Sanders M, Berry D, Greaves F, et al. Provision of social norm feedback to high prescribers of antibiotics in general practice: a pragmatic national randomised controlled trial. Lancet [Internet]. 2016 [cited 2018 Sep 15];387:1743–52. Available from: http://www.ncbi.nlm.nih.gov/pubmed/26898856
5. Meeker D, Linder JA, Fox CR, Friedberg MW, Persell SD, Goldstein NJ, et al. Effect of behavioral interventions on inappropriate antibiotic prescribing among primary care practices a randomized clinical trial. JAMA - J Am Med Assoc. American Medical Association; 2016;315:562–70.
