# Supplementary material for: Knowledge support for optimising antibiotic prescribing for common infections in general practices: evaluation of the effectiveness of periodic feedback, decision support during consultations and peer comparisons in a cluster randomised trial (BRIT2) – study protocol
Source: BMJ Open. 2023 Aug 22;13(8):e076296. doi: 10.1136/bmjopen-2023-076296 (PMC10445367; doi:10.1136/bmjopen-2023-076296)
Supplement: Supplementary data [file bmjopen-2023-076296supp002.pdf]

**Appendix 2: Examples of KSS screens and personalised patient leaflet**

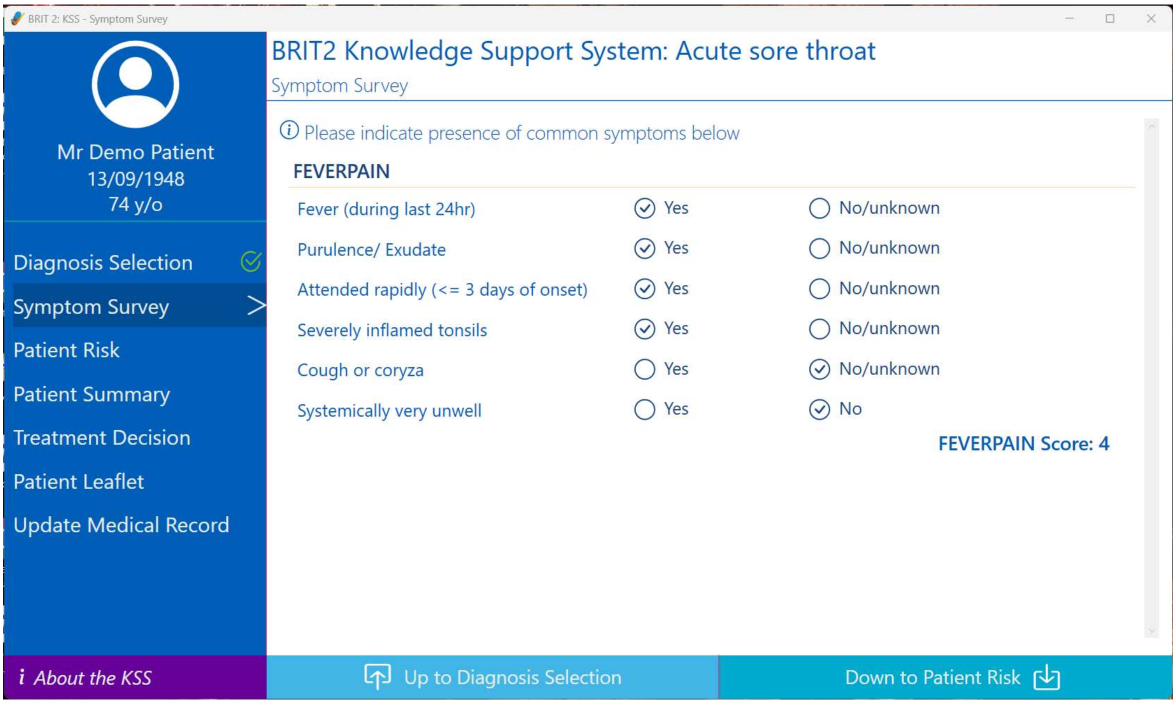

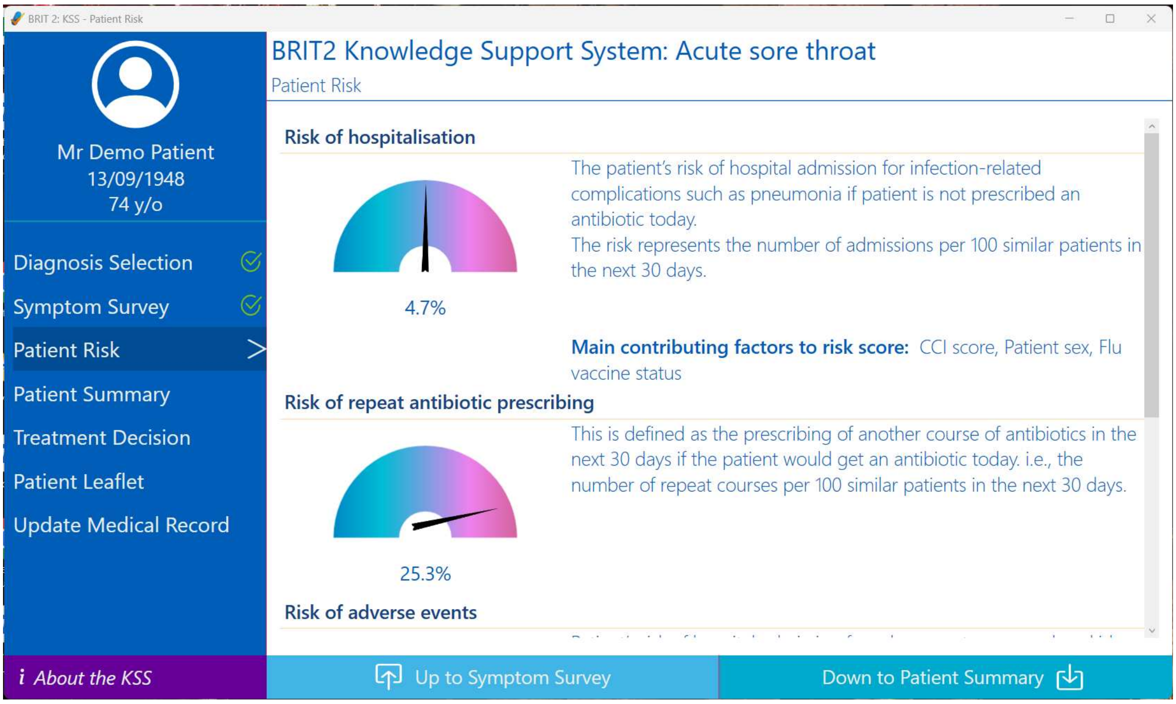
BRIT 2: KSS - Patient Risk
BRIT2 Knowledge Support System: Acute sore throat
Patient Risk
Mr Demo Patient
13/09/1948
74 y/o
Diagnosis Selection
Symptom Survey
Patient Risk
Patient Summary
Treatment Decision
Patient Leaflet
Update Medical Record
About the KSS
Risk of hospitalisation
4.7%
The patient's risk of hospital admission for infection-related complications such as pneumonia if patient is not prescribed an antibiotic today.
The risk represents the number of admissions per 100 similar patients in the next 30 days.
Main contributing factors to risk score: CCI score, Patient sex, Flu vaccine status
Risk of repeat antibiotic prescribing
25.3%
This is defined as the prescribing of another course of antibiotics in the next 30 days if the patient would get an antibiotic today. i.e., the number of repeat courses per 100 similar patients in the next 30 days.
Risk of adverse events
Up to Symptom Survey
Down to Patient Summary

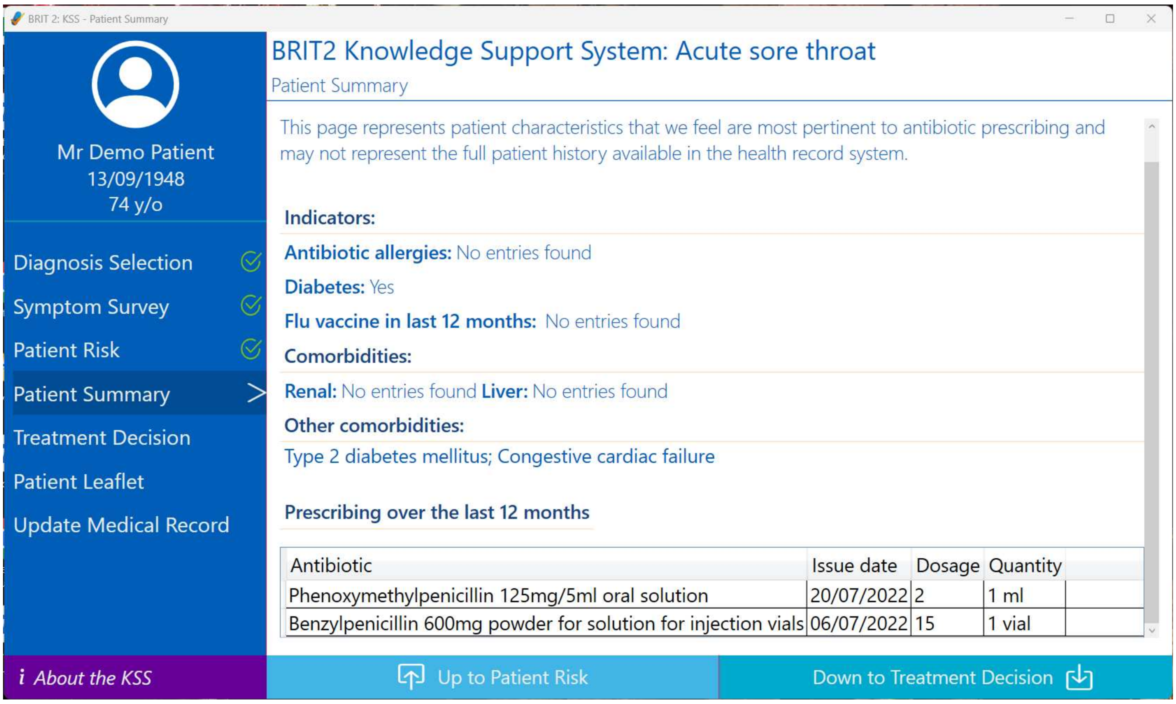
BRIT 2: KSS - Patient Summary
Mr Demo Patient
13/09/1948
74 y/o
Diagnosis Selection
Symptom Survey
Patient Risk
Patient Summary
Treatment Decision
Patient Leaflet
Update Medical Record
About the KSS
BRIT2 Knowledge Support System: Acute sore throat
Patient Summary
This page represents patient characteristics that we feel are most pertinent to antibiotic prescribing and may not represent the full patient history available in the health record system.
Indicators:
Antibiotic allergies: No entries found
Diabetes: Yes
Flu vaccine in last 12 months: No entries found
Comorbidities:
Renal: No entries found Liver: No entries found
Other comorbidities:
Type 2 diabetes mellitus; Congestive cardiac failure
Prescribing over the last 12 months
Antibiotic
Issue date
Dosage
Quantity
Phenoxymethylpenicillin 125mg/5ml oral solution
20/07/2022
2
1 ml
Benzylpenicillin 600mg powder for solution for injection vials
06/07/2022
15
1 vial
Up to Patient Risk
Down to Treatment Decision

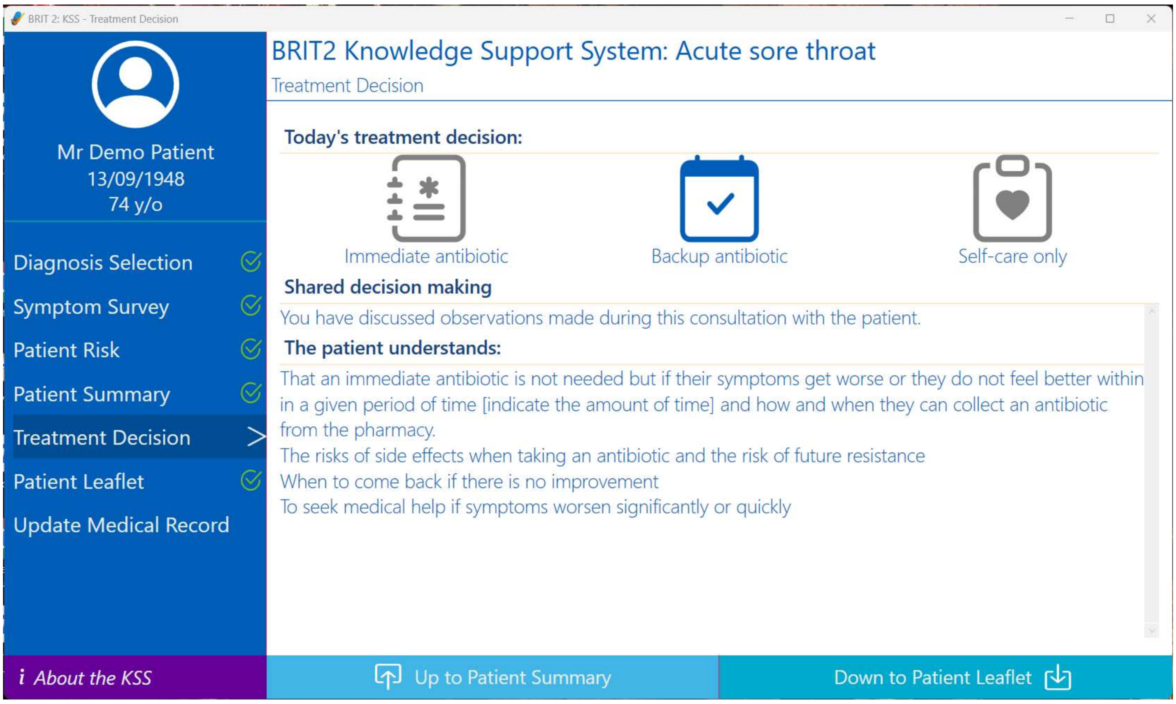
BRIT 2: KSS - Treatment Decision
Mr Demo Patient
13/09/1948
74 y/o
Diagnosis Selection
Symptom Survey
Patient Risk
Patient Summary
Treatment Decision
Patient Leaflet
Update Medical Record
BRIT2 Knowledge Support System: Acute sore throat
Treatment Decision
Today's treatment decision:
Immediate antibiotic
Backup antibiotic
Self-care only
Shared decision making
You have discussed observations made during this consultation with the patient.
The patient understands:
That an immediate antibiotic is not needed but if their symptoms get worse or they do not feel better within in a given period of time [indicate the amount of time] and how and when they can collect an antibiotic from the pharmacy.
The risks of side effects when taking an antibiotic and the risk of future resistance
When to come back if there is no improvement
To seek medical help if symptoms worsen significantly or quickly
i About the KSS
Up to Patient Summary
Down to Patient Leaflet

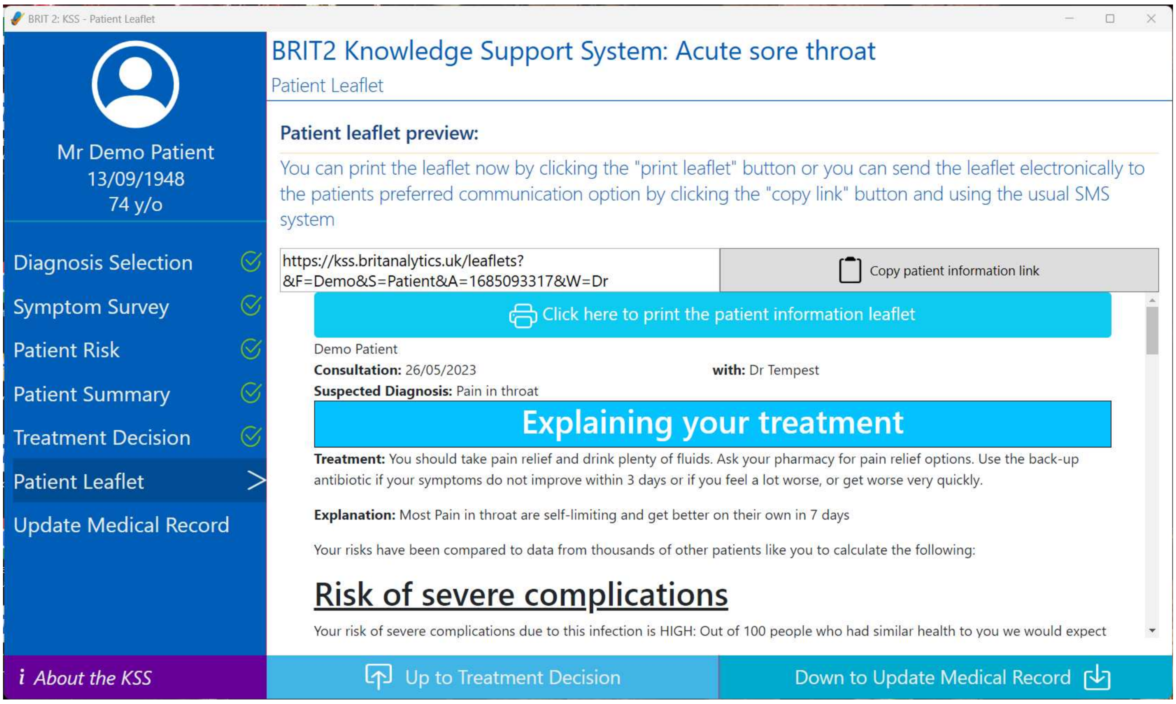
BRIT 2: KSS - Patient Leaflet
BRIT2 Knowledge Support System: Acute sore throat
Patient Leaflet
Mr Demo Patient
13/09/1948
74 y/o
Diagnosis Selection
Symptom Survey
Patient Risk
Patient Summary
Treatment Decision
Patient Leaflet
Update Medical Record
Patient leaflet preview:
You can print the leaflet now by clicking the "print leaflet" button or you can send the leaflet electronically to the patients preferred communication option by clicking the "copy link" button and using the usual SMS system
https://kss.britanalytics.uk/leaflets?&F=Demo&S=Patient&A=1685093317&W=Dr
Copy patient information link
Click here to print the patient information leaflet
Demo Patient
Consultation: 26/05/2023
with: Dr Tempest
Suspected Diagnosis: Pain in throat
Explaining your treatment
Treatment: You should take pain relief and drink plenty of fluids. Ask your pharmacy for pain relief options. Use the back-up antibiotic if your symptoms do not improve within 3 days or if you feel a lot worse, or get worse very quickly.
Explanation: Most Pain in throat are self-limiting and get better on their own in 7 days
Your risks have been compared to data from thousands of other patients like you to calculate the following:
Risk of severe complications
Your risk of severe complications due to this infection is HIGH: Out of 100 people who had similar health to you we would expect
i About the KSS
Up to Treatment Decision
Down to Update Medical Record

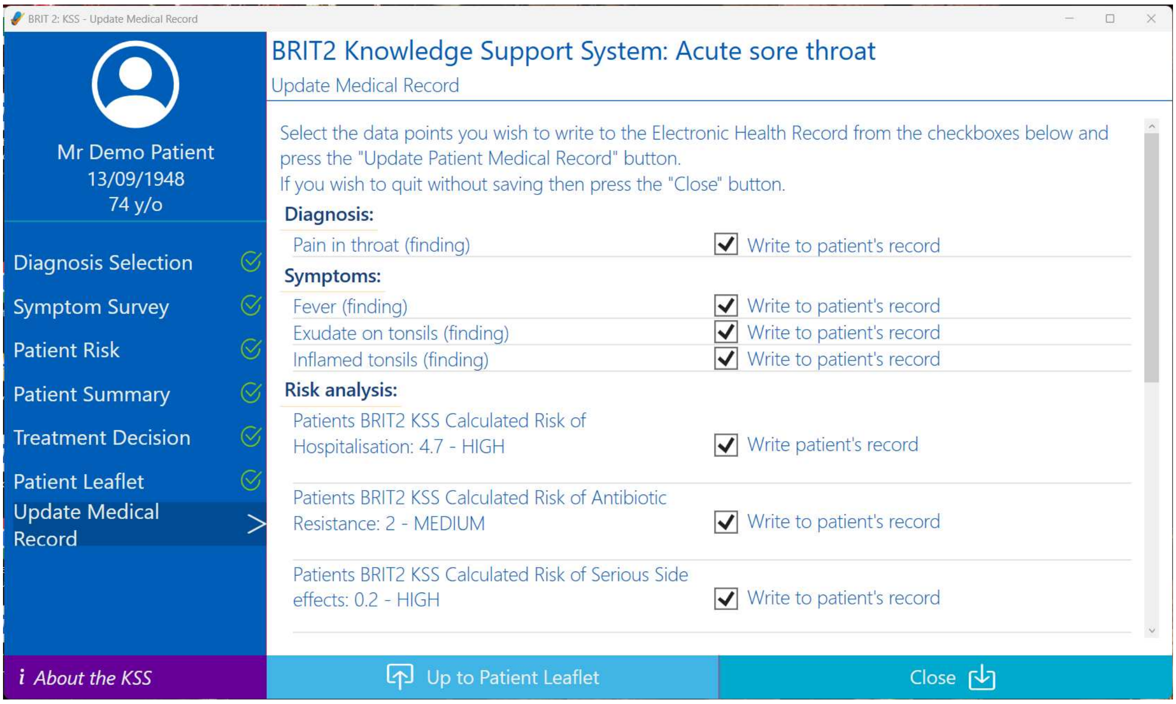
BRIT 2: KSS - Update Medical Record
Mr Demo Patient
13/09/1948
74 y/o
Diagnosis Selection
Symptom Survey
Patient Risk
Patient Summary
Treatment Decision
Patient Leaflet
Update Medical Record
About the KSS
BRIT2 Knowledge Support System: Acute sore throat
Update Medical Record
Select the data points you wish to write to the Electronic Health Record from the checkboxes below and press the "Update Patient Medical Record" button.
If you wish to quit without saving then press the "Close" button.
Diagnosis:
Pain in throat (finding)
Write to patient's record
Symptoms:
Fever (finding)
Write to patient's record
Exudate on tonsils (finding)
Write to patient's record
Inflamed tonsils (finding)
Write to patient's record
Risk analysis:
Patients BRIT2 KSS Calculated Risk of Hospitalisation: 4.7 - HIGH
Write patient's record
Patients BRIT2 KSS Calculated Risk of Antibiotic Resistance: 2 - MEDIUM
Write to patient's record
Patients BRIT2 KSS Calculated Risk of Serious Side effects: 0.2 - HIGH
Write to patient's record
Up to Patient Leaflet
Close

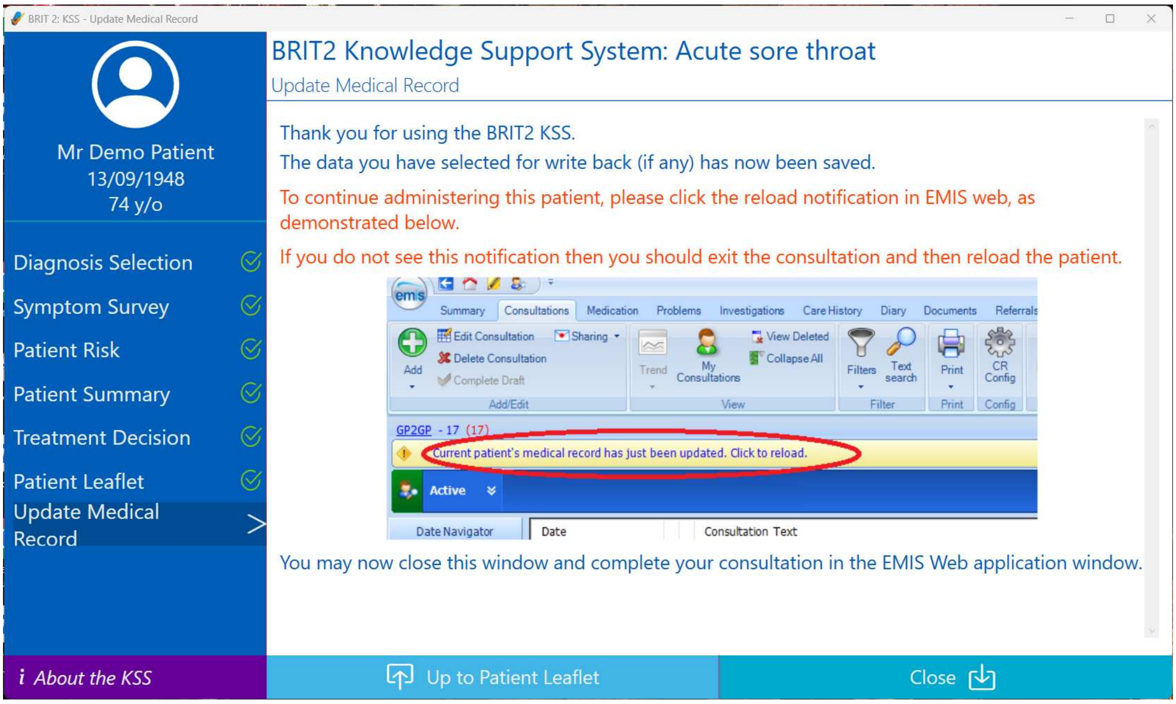
BRIT 2: KSS - Update Medical Record
BRIT2 Knowledge Support System: Acute sore throat
Update Medical Record
Mr Demo Patient
13/09/1948
74 y/o
Diagnosis Selection
Symptom Survey
Patient Risk
Patient Summary
Treatment Decision
Patient Leaflet
Update Medical Record
Thank you for using the BRIT2 KSS.
The data you have selected for write back (if any) has now been saved.
To continue administering this patient, please click the reload notification in EMIS web, as demonstrated below.
If you do not see this notification then you should exit the consultation and then reload the patient.
Summary
Consultations
Medication
Problems
Investigations
Care History
Diary
Documents
Referrals
Edit Consultation
Sharing
Delete Consultation
Complete Draft
Add
Trend
My Consultations
View Deleted
Collapse All
Filters
Text search
Print
CR Config
Add/Edit
View
Filter
Print
Config
GP2GP - 17 (17)
Current patient's medical record has just been updated. Click to reload.
Active
Date Navigator
Date
Consultation Text
You may now close this window and complete your consultation in the EMIS Web application window.
About the KSS
Up to Patient Leaflet
Close

Click here to print the patient information leaflet

Demo Patient
**Consultation:** 14/12/2022 **with:** Dr Tempest
**Suspected Diagnosis:** Pain in throat

## Explaining your treatment

**Treatment:** You should take pain relief and drink plenty of fluids. Ask your pharmacy for pain relief options. Use the back-up antibiotic if your symptoms do not improve within 3-5 days (currently 3 days)

**Explanation:** Most Pain in throat are self-limiting and get better on their own in 7 days. Return in 1 week if not better

Your risks have been compared to data from thousands of other patients like you to calculate the following:

## Risk of severe complications

Your risk of severe complications due to this infection is MODERATE : Out of 100 people who had similar health to you we would expect fewer than 29 to have serious complications

Your personal risk factors identified by this analysis are: Age, High BMI, Other medical conditions

Very low Increased

## Risk of antibiotic failure

Your risk of antibiotics failing this time is LOW. Around 10 per 100 patients like you will return to their doctor, for further treatment within a month.

Very low Increased

## Risk of carrying antibiotic resistant bacteria

You have used 1 courses of antibiotics in the last year. There is a LOW risk that you may carry antibiotic resistant bacteria.

Very low Increased

## Why was this treatment decision made?

Your health-care provider considers that an antibiotic may not be right for you at this moment. Your care provider has prescribed a back-up antibiotic to be used if you do not feel any better in 7 days. Return in 1 week if not better

Your care provider has considered the benefits of antibiotics as well your risk factors and the severity of this condition to come to this decision with you.

| Type of risk | What does this risk mean to me |
|---|---|
| **Complications from your infection (hospitalisation)** | **Explanation:** Most common infections are self-limiting and will clear up on their own without medication, but some people may have a higher risk of getting 'complications' (a worse infection). This 'complications' risk score helps your doctor identify people who might have a higher risk |
| **Risk of resistant bacterial infection** | **Explanation:** Good bacteria helps to protect the body from infection, but antibiotics can kill all bacteria good and bad. Without good bacteria to help fight infection, antibiotic resistant bacteria can grow and take over. When a bacteria becomes "resistant" to an antibiotic, that antibiotic can no longer be used to fight that infection. The more antibiotics you take, the higher the risk of developing resistant bacteria. |
| **Risk of antibiotic failing** | **Explanation:** Antibiotics are effective drugs and long as they are used correctly. They do not work on viral illnesses and could cause you to have more resistant bacteria and side effects. Research has shown that some people return to the GP within a month because the antibiotic treatment has not worked. |
| **Side effects and severe drug reaction** | **Explanation:** Antibiotics are not a 'risk free' treatment and they can cause a lot of unpleasant side effect including: sickness/ diarrhoea, rash, thrush, stomach pains, and being sick if you drink alcohol. They can also cause reactions when used with other medication you might be taking |

**Don't take antibiotics unless your doctor says you need them**

## What if my symptoms get worse?

### If you develop any of the warning signs below, call 999 (or visit your nearest A&E)

- Difficulty swallowing or breathing
- Drooling – this can be a sign of not being able to swallow
- Making a high-pitched sound as you breathe (called stridor)
- Have severe symptoms and are getting worse quickly

This Patient information leaflet has been generated by the BRIT2 project at the University of Manchester. If you have any questions, speak with your GP.
