# Supplementary material for: Knowledge support for optimising antibiotic prescribing for common infections in general practices: evaluation of the effectiveness of periodic feedback, decision support during consultations and peer comparisons in a cluster randomised trial (BRIT2) – study protocol
Source: BMJ Open. 2023 Aug 22;13(8):e076296. doi: 10.1136/bmjopen-2023-076296 (PMC10445367; doi:10.1136/bmjopen-2023-076296)
Supplement: Supplementary data [file bmjopen-2023-076296supp003.pdf]

MANCHESTER 1824
The University of Manchester

Building Resources to Improve Treatments (BRIT2)

A study of interventions to improve quality of care and optimisation of antibiotic prescribing using actionable information from advanced analytics.

Information Sheet: GM CARE RECORD

V1.0 12.10.2022

Your practice is being invited to take part in this study because your practice data forms part of the GM Integrated Care Record. Anonymised data from primary care is a valuable source for research, and has been a useful resource in understanding patient pathways in the COVID-19 pandemic. The attached Data Sharing Agreement (DSA) provided by Health Innovation Manchester asks practices to continue to provide anonymised data for research purposes to the BRIT2 research project at the University of Manchester.

The BRIT2 project

GP practices in the North West of England have been identified as high prescribers of antibiotics. Reducing or optimising prescribing will help to address issues of antimicrobial resistance (AMR) but better tools are needed to support GP practices to do this.

Researchers at the University of Manchester have developed a series of dashboards that uses data from GP practices to help understand where to focus efforts to optimise prescribing. The data the researchers get access to is anonymised at source, and remains in the datacente (authorised access provided to researchers). However, the GP practices get to see an identifiable version which allows a drill down to individual patients if necessary. For example, the dashboards can identify a number of patients who were inappropriately prescribed. The practice can look in more detail at who these patients were and understand where the issues might lie.

https://vimeo.com/764472108 (Test version, does not include enhanced dashboards)

The BRIT research team have also co-designed (though GP workshops) and developed a knowledge support system (KSS). The KSS supports the search and retrieval of context-specific knowledge from the Electronic Health Record system, to facilitate decision making at the point of care for common infections where an antibiotic may be considered as therapy. Characteristics of individual patients are also incorporated into risk analyses to generate patient specific risk assessments and are presented to prescribers for consideration. Appropriate NICE guidelines are provided for reference. Personalised patient leaflets can be generated to support patient understanding of prescribing decisions.

The University of Manchester

The KSS is a software package that has been built to integrate with the electronic health record system provided by EMISHealth. EMIS are the leading GP clinical IT system provider in the North West of England where the BRIT2 study is taking place. The KSS has been fully tested and validated by EMISHealth before being used in this study.

https://vimeo.com/766587482 (example, not final version)

Taking part in the BRIT2 research project.

We are offering the practice level dashboards to any practice who would like to see them – there is no obligation to take part in the study. Practices can use the dashboards as long as the study runs.

For the research study, We are looking for practices who would support the trial of the KSS and individual prescriber dashboards in a randomised controlled trial. A nominated individual in the practice will need to be a point of contact for the research team.

Randomisation will be to one of two groups, either A+B (Practice level and individual dashboards or A+B+C (includes the KSS)

All practices and individuals will receive a reminder once a quarter whilst the study is ongoing to remind them to view the dashboards. Training videos and support from the research team will be provided in the use of the KSS and navigating the dashboards.

Researchers will be able to see when dashboards and the KSS have been activated. This is an observational function of the study to allow researchers to report back to the funder if these systems are used, and the effectiveness of the systems. It does not allow researchers to see how the user is interacting with the dashboard or the KSS, only that they have opened it.

How long will the trial last?

The full trial is expected to last for 12 months (Dashboard) with use of the KSS for 9 months from July23 to March 24 to span the flu season.

Is there any payment to take part?

There is a payment of £250 to each practice who takes part in the trial, to cover associated research costs (time to read and sign documentation etc)

There is an additional payment of up to £1611 to each practice randomised to the KSS arm to cover time taken to use the KSS in consultation with patients.

What happens next?

If you agree to be part of this study, you will be asked to sign the attached:

1. Data Sharing agreement – this authorises use of anonymised data so that the practice can have their enhanced dashboards created.
2. Organisational Information Document: - this is a contract between the University of Manchester and the practice which allows the University to pay the research costs detailed above

Once documents have been received practices will need to request access to dashboards (the process for this will be provided)

Practices will then be randomised into the 2 arms of the study and will be informed of which arm they are in.

Those practices randomised into the KSS arm will be contacted by the IT service provider for their practice to allow installation of the KSS onto the practice systems. This is a tested and validated process that will not affect practice systems.

Anonymised data in the Trustworthy research environment will be accessed on a monthly basis by researchers as part of the research study.

Further information

This study is being organised and managed by the University of Manchester and funded by Public Health England (PHE), the National Institute of Health Research (NIHR) and Health Data Research UK (HDRUK). All patient-level data to be used for analysis will be anonymised and held in a secure Trustworthy Research Environments at Graphnet Health Ltd. The researchers will not disclose the names of practices or clinicians to people or organisations that have not been approved by the Data Controller. Any publications that are created following this study will use only anonymous information. No references to individual practices or prescribers will be made.

The study has been independently reviewed and given approval by the North East - Newcastle & North Tyneside 2 Research Ethics Committee Committee ref: 21/NE/0103 IRAS 290050

Thank you for reading the information sheet and for considering taking part in this research study.

The research behind the study: Recent research by the University of Manchester evaluated a variety of antibiotic (AB) prescribing measures for individual UK GPs, including overall,

MANCHESTER 1824
The University of Manchester

repeat or incidental AB prescribing, AB types prescribed and extent of risk-based prescribing (for infection-related hospital admissions). Results showed four-fold variability in overall prescribing rate, variability in clinician's propensity to repeat antibiotic prescribing and, variability in use of broad spectrum antibiotics. Almost all (96.4%) of clinicians exceeded the prescribing threshold for at least one prescribing measure. Those at higher risk of hospitalisation from an infection related condition, particularly frail elderly, those on multiple medications for comorbidities, are subject to the most variability in care. Targeting on risk-based prescribing would have the largest impact on optimising use of antibiotics in primary care. The research concluded that there is a need for a wider range of objectives to improve prescribing and quality of care, including varying engagement strategies with feedback tailored to each clinician, local context including bespoke recommendations that could be implemented and proactive support from colleagues and local organisations. Previously Public health England (PHE) have sought to impact AB prescribing using a variety of measures, including letters from the Chief Medical Officer to GPs in the top 20% of prescribers, which led to a 3.3-3.7% reduction in prescribing. In collaboration with GP's, AHPs prescribing advisors, patients, NICE and Public Health England (UK_HSA) , the BRIT2 research team have developed a series of dashboards that aim to help GPs adapt their prescribing practice to optimise the use of antibiotics, reduce inappropriate prescribing and use data to assist in evaluation and reporting antibiotic prescribing.

Relevant publications from this research group:

van der Zande, M.M., Dembinsky, M., Aresi, G. et al. General practitioners' accounts of negotiating antibiotic prescribing decisions with patients: a qualitative study on what influences antibiotic prescribing in low, medium and high prescribing practices. BMC Fam Pract 20, 172 (2019). https://doi.org/10.1186/s12875-019-1065-x

Victoria Palin, Anna Mölter, Miguel Belmonte, Darren M Ashcroft, Andrew White, William Welfare, Tjeerd van Staa, Antibiotic prescribing for common infections in UK general practice: variability and drivers, Journal of Antimicrobial Chemotherapy, Volume 74, Issue 8, August 2019, Pages 2440–2450, https://doi.org/10.1093/jac/dkz163

Magdalena Nowakowska, Tjeerd van Staa, Anna Mölter, Darren M Ashcroft, Jung Yin Tsang, Andrew White, William Welfare, Victoria Palin, Antibiotic choice in UK general practice: rates and drivers of potentially inappropriate antibiotic prescribing, Journal of Antimicrobial Chemotherapy, Volume 74, Issue 11, November 2019, Pages 3371–3378, https://doi.org/10.1093/jac/dkz345

Li, Y., Sperrin, M., Belmonte, M. et al. Do population-level risk prediction models that use routinely collected health data reliably predict individual risks? Sci Rep 9, 11222 (2019). https://doi.org/10.1038/s41598-019-47712-5

Yan Li, Anna Mölter, Andrew White, William Welfare, Victoria Palin, Miguel Belmonte, Darren M Ashcroft, Matthew Sperrin, Tjeerd Pieter van Staa. Relationship between prescribing of antibiotics and other medicines in primary care: a cross-sectional study. British Journal of General Practice 2019; 69 (678): e42-e51. DOI: 10.3399/bjgp18X700457

The University of Manchester

Anna Mölter, Miguel Belmonte, Victoria Palin, Chirag Mistry, Matthew Sperrin, Andrew White, William Welfare, Tjeerd Van Staa, Antibiotic prescribing patterns in general medical practices in England: Does area matter?, Health & Place, Volume 53, 2018, Pages 10-16, ISSN 1353-8292,https://doi.org/10.1016/j.healthplace.2018.07.004.

van Staa, T.P., Palin, V., Li, Y. et al. The effectiveness of frequent antibiotic use in reducing the risk of infection-related hospital admissions: results from two large population-based cohorts. BMC Med 18, 40 (2020). https://doi.org/10.1186/s12916-020-1504-5

Tjeerd Pieter van Staa, Victoria Palin, Benjamin Brown, William Welfare, Yan Li, Darren M Ashcroft, The Safety of Delayed Versus Immediate Antibiotic Prescribing for Upper Respiratory Tract Infections, Clinical Infectious Diseases, Volume 73, Issue 2, 15 July 2021, Pages e394–e401, https://doi.org/10.1093/cid/ciaa890

Victoria Palin, William Welfare, Darren M Ashcroft, Tjeerd Pieter van Staa, Shorter and Longer Courses of Antibiotics for Common Infections and the Association With Reductions of Infection-Related Complications Including Hospital Admissions, Clinical Infectious Diseases, Volume 73, Issue 10, 15 November 2021, Pages 1805–1812, https://doi.org/10.1093/cid/ciab159

Van Staa T, Li Y, Gold N, et al; Comparing antibiotic prescribing between clinicians in UK primary care: an analysis in a cohort study of eight different measures of antibiotic prescribing. BMJ Quality & Safety Published Online First: 03 March 2022. doi: 10.1136/bmjqs-2020-012108

Mistry, C., Palin, V., Li, Y. et al. Development and validation of a multivariable prediction model for infection-related complications in patients with common infections in UK primary care and the extent of risk-based prescribing of antibiotics. BMC Med 18, 118 (2020). https://doi.org/10.1186/s12916-020-01581-2

Ruth, Hurley, et al. "Clinician acceptability of an antibiotic prescribing knowledge support system for primary care: a mixed-method evaluation of features and context." (2022). https://doi.org/10.21203/rs.3.rs-1839693/v1
