# Supplementary material for: Knowledge support for optimising antibiotic prescribing for common infections in general practices: evaluation of the effectiveness of periodic feedback, decision support during consultations and peer comparisons in a cluster randomised trial (BRIT2) – study protocol
Source: BMJ Open. 2023 Aug 22;13(8):e076296. doi: 10.1136/bmjopen-2023-076296 (PMC10445367; doi:10.1136/bmjopen-2023-076296)
Supplement: Author's manuscript [file bmjopen-2023-076296.draft_revisions.pdf]

BMJ Open is committed to open peer review. As part of this commitment we make the peer review history of every article we publish publicly available.

When an article is published we post the peer reviewers' comments and the authors' responses online. We also post the versions of the paper that were used during peer review. These are the versions that the peer review comments apply to.

The versions of the paper that follow are the versions that were submitted during the peer review process. They are not the versions of record or the final published versions. They should not be cited or distributed as the published version of this manuscript.

BMJ Open is an open access journal and the full, final, typeset and author-corrected version of record of the manuscript is available on our site with no access controls, subscription charges or pay-per-view fees (http://bmjopen.bmj.com).

If you have any questions on BMJ Open's open peer review process please email info.bmjopen@bmj.com

BMJ Open

Knowledge support for optimising antibiotic prescribing for common infections in general practices: evaluation of the effectiveness of periodic feedback, decision support during consultations and peer comparisons in a cluster randomised trial (BRIT2)

Journal:	*BMJ Open*
Manuscript ID	bmjopen-2023-076296
Article Type:	Protocol
Date Submitted by the Author:	02-Jun-2023

Complete List of Authors:	van Staa, Tjeerd; The University of Manchester, Centre for Health Informatics<br>Sharma, Anita; NHS Oldham Clinical Commissioning Group, GP Oldham<br>Palin, Victoria ; The University of Manchester, Faculty of Biology, Medicine and Health<br>Fahmi, Ali; The University of Manchester, Centre for Health Informatics<br>Cant, Harriet; The University of Manchester, Centre for Health Informatics<br>Zhong, Xiaomin; The University of Manchester, Centre for Health Informatics<br>Jury, Francine; The University of Manchester, Centre for Health Informatics<br>Gold, Natalie; University of Oxford, Faculty of Philosophy<br>Welfare, William; UK Health Security Agency, Regions Directorate<br>Ashcroft, Darren; University of Manchester<br>Tsang, Jung Yin; University of Manchester, Centre for Primary Care and Health Services Research<br>Elliott, Rachel; The University of Manchester, Manchester Centre for Health Economics<br>Sutton, Christopher; University of Manchester, Division of Population Health, Health Services Research & Primary Care<br>Armitage, Chris; University of Manchester, Manchester Centre for Health Psychology<br>Couch, Philip; The University of Manchester, Centre for Health Informatics<br>Tempest, Edward; The University of Manchester, Centre for Health Informatics<br>Buchan, Iain; University of Liverpool, Public Health and Policy

Keywords:	Primary Care < Primary Health Care, Electronic Health Records, INFECTIOUS DISEASES, Randomized Controlled Trial

Knowledge support for optimising antibiotic prescribing for common infections in general practices: evaluation of the effectiveness of periodic feedback, decision support during consultations and peer comparisons in a cluster randomised trial (BRIT2)

Tjeerd Pieter van Staa[1], Anita Sharma[2], Victoria Palin[1,3], Ali Fahmi[1], Harriet Cant[1], Xiaomin Zhong[1], Francine Jury[1], Natalie Gold[4], William Welfare[5], Darren M Ashcroft[6,7], Jung Yin Tsang[1,6,7], Rachel A. Elliott[8], Chris Sutton[9], Christopher J. Armitage[7,10,11], Philip Couch[1], Edward Tempest[1], Iain Buchan[12]

[1]Centre for Health Informatics & Health Data Research UK North, Division of Informatics, Imaging and Data Science, School of Health Sciences, Faculty of Biology, Medicine and Health, University of Manchester, Manchester Academic Health Science Centre, Manchester, UK
[2]Chadderton South Health Centre, Eaves Lane, Chadderton, Oldham, OL9 8RG, UK
[3]Maternal and Foetal Research Centre, Division of Developmental Biology and Medicine, University of Manchester, St Marys Hospital, Oxford Road, Manchester, M13 9WL, UK
[4]Faculty of Philosophy, University of Oxford, Radcliffe Humanities, Woodstock Road, Oxford OX2 6GG
[5]Regions Directorate, UK Health Security Agency, 3 Piccadilly Place, London Road, Manchester, M1 3BN, UK
[6]NIHR School for Primary Care Research, Centre for Primary Care and Health Services Research, University of Manchester, UK
[7]NIHR Greater Manchester Patient Safety Research Collaboration (GMPSRC), University of Manchester, UK
[8]Manchester Centre for Health Economics, School of Health Sciences, Faculty of Biology, Medicine and Health, The University of Manchester, Manchester, UK
[9]Centre for Biostatistics, School of Health Sciences, Faculty of Biology, Medicine and Health, University of Manchester, Manchester Academic Health Science Centre, Manchester, UK
[10]Manchester Centre for Health Psychology, University of Manchester, Oxford Road, Manchester M13 9PL
[11]Manchester University NHS Foundation Trust, Manchester Academic Health Science Centre, Manchester, M13 9PL, UK
[12]Department of Public Health, Policy and Systems, Institute of Population Health, University of Liverpool, Liverpool, UK

BRIT2 Protocol 27_11_2022 Version 2.0 IRAS 290050
Research sponsor: University of Manchester (Faculty research governance team)

Corresponding author: Tjeerd van Staa, tjeerd.vanstaa@manchester.ac.uk; ORCID ID 0000-0001-9363-742X

For peer review only - http://bmjopen.bmj.com/site/about/guidelines.xhtml

Vaughan House, Centre for Health Informatics, Division of Informatics, Imaging and Data Science, School of Health Sciences, Faculty of Biology, Medicine and Health, The University of Manchester, Manchester Academic Health Science Centre, M13 9PL Manchester, United Kingdom

Key words: antibiotics, primary care, cluster randomised trial, electronic health records, patient information

Word count 3546 words

For peer review only

For peer review only - http://bmjopen.bmj.com/site/about/guidelines.xhtml

ABSTRACT

Introduction:

This project applies a Learning Healthcare System (LHS) approach to antibiotic prescribing for common infections in primary care. The approach involves iterations of data analysis, feedback to clinicians, and implementation of quality improvement activities by the clinicians. The main research question is, can a knowledge support system (KSS) intervention within a LHS implementation improve antibiotic prescribing without increasing the risk of complications?

Methods and analysis:

A pragmatic cluster randomised controlled trial will be conducted, with randomisation of at least 112 general practices in North-West England. General practices participating in the trial will be randomised to the following interventions: periodic practice-level and individual prescriber feedback using dashboards; or the same dashboards plus a KSS. Data from large databases of healthcare records are used to characterise heterogeneity in antibiotic uses, and to calculate risk scores for clinical outcomes and for the effectiveness of different treatment strategies. The results provide the baseline content for the dashboards and KSS. The KSS comprises a display within the electronic health record used during the consultation; the prescriber (GP or allied health professional) will answer standard questions about the patient's presentation and will then be presented with information (e.g. patient's risk of complications from the infection) to guide decision making. The KSS can generate information sheets for patients, conveyed by the clinicians during consultations. The primary outcome is the practice-level rate of antibiotic prescribing (per 1000 patients) with secondary safety outcomes. The data from practices participating in the trial and the dashboard infrastructure will be held within regional shared care record systems of the National Health Service in the UK.

Ethics and dissemination: Approved by NHS Ethics Committee IRAS 290050

Registration details: ISRCTN16230629

For peer review only

For peer review only - http://bmjopen.bmj.com/site/about/guidelines.xhtml

Strengths and limitations of this study

- This protocol describes a pragmatic cluster randomised controlled trial with randomisation of general practices to periodic practice-level and individual prescriber feedback using dashboards only compared to dashboards plus a knowledge support system (KSS) that can be activated during consultations.

- These interventions will be applied to antibiotic prescribing for common infections in primary care, an important area for clinical improvement given rising antimicrobial resistance.

- The design of the KSS was informed by two mixed-methods co-design workshops in which clinicians identified: key information to extract from care records (such as antibiotic prescribing history); recommended actions; personalised treatments; risk indicators; and content for patient information sheets.

- The primary research question is what is the effect on antibiotic prescribing of the KSS intervention within a learning health system implementation?

- A pilot phase will be initially conducted, with the recruitment target of 20 practices across two regions to examine feasibility and acceptability.

For peer review only

INTRODUCTION

Translating research findings into routine clinical practice is a major challenge. The Learning Healthcare System (LHS) approach has been proposed to better integrate research into clinical practice.[1] It involves iterations of data analysis (data to knowledge), feedback to clinicians (knowledge to performance) and implementation of quality improvement activities by clinicians (performance to data).[2,3] In the LHS, feedback can either be provided periodically through e.g. on-line dashboards or during consultations with patients using a Knowledge Support System (KSS). We use the term KSS as distinct from a Clinical Decision Support System because the information provided is about the condition, common complications and treatment options in general rather than specific decisions. Activation of KSS linked to electronic health records (EHRs) has been proposed for augmenting clinicians' knowledge during consultations with patients.[4] An icon on the clinicians' computer screen provides access to information on 'patients like mine' (e.g., the risk of developing clinical complications in similar patients), recommendations (e.g., best not prescribe amoxicillin given this patient's frequent prior use) and a patient information sheet tailored to their condition and treatment. The information on similar patients could be drawn from several sources such as historic data on clinical outcomes from comparable patient groups.[4] Thus, the KSS provides contextual information where a Clinical Decision Support System would point to a desired decision.[5]

In this project, the LHS approach will be applied to antibiotic prescribing for common infections in primary care. Primary care accounts for 72% of antibiotic prescribing in England.[6] Overuse of antibiotics is a major public health concern as it increases antimicrobial resistance. The NHS 2019 Long Term plan pledged action to optimise antibiotic uses, reducing inappropriate prescribing. The LHS approach will also include personalised patient information sheets that the clinician can provide to patients during consultation after KSS activation. A recent Cochrane review found that people exposed to decision aids feel more knowledgeable, better informed, and clearer about their values. It also found improved knowledge and accurate risk perceptions when decision aids are used within the consultation.[7]

Research questions

The research question is whether a KSS intervention within a LHS implementation can improve antibiotic prescribing without increasing the risk of complications? Practices that have implemented the KSS will be compared to practices that have not (i.e., randomised cluster trial). The LHS approach will involve detailed data analysis followed by feedback to clinicians.

METHODS AND ANALYSIS

Prior development of study interventions

Periodic feedback

For peer review only - http://bmjopen.bmj.com/site/about/guidelines.xhtml

A previous project piloted and implemented the IT infrastructure for the Learning Healthcare System (i.e., data analytics, feedback to clinicians) on antibiotic prescribing care in UK primary care, This project (BRIT: Building Rapid Interventions to reduce AMR and over-prescribing of antibiotics) was part of the £20 million Department of Health & Social Care (DHSC) funded Connected Health Cities programme (https://www.connectedhealthcities.org/). This initial BRIT project developed practice-level feedback on antibiotic together with feedback on individual prescribers.[8] These dashboards comprise a variety of antibiotic prescribing measures that were developed with clinical stakeholders.[9] They currently provide information at practice level on the drivers of antibiotic prescribing (overall and by indication), analyses of prescribing of inappropriate types of antibiotics (deviating from guideline) and extent of risk-based prescribing. Importantly, the dashboards will allow clinicians to review data at different levels, including e.g. prescribing rates of a specific antibiotic to the individual patients prescribed that antibiotic, and comparison to other practices in the region.[9]

KSS during consultation

Our KSS intervention was developed based on the results of two mixed-methods co-design workshops to gauge the acceptability of a prototype.[10] Clinicians identified the following key requirements: ease and efficiency of use, integration of systems, patient-centeredness, personalisation, and training. The KSS needs to include extraction of pertinent information from the care record (such as antibiotic prescribing history), recommended actions, personalised treatment, risk indicators and electronic patient information leaflets. The anticipated acceptability and intention to use the KSS, was moderate to high. While time was identified as a cost/burden, this was outweighed if the KSS improved patient outcomes and increased prescribing confidence. All the main requested features were implemented in the KSS except personalised treatment recommendations because current treatment guidelines do not cover all of the frequently encountered clinical challenges, such as frequent repeat antibiotic prescribing shortly after the first one.

Study design, participating sites and overview of interventions

The study will be a pragmatic cluster randomised controlled trial with 1:1 randomisation of practices to:

(i) Periodic feedback to general practices using practice-level + individual prescriber feedback dashboards. Appendix 1 provides examples of different dashboards.

or

(ii) Practice-level + individual prescriber feedback dashboards + KSS during consultation with the clinician providing content (such as an information sheet) that is personalised to the patient.

The study sites will include general practices located in the North-West of England that provide patient-level data to a regional shared care record system (https://www.graphnethealth.com), which is deployed in Greater Manchester, Wirral and Cheshire / Merseyside regions, together covering a 5.4 million general population.

For peer review only - http://bmjopen.bmj.com/site/about/guidelines.xhtml

The shared care record collates data from various NHS organisations to support direct patient care, service planning and research. Researchers access anonymised patient-level data through a secure data analytic environment linked to the shared care records.

Details of study interventions

Content of KSS for clinicians

Figure 1 shows the architecture of the KSS including data extracted from the patient's EHR, ability for clinicians to enter details on infection severity, estimation and presentation of risk scores, generation of personalised patient leaflet and write-back of codes to the EHR. The KSS will be available for all clinicians in the practices randomised into the KSS arm of the study. Eight screens are accessible within the KSS. Figure 2 shows Screen 1 of the KSS after activation (and Appendix 2 shows other examples of KSS screenshots). This provides for the selection of the type of common infection and indicators of problem significance and duration. Screen 2 is the symptom survey which provides a survey with list of symptoms to capture infection severity. An example is the Feverpain score for acute sore throat, including questions such as fever in past 24 hours, presence of cough or coryza and physical exam findings.[11] Screen 3 (patient summary) includes an extraction of relevant information as recorded in the patient's EHR. This includes the data on recent antibiotic prescribing (dates and types) and information on presence of allergies. This screen is populated automatically after KSS activation. Screen 4 (patient risks) gives personalised information including risks of:

1) Developing infection-related complications that lead to hospital admission,
2) Resistance based on number of prior antibiotics prescribed to the patient in the previous 12 months,
3) Antibiotic failure indicating probability may be prescribed another antibiotic within 30 days,
4) Severe adverse outcomes such as hospital admission for renal failure.

These personalised risk scores are based on risk prediction models including characteristics such as age, gender, clinical and medication risk factors, ethnicity and socioeconomic status. These risk prediction models were developed and tested in the OpenSAFELY platform (https://opensafely.org) or the Clinical Practice Research Datalink GOLD and Aurum (https://cprd.com/).[12,13] Risks are calculated using these algorithms which are combined with the patient's specific characteristics as recorded in the EHR. The clinician is given links to the relevant NICE guideline for the infection in Screen 5 (treatment guidelines). The clinician can then consider these risk scores alongside other information when considering whether to prescribe an antibiotic, line up a back-up antibiotic, or discuss with the patient alternative care pathways without antibiotics (treatment decision is recorded in Screen 6). Once the shared decision-making process is complete, the clinician may provide an information sheet for the patient, particularly where an antibiotic is not being prescribed to advise the patient of the decision and provide the personalised risk scores (Screen 7 with patient leaflet). Screen 8 allows the clinician to initiate a write-back function which will write back to the care record the diagnosis, symptoms and treatment as SNOMED codes. The KSS has been developed for use with EMIS

For peer review only - http://bmjopen.bmj.com/site/about/guidelines.xhtml

which is the commonest primary care record system in England.[14] Patient records remain confidential, and all data flows happen within the NHS.

KSS content for patients

We developed personalised patient information sheets that show tailored risk/benefit information based on patient-specific estimates of e.g., the risk of being admitted to hospital for infection-related complications. These information sheets are populated by the results of the KSS activation. The clinician can provide these to patients after consultation, in addition to any existing generic patient information sheets (TARGET) which also include information on illness duration, self-care advice, prevention advice and advice on when to re-consult [https://www.rcgp.org.uk/clinical-and-research/resources/toolkits/amr/target-antibiotics-toolkit/leaflets-to-share-with-patients.aspx]. Our advisory group including members of the public were involved in discussing and reviewing draft information sheets.

Sample size

The KSS arm will be limited to a maximum of 62 practices as this arm involves time and effort of clinicians during consultation. Sample size calculation has been computed based on the primary outcome of practice rate of antibiotic prescribing (per 1000 patients). In CPRD, the mean practice list size was 7981 and the overall rate of the number of antibiotic prescriptions (for any indication) was 598 per 1000 patients per year (standard deviation 155). Assuming a reduction of 10% in the low-intensity KSS arm, its antibiotic prescribing rate will be around 538 per 1000 patients per year. Assuming an unchanged SD of 155 and practice attrition rate of 5%, randomising 62 practices to each of the two arms will provide 90% power to detect a 10% (54 per 1000 patients per year) between-arm difference in the overall rate of antibiotic prescribing (two-sided α=0.05), assuming a correlation of 0.82 between baseline and outcome antibiotic prescribing rate and analysing using ANCOVA with baseline prescribing rate as covariate and 5% drop-out rate. For an 80% power, 47 practices are needed in each arm. Sample size calculations were performed in PASS 2019 software. Our primary target is 112 practices although we will recruit up to 124 practices, given the limitation on numbers in the KSS arm. However, determination of the total sample size of practices will depend on practice consent to take part in the KSS evaluation and passing an assessment of IT system capability for the KSS installation.

Study outcomes

The unit of analysis will be general practice. The primary clinical outcome will be the overall rate of antibiotic prescribing. One secondary outcome, relating to safety, will be infection-related complications as recorded in the primary care record, including pneumonia and lower respiratory tract infections, peritonsillar abscess, mastoiditis, intracranial abscess, empyema, scarlet fever, pyelonephritis, septic arthritis, osteomyelitis, meningitis, toxic shock syndrome and septicaemia, and Lemierre syndrome (as defined by Gulliford et al[15]). BRIT found that clinician-recorded infection-related complications provided comparable results as those

For peer review only - http://bmjopen.bmj.com/site/about/guidelines.xhtml

recorded in linked hospital admission data.[16] Another safety outcome will be hospital admissions for infection-related complications (linked hospital data are likely to be available within the shared care-record systems). The primary and safety-related secondary outcomes are equivalent to those used in a recent trial in UK primary care of a decision support system plus patient information sheets.[17] Another secondary outcome will be the level of risk-based prescribing (i.e., the proportion of antibiotic prescribing in patients with different risks) as based on the risk prediction scores as developed and validated in the BRIT project.[16]

A Statistical Analysis Plan (SAP), to be approved by the Steering Committee, will detail the planned analyses. Data analysts will have access to anonymised data from participating practices within a secure data analytic environment. Rates will be analysed using appropriate models with Poisson or normal error structures, weighted by practice size, and with adjustment for practice-level covariates including region (Greater Manchester, Wirral, and Merseyside/Cheshire), study quarter, period of randomisation and baseline antibiotic prescribing, socioeconomic status, case-mix of patients and ethnicity distribution in each practice (including Charlson comorbidity score[18]), consultation rates for common infections, coding propensity and characteristics of patients with common infections. The coding propensity will be the proportion of antibiotic prescriptions with recent clinical record for common infection. The characteristics of patients with common infections will include the averages in each practice for mean age, sex, and predictors for infection-related complication including clinical and medication risk factors[16]). Body mass index and smoking history will also be considered as potential predictors for infection-related complications; these will be discussed with the Study Steering Committee and will each be included in either the primary analysis, a sensitivity analysis or not included in the analysis. The primary analysis will be a complete case analysis (i.e., excluding practices who drop out of the trial), unless attrition is higher than expected (i.e., >10% overall or differential between-arm attrition of >10%).

Cost-effectiveness

NICE has produced guidance on the type of economic analysis needed for digital health technologies, depending on the level of financial risk to the NHS.[19,20] For a digital health technology like KSS that has the potential to be cost-saving, the economic analysis level could be defined as 'low financial commitment' requiring at least a cost-consequence analysis (CCA) and a budget impact analysis (BIA). We will estimate the economic impact of the KSS, from the perspective of the NHS and partner social care services The primary analysis will be a within-trial CCA and BIA where the outcomes are overall rate of antibiotic prescribing and level of risk-based prescribing. As we do not have access to infection-related hospital admissions, we need to use a proxy for this aspect of resource use. Therefore, we will also carry out an indicative BRIT2 algorithm-based economic analysis where we will use the data summarised above plus estimates of the expected rate of infection-related hospital admissions based on the validated BRIT2 risk algorithms, to provide indicative estimates of overall costs associated with KSS.

For peer review only - http://bmjopen.bmj.com/site/about/guidelines.xhtml

For peer review only

In the primary CCA, we will use the primary outcome, overall level of antibiotic prescribing, level of risk-based prescribing, infection-related hospital admissions and infection-related complications, to estimate economic impact of KSS.

Pilot phase

A pilot phase will be conducted, with the initial recruitment of 20 practices in two regions. In case of the trial not meeting this target, the study is to be terminated writing up feasibility and lessons learned without major statistical or pharmacoeconomic analyses. If the pilot target is met but the study recruits less than 94 practices, statistical or pharmacoeconomic analyses will be limited due to power.

Allocation and blinding

Allocation to interventions will use anonymised practice identifiers with randomisation stratified by region (Greater Manchester, Wirral and Merseyside/Cheshire) and baseline rate of antibiotic prescribing. The project manager will recruit practices and randomly allocate using https://www.sealedenvelope.com. The data analysts will not be blinded to intervention allocation. However, the SAP for the end-of-study analyses will be developed by the Lead Statistician from the Centre for Biostatistics who will not have access to study data and practice names throughout the study, and he will also perform the checking of statistical results to ensure that they are performed in accordance with the approved SAP.

Other study-related activities

Online community of practice and professional training

An online community of practice (OCoP), also known as a virtual community of practice, will also be implemented for interested practice prescribing advisors and clinicians to share knowledge through education on the challenges within their clinics and the opportunities to improve prescribing. The OCoP will provide a critical resource to professionals who want and need recommendations, pointers, tips and tricks, best practices, insights and innovations for optimising prescribing.[21,22] The purpose of this OCoP is to facilitate dialogue among experts and clinical stakeholders, present analysis results in order to collectively discuss the local challenges and opportunities for improving antibiotic prescribing that is relevant to the user.

The OCoP will focus on discussion of complex cases (e.g., patients who frequently but intermittently are prescribed antibiotics). It will also discuss the analytics of the local data and any actions that need to be implemented and evaluated locally – this will help researchers to understand the interpretation of the dashboard and provide feedback on the dashboard. The target users will be health professionals in the participating general practices as well as local healthcare (NHS) and public health organizations. The staff will include GPs, GP Research Leads, Practice Managers, Quality and Safety Pharmacists, Medicines

For peer review only - http://bmjopen.bmj.com/site/about/guidelines.xhtml

Optimizations Pharmacists and Public Health Consultants. We have already spoken to some of the community to develop this and when developing the OCoP we will build on this to provide a platform that will suit the way this community works. We need some champions to encourage widespread adoption of the resource. We will include some workshops using video conferencing to gain some of the input for the OCoP as well as provide training.

ETHICS AND DISSEMINATION

Ethics approval

Research Ethics Committee approval has been obtained (IRAS 290050). The study will be conducted in full conformance with all relevant legal requirements and the principles of the Declaration of Helsinki, Good Clinical Practice (GCP) and the UK Policy Framework for Health and Social Care Research 2017.

Publication policy

Dissemination of research outputs to participating clinical staff and policy and guideline developers will be an integral part of this project. In addition, we will promote dissemination to national policy makers, managers and clinical leaders, through project summaries and policy briefings. All study results will be reported in accordance to this study protocol and to the Consolidated Standards of Reporting Trials statement extended to cluster randomised trials.[23] The study sponsor and funder do not have any role in data collection, management, analysis, and interpretation of data; writing of the report; and the decision to submit the report for publication.

Patient and public involvement

Participatory workshops, where stakeholders are brought together to seek their opinions, extract knowledge and solve problems, were used to inform the design of the protocol. In addition, members of the public interested in health data research were invited through advertisement to the NIHR patient research ambassador network, to form an advisory group. This group has been meeting four times throughout the course of the research with a focus on patient risk communications. Members were also invited to rotate into the trial steering committee and research team meetings to add value and report back to the public advisory group.

Steering Committee

We have established an oversight committee, the Study Steering Committee, including senior representatives independent of the trial, comprising stakeholders in general practice, pharmacy, commissioning groups and patient representatives (6-monthly meetings) and statistician. Professor Janusz Jankowski, clinician and healthcare policy expert, chairs this committee. We follow NIHR guidance for programme steering committees [Research Governance Guidelines (nihr.ac.uk)].

Consent

For peer review only

General practices will need to agree with study participation and clinicians will need to activate the KSS within the care record system. Patient consent for use of the KSS will not be sought and is not required as approved by the Ethics Committee. The reason for this is that the KSS intervention is focused on clinicians – the KSS will provide clinicians with further information. It will be up the clinician to decide whether to access or not the KSS, prescribe an antibiotic or not. The KSS makes information easily accessible to the clinicians and will not provide treatment recommendations. Studies very similar to our proposal also did not seek informed consent from patients, but, like our study, required consent by participating clinicians.[24,25]

Device classification of the KSS

The UK regulator MHRA produced guidance on how to comply with the legal requirements for interventions including software. Based on a reasonable interpretation of the MHRA guidance, BRIT2 is not considered a device for the following reasons: the KSS will not apply 'automated reasoning' to the clinician's decision to prescribe an antibiotic or not. There will be no 'if then reasoning', no direct inferences can be drawn from the BRIT2 information provided. There will be no provision of a treatment threshold; it will be up to the clinician to decide whether a BRIT2 risk estimate of infection-related complications of e.g., 1% is relevant to the patient and whether this would require an antibiotic. BRIT2 will provide 'reference information to enable a clinician to make a clinician decision'.

Technical description of the Knowledge Support System

The initial version of the KSS has been written to target the EMIS Health's EMIS Web EHR system as EMIS Health provide EHR services to the majority of general practices in the trial region. This was a strategic decision aimed at controlling the complexity of the software and managing the delivery schedule while maximising the potential targets for the KSS randomisation. Future iterations of the KSS will be platform agnostic and present data in the presentation layer of the application in a consistent way regardless of EHR software running on site and early design decisions support this future requirement.

DCMB compliance

As the KSS is to be used to support the real-time direct care of patients, a rigorous clinical risk assessment was conducted to ensure compliance with the DCB0129 standards. As developed by NHS Digital, this standard "*provides a set of requirements suitably structured to promote and ensure the effective application of clinical risk management by those organisations that are responsible for the development and maintenance of Health IT Systems for use within the health and care environment*" [https://digital.nhs.uk/data-and-information/information-standards/information-standards-and-data-collections-including-extractions/publications-and-notifications/standards-and-collections/dcb0129-clinical-risk-management-its-application-in-the-manufacture-of-health-it-systems]. On evidence of compliance with the DCB0129

For peer review only - http://bmjopen.bmj.com/site/about/guidelines.xhtml

standards, approval was granted by EMIS to establish an API connection between KSS and the live patient record.

Acknowledgements The authors thank all the reviewers, clinicians and BRIT2 Patient Engagement Group for their assistance and support.

Contributors TPvS, ET, IB devised the project, and the main conceptual ideas. VP, NG, WW, CA, NG, WW, DA, RE, CS, CJA and PC were involved in the study design. TPvS, FJ, ET, CS were involved in drafting the manuscript. AS and JT provided general practice advice and AS and FJ are involved in consenting general practice. AF, HC, and XZ are responsible for analysing data. All authors reviewed the manuscript including the revisions, approved the final manuscript and accept accountability for the accuracy and integrity of the work.

Funding This work is supported by the National Institute for Health Research (HS&DR Project: NIHR130581) and concerns the cluster randomised trial to improve antibiotic prescribing in primary care. CJA is supported by the NIHR Manchester Biomedical Research Centre. DMA, CJA and RE are supported by the NIHR Greater Manchester Patient Safety Research Collaboration. The views expressed in this publication are those of the authors and not necessarily those of NIHR or the Department of Health and Social Care.

Competing interests None declared.

Patient and public involvement Patients and/or the public were involved in the design, or conduct, or reporting, or dissemination plans of this research. Refer to the Methods section for further details.

Patient consent for publication Not required.

Provenance and peer review Not commissioned; externally peer reviewed.

Supplemental material This content has been supplied by the author(s). It has not been vetted by BMJ Publishing Group Limited (BMJ) and may not have been peer-reviewed. Any opinions or recommendations discussed are solely those of the author(s) and are not endorsed by BMJ. BMJ disclaims all liability and responsibility arising from any reliance placed on the content. Where the content includes any translated material, BMJ does not warrant the accuracy and reliability of the translations (including but not limited to local regulations, clinical guidelines, terminology, drug names and drug dosages), and is not responsible for any error and/or omissions arising from translation and adaptation or otherwise.

Open access This is an open access article distributed in accordance with the Creative Commons Attribution Non Commercial (CC BY-NC 4.0) license, which permits others to distribute, remix, adapt, build upon this work non-commercially, and license their derivative works on different terms, provided the original work is properly cited, appropriate credit is given, any changes made indicated, and the use is non-commercial. See: http://creativecommons.org/licenses/by-nc/4.0/.

For peer review only - http://bmjopen.bmj.com/site/about/guidelines.xhtml

REFERENCES

1 Grossmann C, Sanders J, English RA, *et al.* Large Simple Trials and Knowledge Generation in a Learning Health System: Workshop Summary. Washington: : National Academies Press 2013. http://www.iom.edu/Reports/2013/Large-Simple-Trials-and-Knowledge-Generation-in-a-Learning-Health-System.aspx

2 Friedman CP, Rubin JC, Sullivan KJ. Toward an Information Infrastructure for Global Health Improvement. *Yearb Med Inform* 2017;**26**:16–23. doi:10.15265/IY-2017-004

3 Ainsworth J, Buchan I. Combining Health Data Uses to Ignite Health System Learning. *Methods Inf Med* 2015;**54**:479–87. doi:10.3414/ME15-01-0064

4 Longhurst CA, Harrington RA, Shah NH. A 'Green Button' For Using Aggregate Patient Data At The Point Of Care. *Health Aff* 2014;**33**:1229–35. doi:10.1377/hlthaff.2014.0099

5 Curtis CE, Al Bahar F, Marriott JF. The effectiveness of computerised decision support on antibiotic use in hospitals: A systematic review. *PLoS One* 2017;**12**:e0183062. doi:10.1371/journal.pone.0183062

6 English surveillance programme for antimicrobial utilisation and resistance (ESPAUR). 2022.

7 Stacey D, Légaré F, Lewis K, *et al.* Decision aids for people facing health treatment or screening decisions. *Cochrane Database Syst Rev* Published Online First: 12 April 2017. doi:10.1002/14651858.CD001431.pub5

8 Palin V, Tempest E, Mistry C, *et al.* Developing the infrastructure to support the optimisation of antibiotic prescribing using the learning healthcare system. *BMJ Heal Care Informatics* 2020;**27**. doi:10.1136/bmjhci-2020-100147

9 Palin V, Tempest E, Mistry C, *et al.* Developing the infrastructure to support the optimisation of antibiotic prescribing using the learning healthcare system to improve healthcare services in the provision of primary care in England. *BMJ Heal care informatics* 2020;**27**. doi:10.1136/bmjhci-2020-100147

10 Hurley R, Jury F, van Staa T, *et al.* Clinician acceptability of an antibiotic prescribing knowledge support system for primary care: a mixed-method evaluation of features and contexte. *BMC Heal Serv J* 2023;**23**:367. doi:10.1186/s12913-023-09239-4

11 Little P, Stuart B, Hobbs FDR, *et al.* Predictors of suppurative complications for acute sore throat in primary care: prospective clinical cohort study. *BMJ* 2013;**347**:f6867. doi:10.1136/bmj.f6867

12 Zhong X, Pate A, Yang Y-T, *et al.* The impact of COVID -19 on antibiotic prescribing in primary care in England: evaluation and risk prediction of appropriateness of type and repeat prescribing. *J Infect* Published Online First: May 2023. doi:10.1016/J.JINF.2023.05.010

13 Fahmi A, Palin V, Zhong X, *et al.* Evaluation of the impact of COVID-19 pandemic on hospital admission related to common infections. *submitted*

14 Kontopantelis E, Stevens RJ, Helms PJ, *et al.* Spatial distribution of clinical computer systems in

For peer review only - http://bmjopen.bmj.com/site/about/guidelines.xhtml

primary care in England in 2016 and implications for primary care electronic medical record databases: a cross-sectional population study. *BMJ Open* 2018;**8**:e020738. doi:10.1136/bmjopen-2017-020738

15 Gulliford MC, Juszczyk D, Prevost AT, *et al.* Electronically delivered interventions to reduce antibiotic prescribing for respiratory infections in primary care: Cluster RCT using electronic health records and cohort study. *Health Technol Assess (Rockv)* 2019;**23**:1–72. doi:10.3310/hta23110

16 Mistry C, Palin V, Li Y, *et al.* Development and validation of a multivariable prediction model for infection-related complications in patients with common infections in UK primary care and the extent of risk-based prescribing of antibiotics. *BMC Med* 2020;**18**:118. doi:10.1186/s12916-020-01581-2

17 Gulliford MC, Prevost AT, Charlton J, *et al.* Effectiveness and safety of electronically delivered prescribing feedback and decision support on antibiotic use for respiratory illness in primary care: REDUCE cluster randomised trial. *BMJ* 2019;**364**:l236. doi:10.1136/bmj.l236

18 Charlson M, Wells MT, Ullman R, *et al.* The Charlson comorbidity index can be used prospectively to identify patients who will incur high future costs. *PLoS One* 2014;**9**:e112479. doi:10.1371/journal.pone.0112479

19 Evidence standards framework for digital health technologies | Our programmes | What we do | About | NICE. https://www.nice.org.uk/about/what-we-do/our-programmes/evidence-standards-framework-for-digital-health-technologies (accessed 4 Mar2019).

20 Craig J. National Institute for Health and Care Excellenc e. Evidence Standards Framework for Digital Health Technologies Examples of Functional Classifications for Selected Digital Health Technologies. 2019.

21 Barnett S, Jones SC, Bennett S, *et al.* General practice training and virtual communities of practice - a review of the literature. *BMC Fam Pract* 2012;**13**:87. doi:10.1186/1471-2296-13-87

22 Bermejo-Caja CJ, Koatz D, Orrego C, *et al.* Acceptability and feasibility of a virtual community of practice to primary care professionals regarding patient empowerment: a qualitative pilot study. *BMC Health Serv Res* 2019;**19**:403. doi:10.1186/s12913-019-4185-z

23 Campbell MK, Piaggio G, Elbourne DR, *et al.* Consort 2010 statement: Extension to cluster randomised trials. *BMJ* 2012;**345**. doi:10.1136/bmj.e5661

24 Gulliford MC, Prevost AT, Charlton J, *et al.* Effectiveness and safety of electronically delivered prescribing feedback and decision support on antibiotic use for respiratory illness in primary care: REDUCE cluster randomised trial. *BMJ* 2019;**364**:l236. doi:10.1136/bmj.l236

25 Gulliford MC, van Staa TP, McDermott L, *et al.* Cluster randomized trials utilizing primary care electronic health records: Methodological issues in design, conduct, and analysis (eCRT Study). *Trials* 2014;**15**. doi:10.1186/1745-6215-15-220

For peer review only - http://bmjopen.bmj.com/site/about/guidelines.xhtml

Figure 1: Architecture of the KSS

Figure 2: Example of a KSS screen after activation within the care record system during consultation highlighting the patient's details (fictional) with the active record and the various screen available to the clinician

For peer review only

For peer review only - http://bmjopen.bmj.com/site/about/guidelines.xhtml

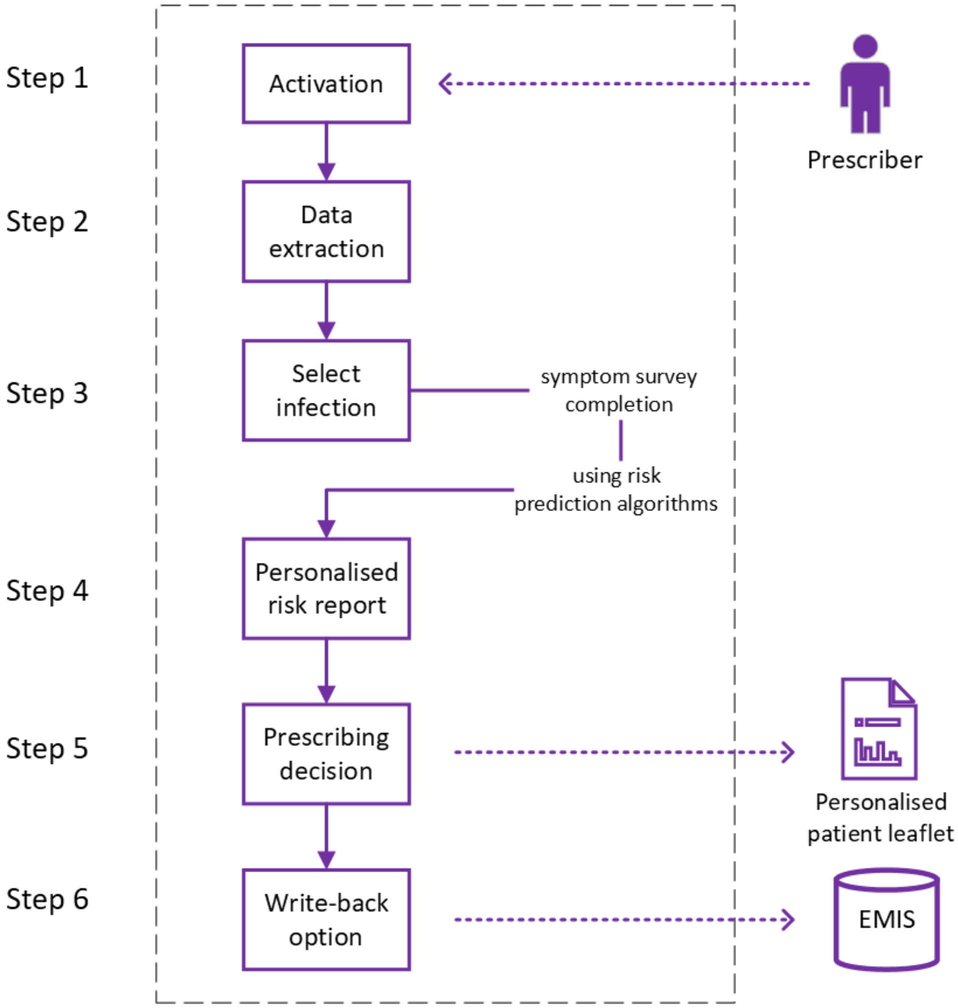

Architecture of the KSS

381x389mm (57 x 57 DPI)

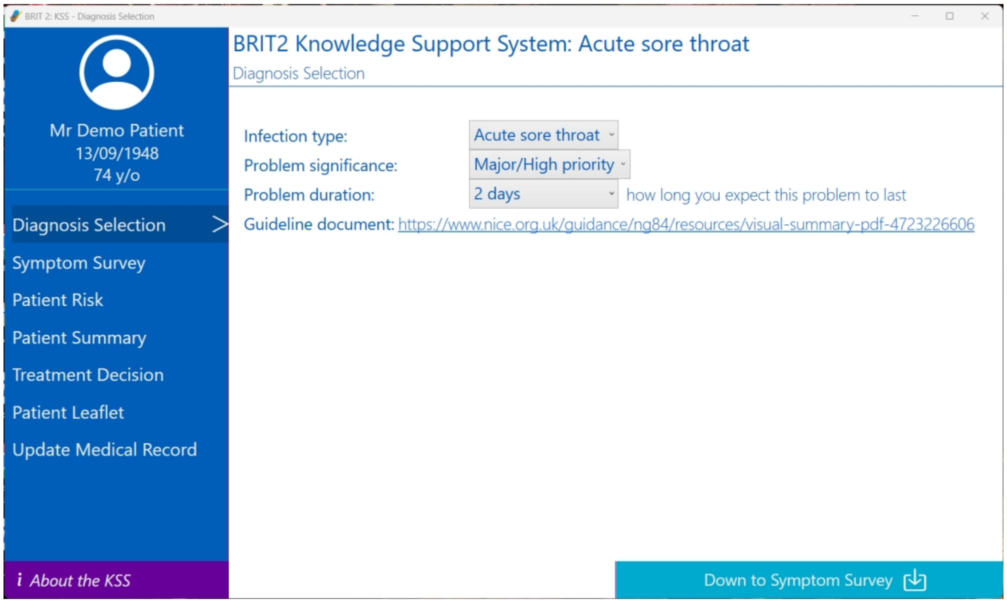

Example of a KSS screen after activation within the care record system during consultation highlighting the patient's details (fictional) with the active record and the various screen available to the clinician

904x536mm (47 x 47 DPI)

Appendix 1: Examples of dashboards as developed in BRIT project

Benchmarking

Data from large national data sources have been and will be used to get better understanding of the drivers for heterogeneity in care and for the effectiveness of different treatment strategies. The results will provide the baseline content for the dashboards.

A previous project piloted and implemented the IT infrastructure for the Learning Healthcare System (i.e., data analytics, feedback to clinicians) on antibiotic prescribing care in UK primary care, This project (BRIT: Building Rapid Interventions to reduce AMR and over-prescribing of antibiotics) was part of the £20 million Department of Health & Social Care (DHSC) funded Connected Health Cities programme (https://www.connectedhealthcities.org/). The data analytics activities in BRIT consisted of benchmarking current practice in primary care, evaluating the levels of suboptimal antibiotic prescribing and identifying opportunities for improvement. Large variability in antibiotic prescribing was observed between practices and within practices: Change points in prescribing did not reflect updates to national guidelines. Prescribing levels within practices were not consistent for different infectious conditions. BRIT also found high levels of prescribing of potentially inappropriate type of antibiotics which were highest for otitis externa (67.3%) and upper respiratory tract infection (38.7%). BRIT found that that over the last 15 years antibiotic prescribing in primary care was not risk-based: patients with very low risk of infection-related hospital admissions were as likely to receive an antibiotic as patients with higher risks. BRIT also evaluated the effectiveness of treating common infections with antibiotics. The findings also indicate that incidental use of antibiotics is effective in reducing infection-related hospital admissions while repeated courses of antibiotics may have limited benefit and be indicative of adverse outcomes. Of 5.1 million antibiotics prescribed in UK primary care, only 14.8% were given to patients without any antibiotic prescribing in the previous three years and 43.6% are for patients who already received 5+ antibiotic prescriptions in the previous three years. These BRIT findings indicate that optimal antibiotic prescribing in primary care is a complex interplay of a patient's symptoms, age and co-morbidity and previous history of antibiotic use. While incidental use of an antibiotic may reduce the risk of infection-related complications, it may also decrease the effectiveness of the antibiotic for future infections (possibly due to the development of resistance in the patient [1]). This highlights the importance for patient-specific communication, information and algorithms that recommend best course of action.

Recent research found considerable variability between GPs in the case mix of patients consulting and large variability in antibiotic prescribing habits. The majority of clinicians (> 95%) prescribed at least one antibiotic measure that was above the medians of their peers. It concluded that there is a need for a wider range of objectives (using a variety of measures without ranking of clinicians based on a single metric), varying engagement strategies with feedback tailored to each clinician, local context including bespoke

For peer review only

recommendations that could be implemented and proactive support from colleagues and local organisations [2].

The LHS approach is not just about analysis of data and feedback of results to clinicians. An important aspect is around understanding and changing behaviours which is a focus of behavioural sciences [3]. An example of this is the provision of social norm practice-level feedback (i.e., comparing a clinician to other peers). In a UK cluster randomised controlled trial (cRCT), this approach was found to substantially reduce antibiotic prescribing. Every GP in the feedback intervention group was sent a letter from England's Chief Medical Officer [4]. A US cRCT tested the effects of several behavioural interventions and found that peer comparisons resulted in lower rates of inappropriate antibiotic prescribing for acute respiratory tract infection. These comparisons consisted of emails sent to clinicians that compared their antibiotic prescribing rates with those with the lowest inappropriate prescribing rates [5].

The BRIT dashboard summarises information on antibiotic prescribing by infection type and where these prescriptions may deviate from the recommended guidelines, overall prescribing rates with comparision to peers, as well as how a practice may prescribe based on a pratients risk of an infection-related complication. On the hompage users have the option to clickthe lage icons or use the menu bar across the top to navigete to each dashbord.

The first group of visualisations (Supplementary Figure 1A) shows the general practices (GPs) inforation about their antibiotic prescribing by infectious conditions. The first part of the dashboard shows colourful notification boxes with the number of infection-related consultations in a given time period (yellow box), the number of antibiotic prescriptions issued for these consultations (blue box), the number of these prescriptions issed that deviated from the national recommended guidelines (red box) and a nudging notification box (in green) that suggests areas for improvement in their prescribing based on which condition saw more deviating prescriptions. Users reviewing the dashboard, such as prescribing advisor may decide to concentrate their efforts when auditing the practices antibiotic prescribing in a given month by delving deeper into the prescriptions issued for consultations were the system flagged the most inappropriate prescriptions (in this example upper respiratory tract infections; URTI). Next the user will see a box and whisker plot showing national prescribing rates by infection, i.e. for each practice the percentage of consultations that resulted in an antibiotic prescription by infection type (Supplementary Figure 1B). The users of the BRIT dashboard can also see where their practice sits (the black dots overlaid on this plot) compared to the national prescribing rates. For example, Supplementary Figure 1 shows the practice has a higher prescribing rate for breast-related infections compared to the national average but have a lover prescribing rate for ear-related infections (otitis externa and otitis media), highlighting which infections warrant further investigation in their practice. The practice can also see what prescriptions are being prescribed to each infectious condition as a percentage in a sunburst plot, where Supplementary figure 1C shows that when selecting lower respiratiory tract infections

For peer review only

(LRTI) over three quarters of the prescriptions their practice prescribed for this condition were fore amoxicillian, followed by doxycycline and clarithromycin as well as other antibiotic less commonly prescribed to these conditionsn in their practice in a given time period.

The practice can also see how their practice is performing in terms of prescribing appropriateness (Figure 1D). The analysis behind these plots assess wheather each antibioitc prescription in agreement (blue) or disagreement (red) with the recommended first- second- or alternative- medicine. For example this plot shows a hypothetical practice were 24% of the consultations for sinusitis resulted in an antibiotic prescription that may have been inappropriate because it deviated form the recommended guidelines. They can also see for each condition what antibiotic type was issued and flagged as potentially inappropriate (figure not shown). If they were to scroll down on the sape dashboard there are snapshots of the most recent antibiotic guidelines , enabiling a quick comparision of the antibiotics they have prescribed deviating from recommendations and refreshing what antibiotics are currently recommended appropriate for each condition. Again, this analysis highlights areas for review/improvement specific to each practice that may instigate discussion in a practice manegement meeting ensuring prescribers have refreshed their knowledge on the current prescribing guidelines for a particular infection.

A second topic of analysis is looking at how well a practice prescribed based on a patients predicted risk of a poor outcome (e.g., infection-related hospitalisation). The analysis is based on a validated risk prediction model using two national datasets . Supplementary Figure 2 shows the predicted risk (using the prediction model) for every patient presenting with a particular infection (in this case LRTI) in a given time period (6 months), separated into categories from very low predicted risk to very high predicted risk of complication. For each category the actual prescribing rate is caluculated as a percentage and displayed by the bule bars. Practices prescribing according to patients risk of complication would expect to see the height of the blue bars match the red line here, where very low risk patients received prescriptions on occasion, perhapse due to the severity of their symptom and the very high risk patients are receiving a prescription more often. However, this figure shows a flat line across all risk categories again suggesting prescribing for this infection is not well targeted to high risk patients and there is room for improvement. The dashboard also has a built in a risk prediction calculator, allowing users modify various patient characteristics and see which effects the predicted risk more, again adding a training element to the dashboard that can help to identify ways to further optimise their prescribing.

The final topic of analysis is prescribing benchmarking. The traffic-light visulisation (Supplementary figure 3A) shows STAR-PU adjusted prescriing rates for each practice. This is an age-sex adjusted prescribing rate allowing fair comparison between practices. The yellow dots show bottom 25% of practices with a relatively low prescribing rate, the red dots show the top 25% of high prescribing practices and the green is the middle 50% percent of practices with an average prescribing rate. The black dot then shows where the individual

For peer review only

practice sits compared to their peers. The bottom figure (Supplementary figure 3B) shows the practices prescribing rate over time. This is important compare how a practice is prescribing to their peerd but to also observe any rapid changes to their prescribing rates following an intervention; allowing rapid review and uptake of interventions that work and removal of those that do not. The practice can also use these sort of comparison plots to see seasonality trends as well as changes in prescribing due to an outbreak of a pandemic disease, such as COVID-19. Here we can see the prescribing rate in April 2018 and April 2019 for this demostrative practice was 31.5 units of antibiotics for every 1000 registered patients; however following the outbreak of COVID-19 the prescribing rate in April 2020 has reduced to 23.85 units of antibiotics prescribed per 1000 registered patients.

The data in the dashboard is regularly updated providing practices with frequent incited to their own prescribing and how changes they make can alter and optimise their prescribing. The content of the dahboard visualisations also evolving in an iterative manner based on feedback from users and key stakholders, creating a learning health care system that is evolving with the health care our patner practices provide.

For peer review only

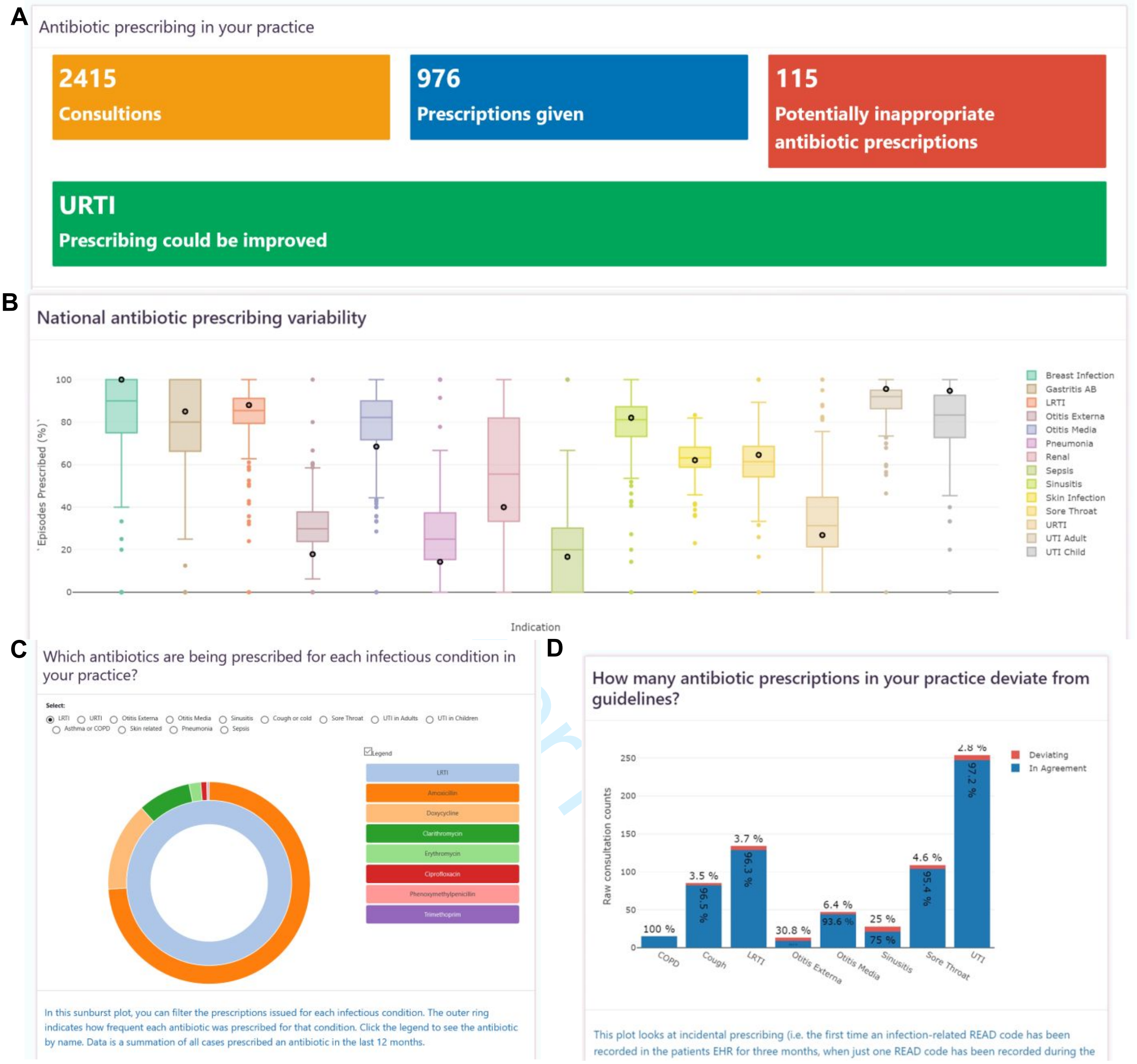

Supplementary Figure 1: Examples of dashboards providing periodic feedback on antibiotic prescribing of practices

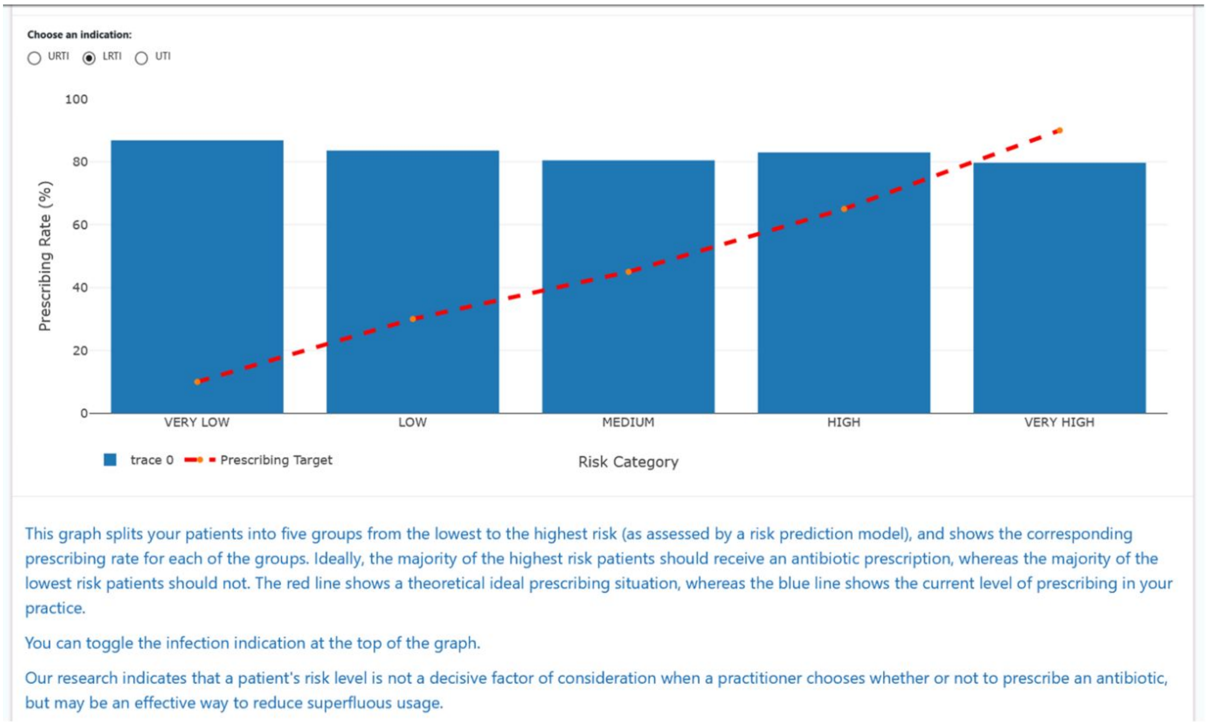

Supplementary Figure 2: dashboards of antibiotic prescribing by patient risk profile

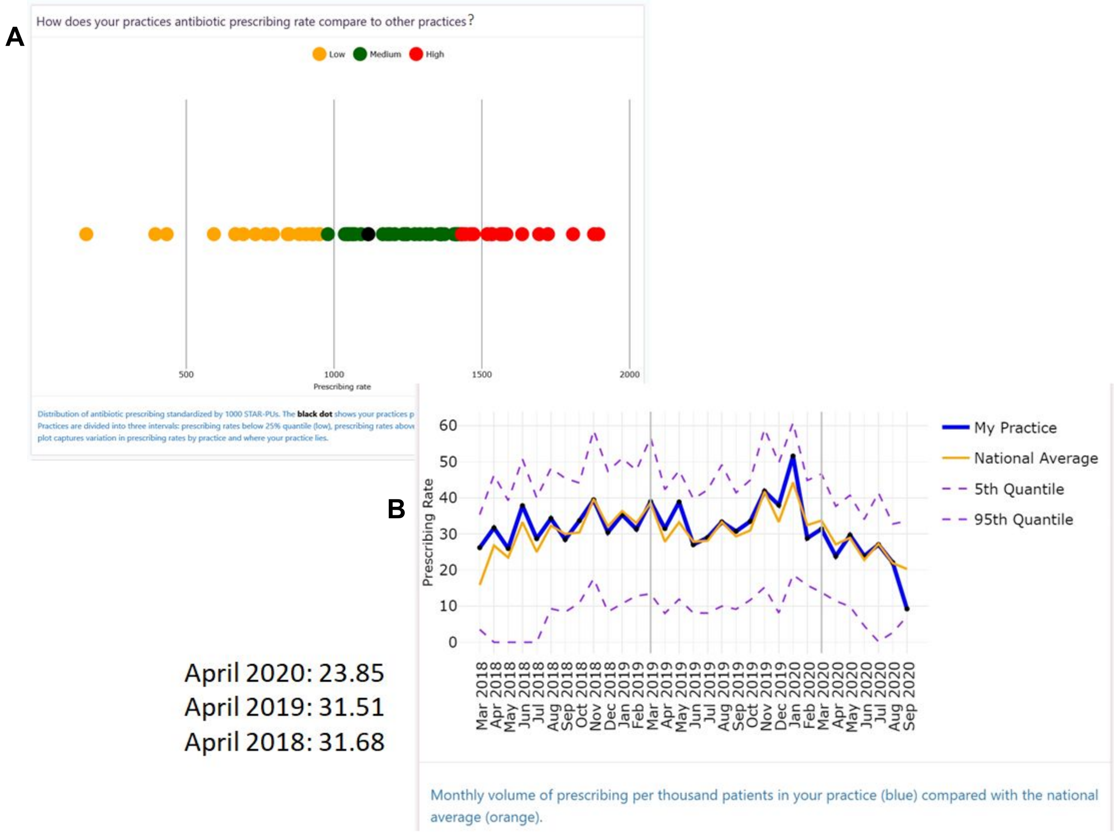

Supplementary Figure 3: Dashboards of practice-level antibiotic prescribing rates

References

1. Costelloe C, Metcalfe C, Lovering A, Mant D, Hay AD. Effect of antibiotic prescribing in primary care on antimicrobial resistance in individual patients: systematic review and meta-analysis. BMJ [Internet]. 2010 [cited 2018 Sep 15];340:c2096. Available from: http://www.ncbi.nlm.nih.gov/pubmed/20483949
2. Van Staa T, Li Y, Gold N, Chadborn T, Welfare W, Palin V, et al. Comparing antibiotic prescribing between clinicians in UK primary care: an analysis in a cohort study of eight different measures of antibiotic prescribing. BMJ Qual Saf [Internet]. BMJ Qual Saf; 2022 [cited 2022 Sep 6];bmjqs-2020-012108. Available from: https://pubmed.ncbi.nlm.nih.gov/35241573/
3. Improving people’s health: Applying behavioural and social sciences to improve population health and wellbeing in England [Internet]. 2018. Available from: www.facebook.com/PublicHealthEngland
4. Hallsworth M, Chadborn T, Sallis A, Sanders M, Berry D, Greaves F, et al. Provision of social norm feedback to high prescribers of antibiotics in general practice: a pragmatic national randomised controlled trial. Lancet [Internet]. 2016 [cited 2018 Sep 15];387:1743–52. Available from: http://www.ncbi.nlm.nih.gov/pubmed/26898856
5. Meeker D, Linder JA, Fox CR, Friedberg MW, Persell SD, Goldstein NJ, et al. Effect of behavioral interventions on inappropriate antibiotic prescribing among primary care practices a randomized clinical trial. JAMA - J Am Med Assoc. American Medical Association; 2016;315:562–70.

For peer review only

Appendix 2: Examples of KSS screens and personalised patient leaflet

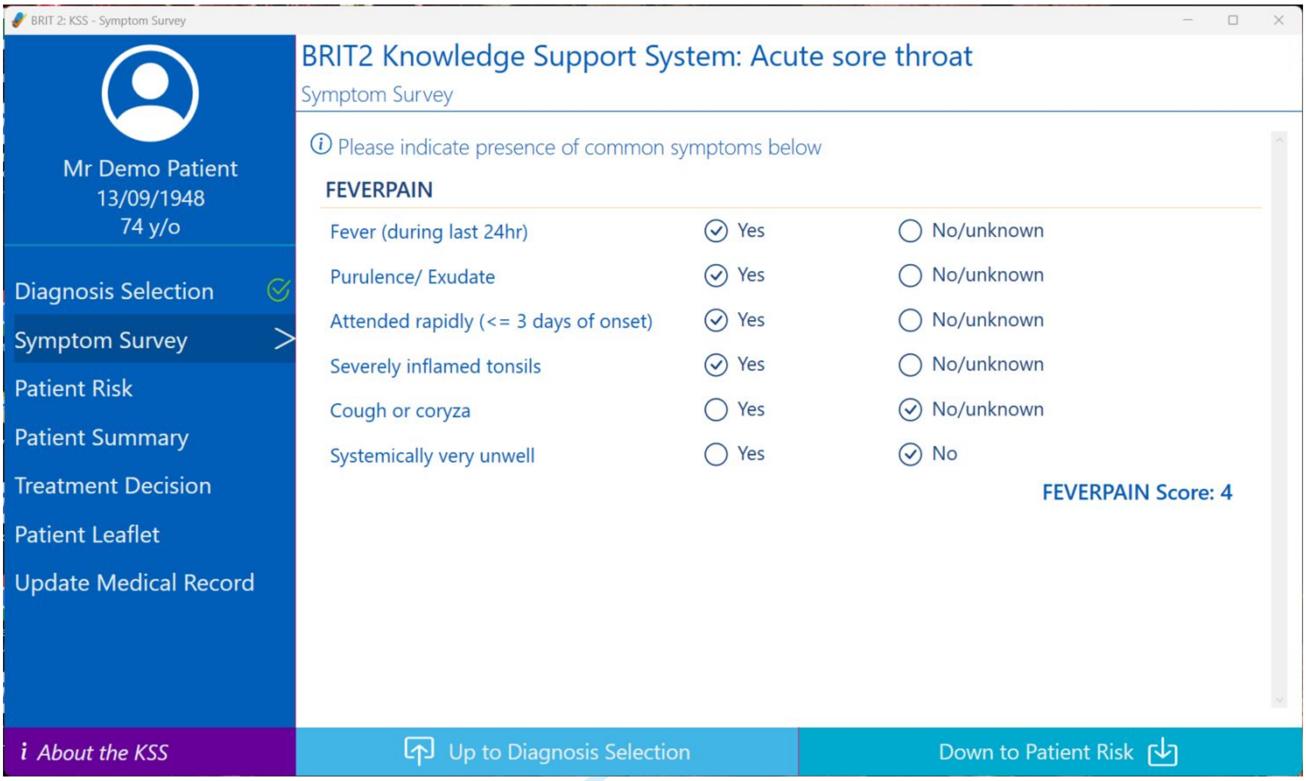

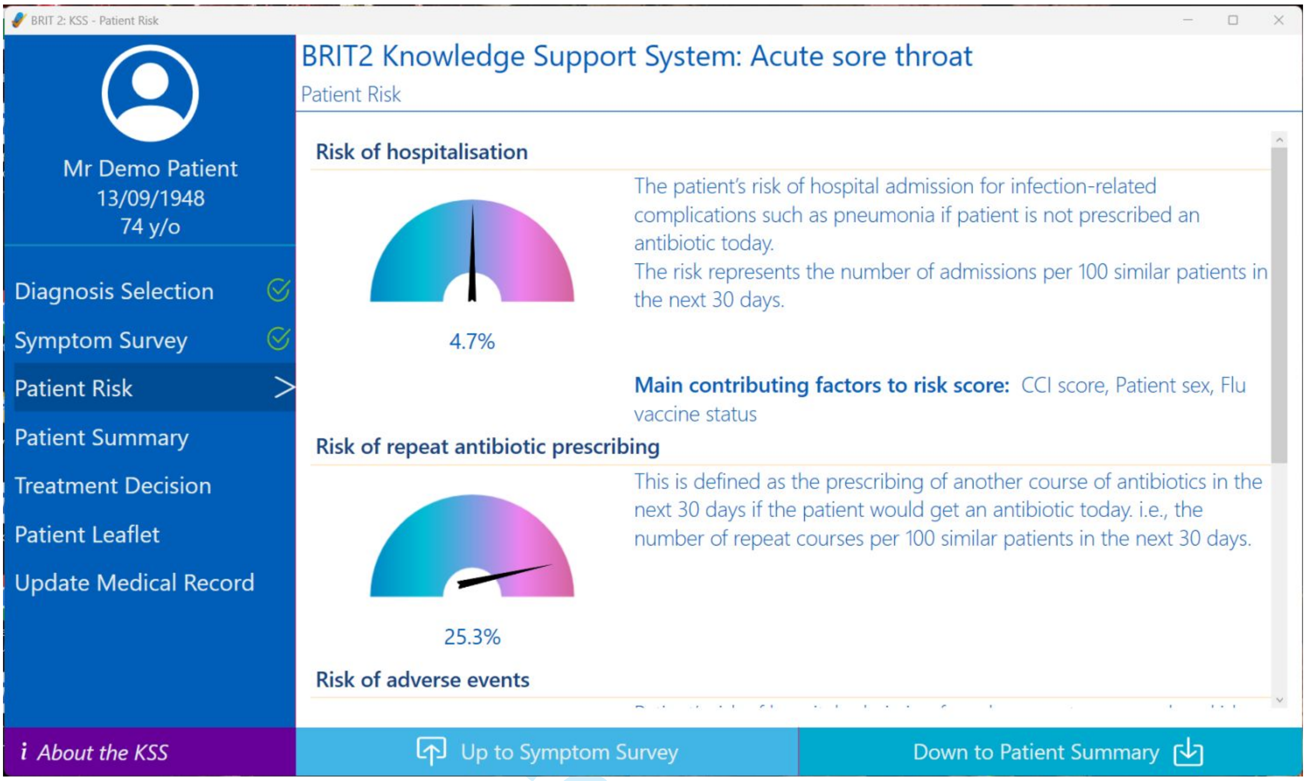
BRIT 2: KSS - Patient Risk
BRIT2 Knowledge Support System: Acute sore throat
Patient Risk
Mr Demo Patient
13/09/1948
74 y/o
Diagnosis Selection
Symptom Survey
Patient Risk
Patient Summary
Treatment Decision
Patient Leaflet
Update Medical Record
About the KSS
Risk of hospitalisation
4.7%
The patient's risk of hospital admission for infection-related complications such as pneumonia if patient is not prescribed an antibiotic today.
The risk represents the number of admissions per 100 similar patients in the next 30 days.
Main contributing factors to risk score: CCI score, Patient sex, Flu vaccine status
Risk of repeat antibiotic prescribing
25.3%
This is defined as the prescribing of another course of antibiotics in the next 30 days if the patient would get an antibiotic today. i.e., the number of repeat courses per 100 similar patients in the next 30 days.
Risk of adverse events
Up to Symptom Survey
Down to Patient Summary

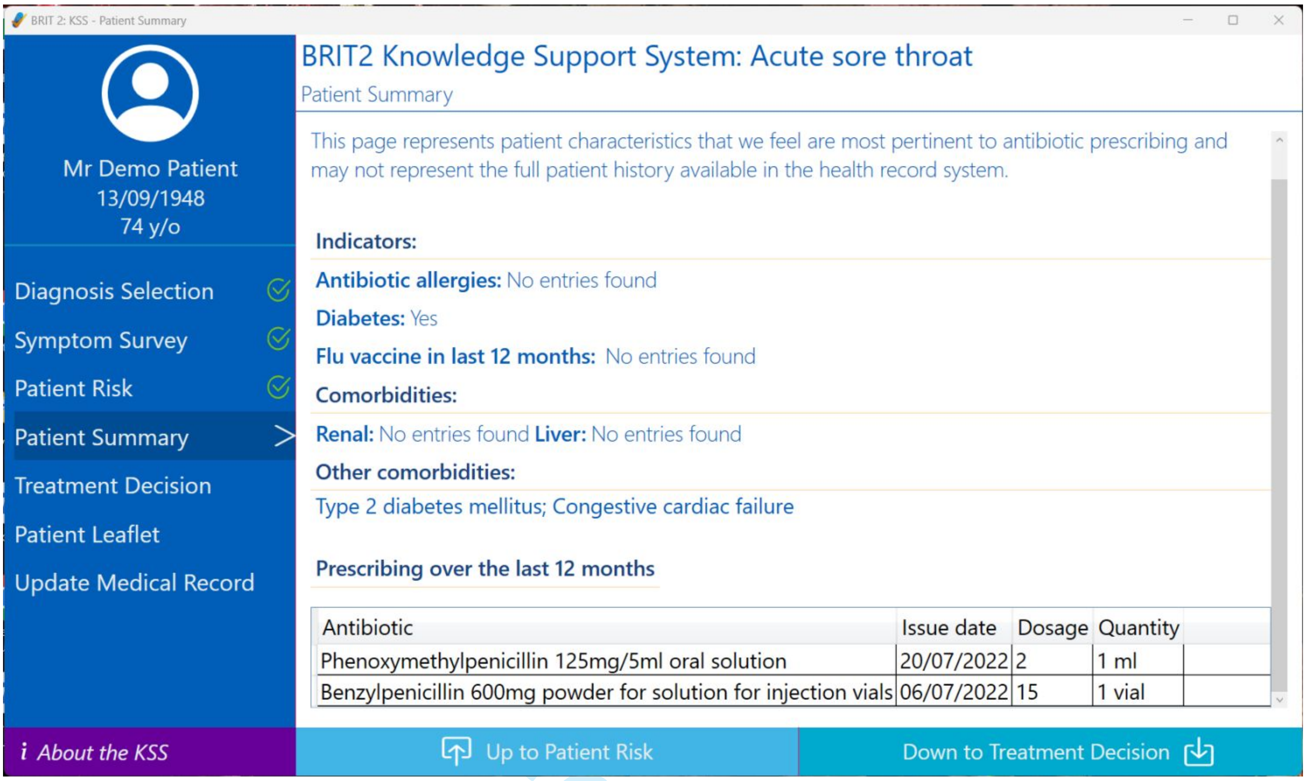
BRIT 2: KSS - Patient Summary
Mr Demo Patient
13/09/1948
74 y/o
Diagnosis Selection
Symptom Survey
Patient Risk
Patient Summary
Treatment Decision
Patient Leaflet
Update Medical Record
About the KSS
BRIT2 Knowledge Support System: Acute sore throat
Patient Summary
This page represents patient characteristics that we feel are most pertinent to antibiotic prescribing and may not represent the full patient history available in the health record system.
Indicators:
Antibiotic allergies: No entries found
Diabetes: Yes
Flu vaccine in last 12 months: No entries found
Comorbidities:
Renal: No entries found Liver: No entries found
Other comorbidities:
Type 2 diabetes mellitus; Congestive cardiac failure
Prescribing over the last 12 months
Antibiotic | Issue date | Dosage | Quantity
Phenoxymethylpenicillin 125mg/5ml oral solution | 20/07/2022 | 2 | 1 ml
Benzylpenicillin 600mg powder for solution for injection vials | 06/07/2022 | 15 | 1 vial
Up to Patient Risk
Down to Treatment Decision

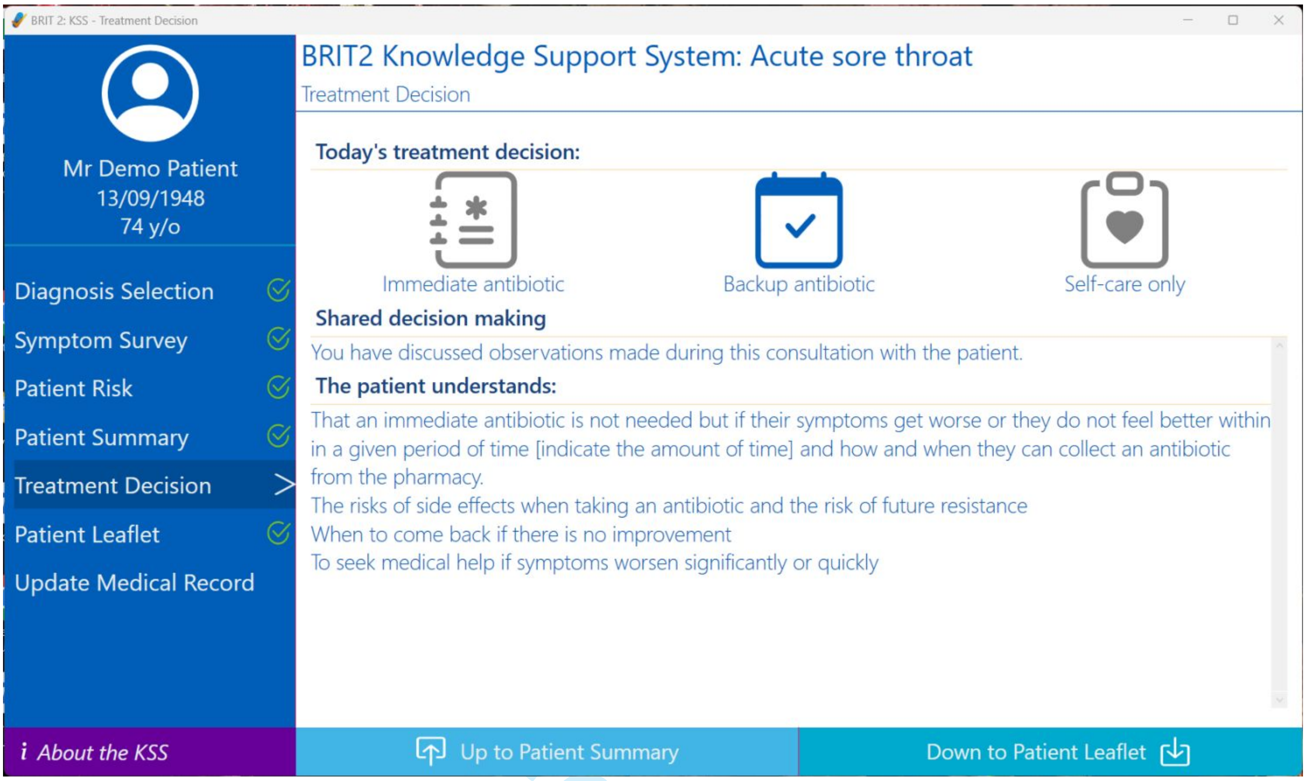
BRIT 2: KSS - Treatment Decision
BRIT2 Knowledge Support System: Acute sore throat
Treatment Decision
Mr Demo Patient
13/09/1948
74 y/o
Diagnosis Selection
Symptom Survey
Patient Risk
Patient Summary
Treatment Decision
Patient Leaflet
Update Medical Record
Today's treatment decision:
Immediate antibiotic
Backup antibiotic
Self-care only
Shared decision making
You have discussed observations made during this consultation with the patient.
The patient understands:
That an immediate antibiotic is not needed but if their symptoms get worse or they do not feel better within in a given period of time [indicate the amount of time] and how and when they can collect an antibiotic from the pharmacy.
The risks of side effects when taking an antibiotic and the risk of future resistance
When to come back if there is no improvement
To seek medical help if symptoms worsen significantly or quickly
i About the KSS
Up to Patient Summary
Down to Patient Leaflet

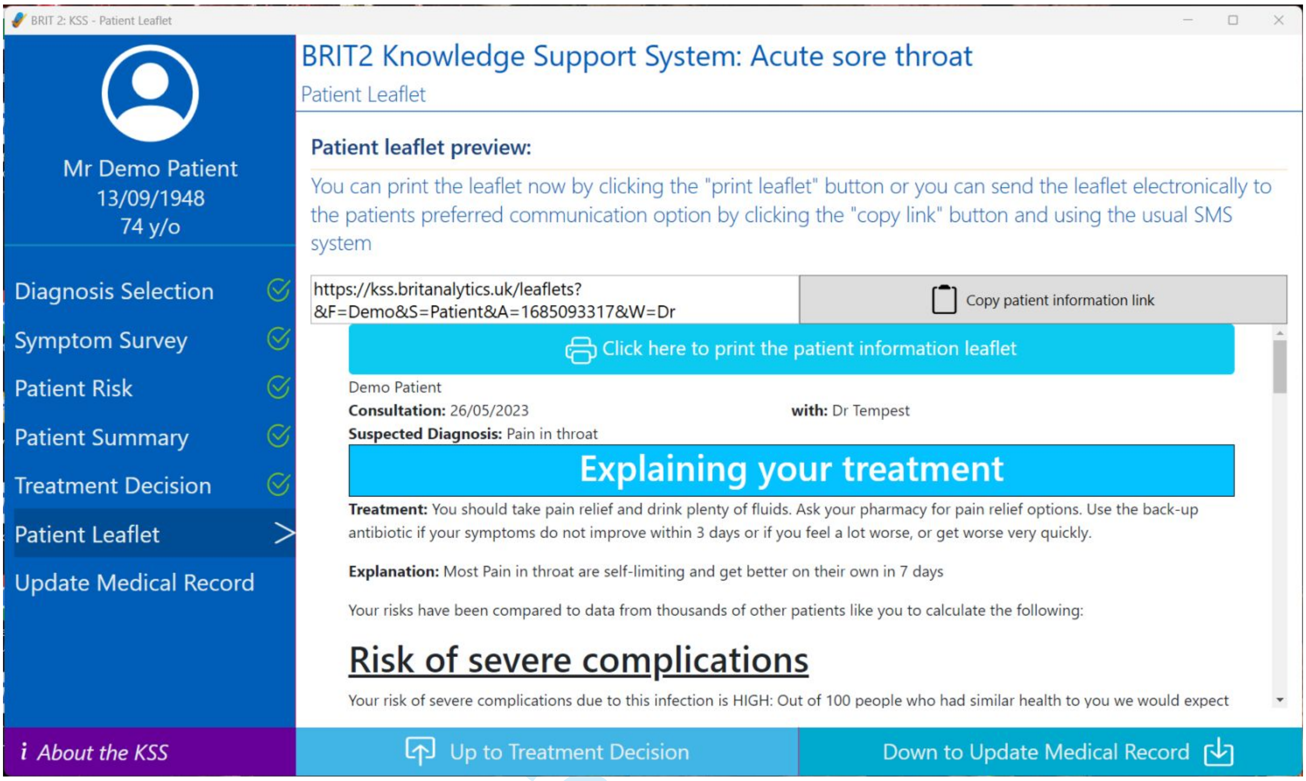
BRIT 2: KSS - Patient Leaflet
Mr Demo Patient
13/09/1948
74 y/o
Diagnosis Selection
Symptom Survey
Patient Risk
Patient Summary
Treatment Decision
Patient Leaflet
Update Medical Record
About the KSS
BRIT2 Knowledge Support System: Acute sore throat
Patient Leaflet
Patient leaflet preview:
You can print the leaflet now by clicking the "print leaflet" button or you can send the leaflet electronically to the patients preferred communication option by clicking the "copy link" button and using the usual SMS system
https://kss.britanalytics.uk/leaflets?&F=Demo&S=Patient&A=1685093317&W=Dr
Copy patient information link
Click here to print the patient information leaflet
Demo Patient
Consultation: 26/05/2023
with: Dr Tempest
Suspected Diagnosis: Pain in throat
Explaining your treatment
Treatment: You should take pain relief and drink plenty of fluids. Ask your pharmacy for pain relief options. Use the back-up antibiotic if your symptoms do not improve within 3 days or if you feel a lot worse, or get worse very quickly.
Explanation: Most Pain in throat are self-limiting and get better on their own in 7 days
Your risks have been compared to data from thousands of other patients like you to calculate the following:
Risk of severe complications
Your risk of severe complications due to this infection is HIGH: Out of 100 people who had similar health to you we would expect
Up to Treatment Decision
Down to Update Medical Record

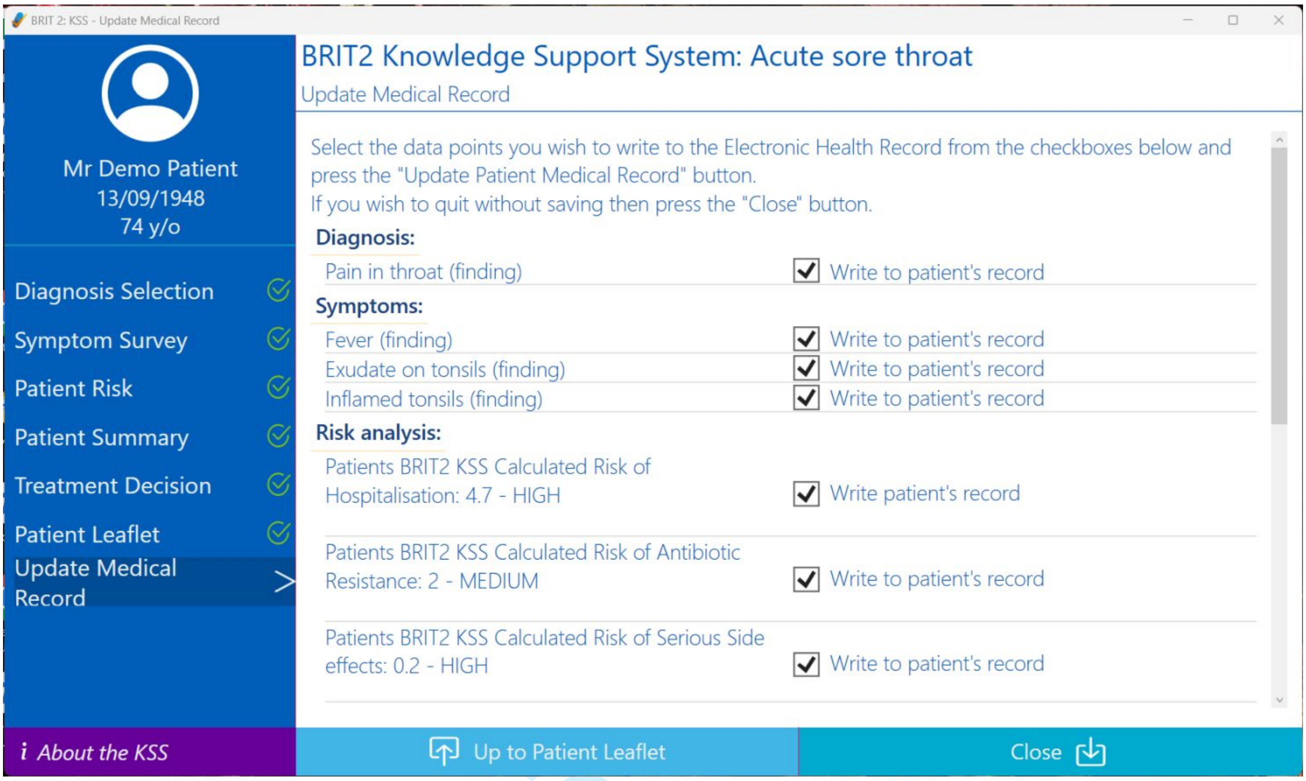
BRIT 2: KSS - Update Medical Record
BRIT2 Knowledge Support System: Acute sore throat
Update Medical Record
Mr Demo Patient
13/09/1948
74 y/o
Diagnosis Selection
Symptom Survey
Patient Risk
Patient Summary
Treatment Decision
Patient Leaflet
Update Medical Record
Select the data points you wish to write to the Electronic Health Record from the checkboxes below and press the "Update Patient Medical Record" button.
If you wish to quit without saving then press the "Close" button.
Diagnosis:
Pain in throat (finding)
Write to patient's record
Symptoms:
Fever (finding)
Write to patient's record
Exudate on tonsils (finding)
Write to patient's record
Inflamed tonsils (finding)
Write to patient's record
Risk analysis:
Patients BRIT2 KSS Calculated Risk of Hospitalisation: 4.7 - HIGH
Write patient's record
Patients BRIT2 KSS Calculated Risk of Antibiotic Resistance: 2 - MEDIUM
Write to patient's record
Patients BRIT2 KSS Calculated Risk of Serious Side effects: 0.2 - HIGH
Write to patient's record
i About the KSS
Up to Patient Leaflet
Close

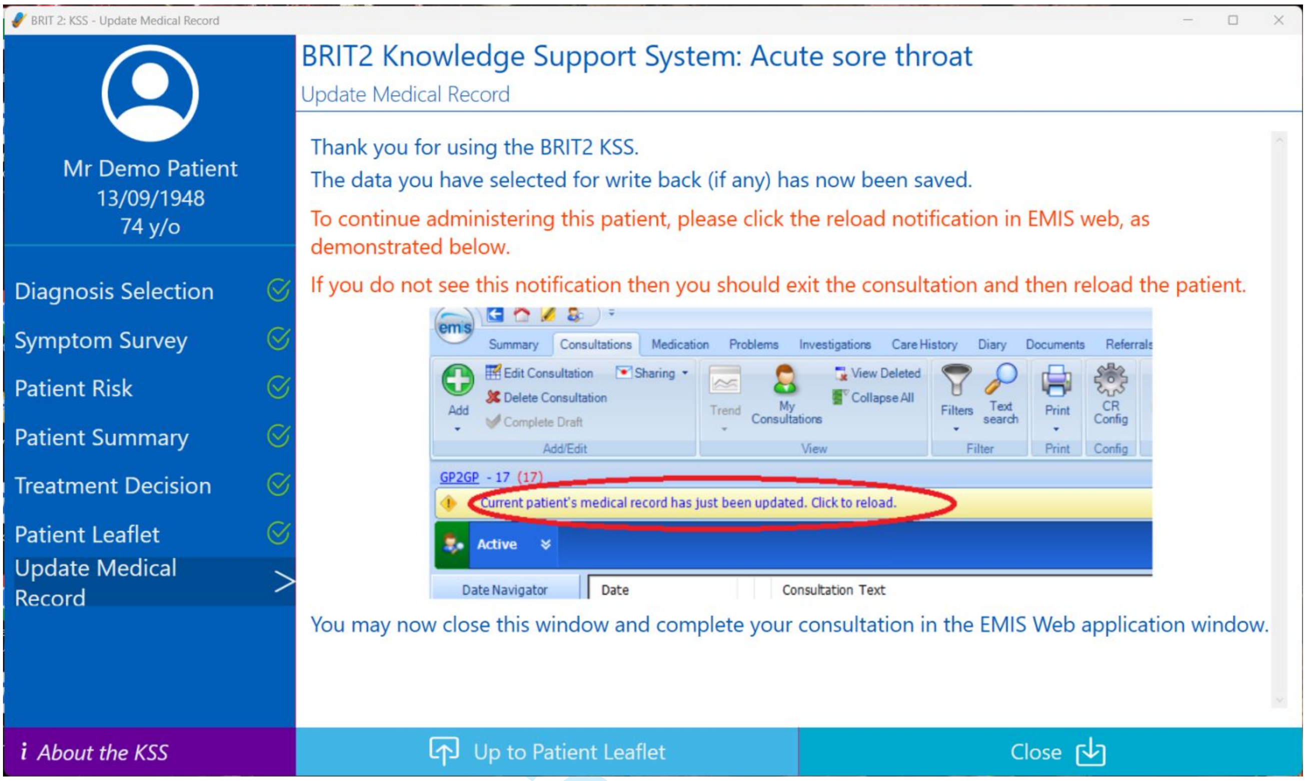

For peer review only

Click here to print the patient information leaflet

Demo Patient
Consultation: 14/12/2022 **with:** Dr Tempest
Suspected Diagnosis: Pain in throat

Explaining your treatment

Treatment: You should take pain relief and drink plenty of fluids. Ask your pharmacy for pain relief options. Use the back-up antibiotic if your symptoms do not improve within 3-5 days (currently 3 days)

Explanation: Most Pain in throat are self-limiting and get better on their own in 7 days. Return in 1 week if not better

Your risks have been compared to data from thousands of other patients like you to calculate the following:

Risk of severe complications

Your risk of severe complications due to this infection is MODERATE : Out of 100 people who had similar health to you we would expect fewer than 29 to have serious complications

Your personal risk factors identified by this analysis are: Age, High BMI, Other medical conditions

Very low Increased

Risk of antibiotic failure

Your risk of antibiotics failing this time is LOW. Around 10 per 100 patients like you will return to their doctor, for further treatment within a month.

Very low Increased

Risk of carrying antibiotic resistant bacteria

You have used 1 courses of antibiotics in the last year. There is a LOW risk that you may carry antibiotic resistant bacteria.

Very low Increased

Why was this treatment decision made?

Your health-care provider considers that an antibiotic may not be right for you at this moment. Your care provider has prescribed a back-up antibiotic to be used if you do not feel any better in 7 days. Return in 1 week if not better

Your care provider has considered the benefits of antibiotics as well your risk factors and the severity of this condition to come to this decision with you.

Type of risk	What does this risk mean to me
**Complications from your infection (hospitalisation)**	**Explanation:** Most common infections are self-limiting and will clear up on their own without medication, but some people may have a higher risk of getting 'complications' (a worse infection). This 'complications' risk score helps your doctor identify people who might have a higher risk
**Risk of resistant bacterial infection**	**Explanation:** Good bacteria helps to protect the body from infection, but antibiotics can kill all bacteria good and bad. Without good bacteria to help fight infection, antibiotic resistant bacteria can grow and take over. When a bacteria becomes "resistant" to an antibiotic, that antibiotic can no longer be used to fight that infection. The more antibiotics you take, the higher the risk of developing resistant bacteria.
**Risk of antibiotic failing**	**Explanation:** Antibiotics are effective drugs and long as they are used correctly. They do not work on viral illnesses and could cause you to have more resistant bacteria and side effects. Research has shown that some people return to the GP within a month because the antibiotic treatment has not worked.
**Side effects and severe drug reaction**	**Explanation:** Antibiotics are not a 'risk free' treatment and they can cause a lot of unpleasant side effect including: sickness/ diarrhoea, rash, thrush, stomach pains, and being sick if you drink alcohol. They can also cause reactions when used with other medication you might be taking

Don't take antibiotics unless your doctor says you need them

What if my symptoms get worse?

If you develop any of the warning signs below, call 999 (or visit your nearest A&E)

- Difficulty swallowing or breathing
- Drooling – this can be a sign of not being able to swallow
- Making a high-pitched sound as you breathe (called stridor)
- Have severe symptoms and are getting worse quickly

This Patient information leaflet has been generated by the BRIT2 project at the University of Manchester. If you have any questions, speak with your GP.

CONSORT 2010 checklist of information to include when reporting a cluster randomised trial

Section/Topic	Item No	Standard Checklist item	Extension for cluster designs	Section and Paragraph Number in Manuscript
**Title and abstract**				
	1a	Identification as a randomised trial in the title	Identification as a cluster randomised trial in the title	Title
	1b	Structured summary of trial design, methods, results, and conclusions (for specific guidance see CONSORT for abstracts)[1,2]	See table 2	Abstract
**Introduction**				
**Background and objectives**	2a	Scientific background and explanation of rationale	Rationale for using a cluster design	Introduction, Paragraphs 1 and 2
	2b	Specific objectives or hypotheses	Whether objectives pertain to the cluster level, the individual participant level or both	Introduction, Paragraph 3
**Methods**				
**Trial design**	3a	Description of trial design (such as parallel, factorial) including allocation ratio	Definition of cluster and description of how the design features apply to the clusters	Page 7
	3b	Important changes to methods after trial commencement (such as eligibility criteria), with reasons		N/A
**Participants**	4a	Eligibility criteria for participants	Eligibility criteria for clusters	Page 6
	4b	Settings and locations where the data were collected		Page 5
**Interventions**	5	The interventions for each group with	Whether interventions pertain to the cluster level, the	Pages 6-7

		sufficient details to allow replication, including how and when they were actually administered	individual participant level or both	
**Outcomes**	6a	Completely defined pre-specified primary and secondary outcome measures, including how and when they were assessed	Whether outcome measures pertain to the cluster level, the individual participant level or both	Pages 7-8
	6b	Any changes to trial outcomes after the trial commenced, with reasons		NA
**Sample size**	7a	How sample size was determined	Method of calculation, number of clusters(s) (and whether equal or unequal cluster sizes are assumed), cluster size, a coefficient of intracluster correlation (ICC or *k*), and an indication of its uncertainty	Page 7
	7b	When applicable, explanation of any interim analyses and stopping guidelines		N/A
**Randomisation:**				
**Sequence generation**	8a	Method used to generate the random allocation sequence		Page 9
	8b	Type of randomisation; details of any restriction (such as blocking and block size)	Details of stratification or matching if used	Page 7
**Allocation concealment mechanism**	9	Mechanism used to implement the random allocation sequence (such as sequentially numbered containers), describing any steps taken to conceal the sequence until interventions were assigned	Specification that allocation was based on clusters rather than individuals and whether allocation concealment (if any) was at the cluster level, the individual participant level or both	Page 7

For peer review only

**Implementation**	10	Who generated the random allocation sequence, who enrolled participants, and who assigned participants to interventions	Replace by 10a, 10b and 10c	Page 9
	10a		Who generated the random allocation sequence, who enrolled clusters, and who assigned clusters to interventions	Page 9
	10b		Mechanism by which individual participants were included in clusters for the purposes of the trial (such as complete enumeration, random sampling)	Page 7
	10c		From whom consent was sought (representatives of the cluster, or individual cluster members, or both), and whether consent was sought before or after randomisation	Page 11
**Blinding**	11a	If done, who was blinded after assignment to interventions (for example, participants, care providers, those assessing outcomes) and how		Page 9
	11b	If relevant, description of the similarity of interventions		**N/A**
**Statistical methods**	12a	Statistical methods used to compare groups for primary and secondary outcomes	How clustering was taken into account	Page 7
	12b	Methods for additional analyses, such as subgroup analyses and adjusted analyses		N/A

Results				
**Participant flow (a diagram is strongly recommended)**	13a	For each group, the numbers of participants who were randomly assigned, received intended treatment, and were analysed for the primary outcome	For each group, the numbers of clusters that were randomly assigned, received intended treatment, and were analysed for the primary outcome	N/A
	13b	For each group, losses and exclusions after randomisation, together with reasons	For each group, losses and exclusions for both clusters and individual cluster members	N/A
**Recruitment**	14a	Dates defining the periods of recruitment and follow-up		N/A
	14b	Why the trial ended or was stopped		N/A
**Baseline data**	15	A table showing baseline demographic and clinical characteristics for each group	Baseline characteristics for the individual and cluster levels as applicable for each group	N/A
**Numbers analysed**	16	For each group, number of participants (denominator) included in each analysis and whether the analysis was by original assigned groups	For each group, number of clusters included in each analysis	N/A
**Outcomes and estimation**	17a	For each primary and secondary outcome, results for each group, and the estimated effect size and its precision (such as 95% confidence interval)	Results at the individual or cluster level as applicable and a coefficient of intracluster correlation (ICC or k) for each primary outcome	Page 7
	17b	For binary outcomes, presentation of both absolute and relative effect sizes is recommended		N/A-
**Ancillary analyses**	18	Results of any other analyses performed, including subgroup analyses and adjusted		N/A-

For peer review only

		analyses, distinguishing pre-specified from exploratory		
**Harms**	19	All important harms or unintended effects in each group (for specific guidance see CONSORT for harms[3])		N/A-
**Discussion**				
**Limitations**	20	Trial limitations, addressing sources of potential bias, imprecision, and, if relevant, multiplicity of analyses		N/A
**Generalisability**	21	Generalisability (external validity, applicability) of the trial findings	Generalisability to clusters and/or individual participants (as relevant)	N/A-
**Interpretation**	22	Interpretation consistent with results, balancing benefits and harms, and considering other relevant evidence		N/A-
**Other information**				
**Registration**	23	Registration number and name of trial registry		Page 2
**Protocol**	24	Where the full trial protocol can be accessed, if available		N/A
**Funding**	25	Sources of funding and other support (such as supply of drugs), role of funders		Page 12

For peer review only

BMJ Open

Knowledge support for optimising antibiotic prescribing for common infections in general practices: evaluation of the effectiveness of periodic feedback, decision support during consultations and peer comparisons in a cluster randomised trial (BRIT2): study protocol

Journal:	*BMJ Open*
Manuscript ID	bmjopen-2023-076296.R1
Article Type:	Protocol
Date Submitted by the Author:	10-Jul-2023

Complete List of Authors:	van Staa, Tjeerd; The University of Manchester, Centre for Health Informatics<br>Sharma, Anita; NHS Oldham Clinical Commissioning Group, GP Oldham<br>Palin, Victoria ; The University of Manchester, Faculty of Biology, Medicine and Health<br>Fahmi, Ali; The University of Manchester, Centre for Health Informatics<br>Cant, Harriet; The University of Manchester, Centre for Health Informatics<br>Zhong, Xiaomin; The University of Manchester, Centre for Health Informatics<br>Jury, Francine; The University of Manchester, Centre for Health Informatics<br>Gold, Natalie; University of Oxford, Faculty of Philosophy<br>Welfare, William; UK Health Security Agency, Regions Directorate<br>Ashcroft, Darren; University of Manchester<br>Tsang, Jung Yin; University of Manchester, Centre for Primary Care and Health Services Research<br>Elliott, Rachel; The University of Manchester, Manchester Centre for Health Economics<br>Sutton, Christopher; University of Manchester, Division of Population Health, Health Services Research & Primary Care<br>Armitage, Chris; University of Manchester, Manchester Centre for Health Psychology<br>Couch, Philip; The University of Manchester, Centre for Health Informatics<br>Moulton, Georgina; The University of Manchester, Centre for Health Informatics<br>Tempest, Edward; The University of Manchester, Centre for Health Informatics<br>Buchan, Iain; University of Liverpool, Public Health and Policy

**Primary Subject Heading**:	General practice / Family practice
Secondary Subject Heading:	Infectious diseases
Keywords:	Primary Care < Primary Health Care, Electronic Health Records,

	INFECTIOUS DISEASES, Randomized Controlled Trial

SCHOLARONE™ Manuscripts

Knowledge support for optimising antibiotic prescribing for common infections in general practices: evaluation of the effectiveness of periodic feedback, decision support during consultations and peer comparisons in a cluster randomised trial (BRIT2): study protocol

Tjeerd Pieter van Staa[1], Anita Sharma[2], Victoria Palin[1,3], Ali Fahmi[1], Harriet Cant[1], Xiaomin Zhong[1], Francine Jury[1], Natalie Gold[4], William Welfare[5], Darren M Ashcroft[6,7], Jung Yin Tsang[1,6,7], Rachel A. Elliott[8], Chris Sutton[9], Christopher J. Armitage[7,10,11], Philip Couch[1], Georgina Moulton[1], Edward Tempest[1], Iain Buchan[12]

[1]Centre for Health Informatics & Health Data Research UK North, Division of Informatics, Imaging and Data Science, School of Health Sciences, Faculty of Biology, Medicine and Health, University of Manchester, Manchester Academic Health Science Centre, Manchester, UK

[2]Chadderton South Health Centre, Eaves Lane, Chadderton, Oldham, OL9 8RG, UK

[3]Maternal and Foetal Research Centre, Division of Developmental Biology and Medicine, University of Manchester, St Marys Hospital, Oxford Road, Manchester, M13 9WL, UK

[4]Faculty of Philosophy, University of Oxford, Radcliffe Humanities, Woodstock Road, Oxford OX2 6GG

[5]Regions Directorate, UK Health Security Agency, 3 Piccadilly Place, London Road, Manchester, M1 3BN, UK

[6]NIHR School for Primary Care Research, Centre for Primary Care and Health Services Research, University of Manchester, UK

[7]NIHR Greater Manchester Patient Safety Research Collaboration (GMPSRC), University of Manchester, UK

[8]Manchester Centre for Health Economics, School of Health Sciences, Faculty of Biology, Medicine and Health, The University of Manchester, Manchester, UK

[9]Centre for Biostatistics, School of Health Sciences, Faculty of Biology, Medicine and Health, University of Manchester, Manchester Academic Health Science Centre, Manchester, UK

[10]Manchester Centre for Health Psychology, University of Manchester, Oxford Road, Manchester M13 9PL

[11]Manchester University NHS Foundation Trust, Manchester Academic Health Science Centre, Manchester, M13 9PL, UK

[12]Department of Public Health, Policy and Systems, Institute of Population Health, University of Liverpool, Liverpool, UK

BRIT2 Protocol 27_11_2022 Version 2.0 IRAS 290050

Research sponsor: University of Manchester (Faculty research governance team)

Corresponding author: Tjeerd van Staa, tjeerd.vanstaa@manchester.ac.uk; ORCID ID 0000-0001-9363-742X

For peer review only

Vaughan House, Centre for Health Informatics, Division of Informatics, Imaging and Data Science, School of Health Sciences, Faculty of Biology, Medicine and Health, The University of Manchester, Manchester Academic Health Science Centre, M13 9PL Manchester, United Kingdom

Key words: antibiotics, primary care, cluster randomised trial, electronic health records, patient information

Word count 3551 words

For peer review only

For peer review only - http://bmjopen.bmj.com/site/about/guidelines.xhtml

ABSTRACT

Introduction:

This project applies a Learning Healthcare System (LHS) approach to antibiotic prescribing for common infections in primary care. The approach involves iterations of data analysis, feedback to clinicians, and implementation of quality improvement activities by the clinicians. The main research question is, can a knowledge support system (KSS) intervention within a LHS implementation improve antibiotic prescribing without increasing the risk of complications?

Methods and analysis:

A pragmatic cluster randomised controlled trial will be conducted, with randomisation of at least 112 general practices in North-West England. General practices participating in the trial will be randomised to the following interventions: periodic practice-level and individual prescriber feedback using dashboards; or the same dashboards plus a KSS. Data from large databases of healthcare records are used to characterise heterogeneity in antibiotic uses, and to calculate risk scores for clinical outcomes and for the effectiveness of different treatment strategies. The results provide the baseline content for the dashboards and KSS. The KSS comprises a display within the electronic health record used during the consultation; the prescriber (GP or allied health professional) will answer standard questions about the patient's presentation and will then be presented with information (e.g. patient's risk of complications from the infection) to guide decision making. The KSS can generate information sheets for patients, conveyed by the clinicians during consultations. The primary outcome is the practice-level rate of antibiotic prescribing (per 1000 patients) with secondary safety outcomes. The data from practices participating in the trial and the dashboard infrastructure will be held within regional shared care record systems of the National Health Service in the UK.

Ethics and dissemination: Approved by NHS Ethics Committee IRAS 290050. The research results will be published in peer-reviewed journals and also disseminated to participating clinical staff and policy and guideline developers.

Registration details: ISRCTN16230629

For peer review only - http://bmjopen.bmj.com/site/about/guidelines.xhtml

Strengths and limitations of this study

- This protocol describes a pragmatic cluster randomised controlled trial with randomisation of general practices to periodic practice-level and individual prescriber feedback using dashboards only compared to dashboards plus a knowledge support system (KSS) that can be activated during consultations.
- These interventions will be applied to antibiotic prescribing for common infections in primary care, an important area for clinical improvement given rising antimicrobial resistance.
- The design of the KSS was informed by two mixed-methods co-design workshops in which clinicians identified: key information to extract from care records (such as antibiotic prescribing history); recommended actions; personalised treatments; risk indicators; and content for patient information sheets.
- The primary research question is what is the effect on antibiotic prescribing of the KSS intervention within a learning health system implementation?
- A pilot phase will be initially conducted, with the recruitment target of 20 practices across two regions to examine feasibility and acceptability.

For peer review only

INTRODUCTION

Translating research findings into routine clinical practice is a major challenge. The Learning Healthcare System (LHS) approach has been proposed to better integrate research into clinical practice.[1] It involves iterations of data analysis (data to knowledge), feedback to clinicians (knowledge to performance) and implementation of quality improvement activities by clinicians (performance to data).[2,3] In the LHS, feedback can either be provided periodically through for example on-line dashboards or during consultations with patients using a Knowledge Support System (KSS). We use the term KSS as distinct from a Clinical Decision Support System because the information provided is about the condition, common complications and treatment options in general rather than specific decisions. Activation of KSS linked to electronic health records (EHRs) has been proposed for augmenting clinicians' knowledge during consultations with patients.[4] An icon on the clinicians' computer screen provides access to information on 'patients like mine' (e.g., the risk of developing clinical complications in similar patients), recommendations (e.g., best not prescribe amoxicillin given this patient's frequent prior use) and a patient information sheet tailored to their condition and treatment. The information on similar patients could be drawn from several sources such as historic data on clinical outcomes from comparable patient groups.[4] Thus, the KSS provides contextual information where a Clinical Decision Support System would point to a desired decision.[5]

In this project, the LHS approach will be applied to antibiotic prescribing for common infections in primary care. Primary care accounts for 72% of antibiotic prescribing in England.[6] Overuse of antibiotics is a major public health concern as it increases antimicrobial resistance. The NHS 2019 Long Term plan pledged action to optimise antibiotic uses, reducing inappropriate prescribing. The LHS approach will also include personalised patient information sheets that the clinician can provide to patients during consultation after KSS activation. A recent Cochrane review found that people exposed to decision aids feel more knowledgeable, better informed, and clearer about their values. It also found improved knowledge and accurate risk perceptions when decision aids are used within the consultation.[7]

Research questions

The research question is whether a KSS intervention within a LHS implementation can improve antibiotic prescribing without increasing the risk of complications? Practices that have implemented the KSS will be compared to practices that have not (i.e., randomised cluster trial). The LHS approach will involve detailed data analysis followed by feedback to clinicians.

METHODS AND ANALYSIS

Prior development of study interventions

Periodic feedback

For peer review only - http://bmjopen.bmj.com/site/about/guidelines.xhtml

A previous project piloted and implemented the IT infrastructure for the Learning Healthcare System (i.e., data analytics, feedback to clinicians) on antibiotic prescribing care in UK primary care. This project (BRIT: Building Rapid Interventions to reduce AMR and over-prescribing of antibiotics) was part of the £20 million Department of Health & Social Care (DHSC) funded Connected Health Cities programme (https://www.connectedhealthcities.org/). This initial BRIT project developed practice-level feedback on antibiotic together with feedback on individual prescribers.[8] These dashboards comprise a variety of antibiotic prescribing measures that were developed with clinical stakeholders.[9] They currently provide information at practice level on the drivers of antibiotic prescribing (overall and by indication), analyses of prescribing of inappropriate types of antibiotics (deviating from guideline) and extent of risk-based prescribing. Importantly, the dashboards will allow clinicians to review data at different levels, including for example prescribing rates of a specific antibiotic to the individual patients prescribed that antibiotic, and comparison to other practices in the region.[9]

KSS during consultation

Our KSS intervention was developed based on the results of two mixed-methods co-design workshops to gauge the acceptability of a prototype.[10] Clinicians identified the following key requirements: ease and efficiency of use, integration of systems, patient-centeredness, personalisation, and training. The KSS needs to include extraction of pertinent information from the care record (such as antibiotic prescribing history), recommended actions, personalised treatment, risk indicators and electronic patient information leaflets. The anticipated acceptability and intention to use the KSS, was moderate to high. While time was identified as a cost/burden, this was outweighed if the KSS improved patient outcomes and increased prescribing confidence. All the main requested features were implemented in the KSS except personalised treatment recommendations because current treatment guidelines do not cover all of the frequently encountered clinical challenges, such as frequent repeat antibiotic prescribing shortly after the first one.

Study design, participating sites and overview of interventions

The study will be a pragmatic cluster randomised controlled trial with 1:1 randomisation of practices to:

(i) Periodic feedback to general practices using practice-level + individual prescriber feedback dashboards. Appendix 1 provides examples of different dashboards.

or

(ii) Practice-level + individual prescriber feedback dashboards + KSS during consultation with the clinician providing content (such as an information sheet) that is personalised to the patient.

The study sites will include general practices located in the North-West of England that provide patient-level data to a regional shared care record system (https://www.graphnethealth.com), which is deployed in Greater Manchester, Wirral and Cheshire / Merseyside regions, together covering a 5.4 million general population.

For peer review only - http://bmjopen.bmj.com/site/about/guidelines.xhtml

The shared care record collates data from various NHS organisations to support direct patient care, service planning and research. Researchers access anonymised patient-level data through a secure data analytic environment linked to the shared care records.

Details of study interventions

Content of KSS for clinicians

Figure 1 shows the architecture of the KSS including data extracted from the patient's EHR, ability for clinicians to enter details on infection severity, estimation and presentation of risk scores, generation of personalised patient leaflet and write-back of codes to the EHR. The KSS will be available for all clinicians in the practices randomised into the KSS arm of the study. Eight screens are accessible within the KSS. Figure 2 shows Screen 1 of the KSS after activation (and Appendix 2 shows additional examples of KSS screenshots). This provides for the selection of the type of common infection and indicators of problem significance and duration. Screen 2 is the symptom survey which provides a survey with list of symptoms to capture infection severity. An example is the Feverpain score for acute sore throat, including questions such as fever in past 24 hours, presence of cough or coryza and physical exam findings.[11] Screen 3 (patient summary) includes an extraction of relevant information as recorded in the patient's EHR: the data on recent antibiotic prescribing (dates and types) and information on presence of allergies. This screen is populated automatically after KSS activation. Screen 4 (patient risks) gives personalised information including risks of:

1) Developing infection-related complications that lead to hospital admission,
2) Resistance based on number of prior antibiotics prescribed to the patient in the previous 12 months,
3) Antibiotic failure indicating probability may be prescribed another antibiotic within 30 days,
4) Severe adverse outcomes such as hospital admission for renal failure.

These personalised risk scores are based on risk prediction models including characteristics such as age, gender, clinical and medication risk factors, ethnicity and socioeconomic status. These risk prediction models were developed and tested in the OpenSAFELY platform (https://opensafely.org) or the Clinical Practice Research Datalink GOLD and Aurum (https://cprd.com/).[12,13] Risks are calculated using these algorithms which are combined with the patient's specific characteristics as recorded in the EHR. The clinician is given links to the relevant NICE guideline for the infection in Screen 5 (treatment guidelines). The clinician can then consider these risk scores alongside other information when considering whether to prescribe an antibiotic, line up a back-up antibiotic, or discuss with the patient alternative care pathways without antibiotics (treatment decision is recorded in Screen 6). Once the shared decision-making process is complete, the clinician may provide an information sheet for the patient, particularly where an antibiotic is not being prescribed to advise the patient of the decision and provide the personalised risk scores (Screen 7 with patient leaflet). Screen 8 allows the clinician to initiate a write-back function which will write back to the care record the diagnosis, symptoms and treatment as SNOMED codes. The KSS has been developed for use with EMIS

For peer review only - http://bmjopen.bmj.com/site/about/guidelines.xhtml

which is the commonest primary care record system in England.[14] Patient records remain confidential, and all data flows happen within the NHS.

KSS content for patients

We developed personalised patient information sheets that show tailored risk/benefit information based on patient-specific estimates of for example the risk of being admitted to hospital for infection-related complications. These information sheets are populated by the results of the KSS activation. The clinician can provide these to patients after consultation, in addition to any existing generic patient information sheets (TARGET) which also include information on illness duration, self-care advice, prevention advice and advice on when to re-consult [https://www.rcgp.org.uk/clinical-and-research/resources/toolkits/amr/target-antibiotics-toolkit/leaflets-to-share-with-patients.aspx]. Our advisory group including members of the public were involved in discussing and reviewing draft information sheets.

Sample size

The KSS arm will be limited to a maximum of 62 practices as this arm involves time and effort of clinicians during consultation. Sample size calculation has been computed based on the primary outcome of practice rate of antibiotic prescribing (per 1000 patients). In CPRD, the mean practice list size was 7981 and the overall rate of the number of antibiotic prescriptions (for any indication) was 598 per 1000 patients per year (standard deviation 155). Assuming a reduction of 10% in the low-intensity KSS arm, its antibiotic prescribing rate will be around 538 per 1000 patients per year. Assuming an unchanged SD of 155 and practice attrition rate of 5%, randomising 62 practices to each of the two arms will provide 90% power to detect a 10% (54 per 1000 patients per year) between-arm difference in the overall rate of antibiotic prescribing (two-sided $\alpha=0.05$), assuming a correlation of 0.82 between baseline and outcome antibiotic prescribing rate and analysing using ANCOVA with baseline prescribing rate as covariate and 5% drop-out rate. For an 80% power, 47 practices are needed in each arm. Sample size calculations were performed in PASS 2019 software. Our primary target is 112 practices although we will recruit up to 124 practices, given the limitation on numbers in the KSS arm. However, determination of the total sample size of practices will depend on practice consent to take part in the KSS evaluation and passing an assessment of IT system capability for the KSS installation.

Study outcomes

The unit of analysis will be general practice. The primary clinical outcome will be the overall rate of antibiotic prescribing. One secondary outcome, relating to safety, will be infection-related complications as recorded in the primary care record, including pneumonia and lower respiratory tract infections, peritonsillar abscess, mastoiditis, intracranial abscess, empyema, scarlet fever, pyelonephritis, septic arthritis, osteomyelitis, meningitis, toxic shock syndrome and septicaemia, and Lemierre syndrome (as defined by Gulliford et al[15]). BRIT found that clinician-recorded infection-related complications provided comparable results as those

For peer review only - http://bmjopen.bmj.com/site/about/guidelines.xhtml

recorded in linked hospital admission data.[16] Another safety outcome will be hospital admissions for infection-related complications (linked hospital data are likely to be available within the shared care-record systems). The primary and safety-related secondary outcomes are equivalent to those used in a recent trial in UK primary care of a decision support system plus patient information sheets.[17] Another secondary outcome will be the level of risk-based prescribing (i.e., the proportion of antibiotic prescribing in patients with different risks) as based on the risk prediction scores as developed and validated in the BRIT project.[16]

A Statistical Analysis Plan (SAP), to be approved by the Steering Committee, will detail the planned analyses. Data analysts will have access to anonymised data from participating practices within a secure data analytic environment. Rates will be analysed using appropriate models with Poisson or normal error structures, weighted by practice size, and with adjustment for practice-level covariates including region (Greater Manchester, Wirral, and Merseyside/Cheshire), study quarter, period of randomisation and baseline antibiotic prescribing, socioeconomic status, case-mix of patients and ethnicity distribution in each practice (including Charlson comorbidity score[18]), consultation rates for common infections, coding propensity and characteristics of patients with common infections. The coding propensity will be the proportion of antibiotic prescriptions with recent clinical record for common infection. The characteristics of patients with common infections will include the averages in each practice for mean age, sex, and predictors for infection-related complication including clinical and medication risk factors[16]). Body mass index and smoking history will also be considered as potential predictors for infection-related complications; these will be discussed with the Study Steering Committee and will each be included in either the primary analysis, a sensitivity analysis or not included in the analysis. The primary analysis will be a complete case analysis (i.e., excluding practices who drop out of the trial), unless attrition is higher than expected (i.e., >10% overall or differential between-arm attrition of >10%).

Cost-effectiveness

NICE has produced guidance on the type of economic analysis needed for digital health technologies, depending on the level of financial risk to the NHS.[19,20] For a digital health technology like KSS that has the potential to be cost-saving, the economic analysis level could be defined as 'low financial commitment' requiring at least a cost-consequence analysis (CCA) and a budget impact analysis (BIA). We will estimate the economic impact of the KSS, from the perspective of the NHS and partner social care services The primary analysis will be a within-trial CCA and BIA where the outcomes are overall rate of antibiotic prescribing and level of risk-based prescribing. As we do not have access to infection-related hospital admissions, we need to use a proxy for this aspect of resource use. Therefore, we will also carry out an indicative BRIT2 algorithm-based economic analysis where we will use the data summarised above plus estimates of the expected rate of infection-related hospital admissions based on the validated BRIT2 risk algorithms, to provide indicative estimates of overall costs associated with KSS.

For peer review only - http://bmjopen.bmj.com/site/about/guidelines.xhtml

For peer review only

In the primary CCA, we will use the primary outcome, overall level of antibiotic prescribing, level of risk-based prescribing, infection-related hospital admissions and infection-related complications, to estimate economic impact of KSS.

Pilot phase

A pilot phase will be conducted, with the initial recruitment of 20 practices in two regions. In case of the trial not meeting this target, the study is to be terminated writing up feasibility and lessons learned without major statistical or pharmacoeconomic analyses. If the pilot target is met but the study recruits less than 94 practices, statistical or pharmacoeconomic analyses will be limited due to power.

Allocation and blinding

Allocation to interventions will use anonymised practice identifiers with randomisation stratified by region (Greater Manchester, Wirral and Merseyside/Cheshire) and baseline rate of antibiotic prescribing. The project manager will recruit practices and randomly allocate using https://www.sealedenvelope.com. The data analysts will not be blinded to intervention allocation. However, the SAP for the end-of-study analyses will be developed by the Lead Statistician from the Centre for Biostatistics who will not have access to study data and practice names throughout the study, and he will also perform the checking of statistical results to ensure that they are performed in accordance with the approved SAP.

Other study-related activities

Online community of practice and professional training

An online community of practice (OCoP), also known as a virtual community of practice, will also be implemented for interested practice prescribing advisors and clinicians to share knowledge through education on the challenges within their clinics and the opportunities to improve prescribing. The OCoP will provide a critical resource to professionals who want and need recommendations, pointers, tips and tricks, best practices, insights and innovations for optimising prescribing.[21,22] The purpose of this OCoP is to facilitate dialogue among experts and clinical stakeholders, present analysis results in order to collectively discuss the local challenges and opportunities for improving antibiotic prescribing that is relevant to the user.

The OCoP will focus on discussion of complex cases (e.g., patients who frequently but intermittently are prescribed antibiotics). It will also discuss the analytics of the local data and any actions that need to be implemented and evaluated locally – this will help researchers to understand the interpretation of the dashboard and provide feedback on the dashboard. The target users will be health professionals in the participating general practices as well as local healthcare (NHS) and public health organizations. The staff will include GPs, GP Research Leads, Practice Managers, Quality and Safety Pharmacists, Medicines

For peer review only - http://bmjopen.bmj.com/site/about/guidelines.xhtml

Optimizations Pharmacists and Public Health Consultants. We have already spoken to some of the community to develop this and when developing the OCoP we will build on this to provide a platform that will suit the way this community works. We need some champions to encourage widespread adoption of the resource. We will include some workshops using video conferencing to gain some of the input for the OCoP as well as provide training.

ETHICS AND DISSEMINATION

Ethics approval

Research Ethics Committee approval has been obtained (IRAS 290050). The study will be conducted in full conformance with all relevant legal requirements and the principles of the Declaration of Helsinki, Good Clinical Practice (GCP) and the UK Policy Framework for Health and Social Care Research 2017.

Publication policy

Dissemination of research outputs to participating clinical staff and policy and guideline developers will be an integral part of this project. In addition, we will promote dissemination to national policy makers, managers and clinical leaders, through project summaries and policy briefings. All study results will be reported in accordance to this study protocol and to the Consolidated Standards of Reporting Trials statement extended to cluster randomised trials.[23] The study sponsor and funder do not have any role in data collection, management, analysis, and interpretation of data; writing of the report; and the decision to submit the report for publication.

Patient and public involvement

Patients and/or the public were involved in the design, or conduct, or reporting, or dissemination plans of this research. Participatory workshops, where stakeholders are brought together to seek their opinions, extract knowledge and solve problems, were used to inform the design of the protocol. In addition, members of the public interested in health data research were invited through advertisement to the NIHR patient research ambassador network, to form an advisory group. This group has been meeting four times throughout the course of the research with a focus on patient risk communications. Members were also invited to rotate into the trial steering committee and research team meetings to add value and report back to the public advisory group.

Steering Committee

We have established an oversight committee, the Study Steering Committee, including senior representatives independent of the trial, comprising stakeholders in general practice, pharmacy, commissioning groups and patient representatives (6-monthly meetings) and statistician. Professor Janusz Jankowski, clinician and healthcare policy expert, chairs this committee. We follow NIHR guidance for programme steering committees [Research Governance Guidelines (nihr.ac.uk)].

For peer review only - http://bmjopen.bmj.com/site/about/guidelines.xhtml

Consent

General practices will need to agree with study participation (information sheet is provided in Appendix 3) and clinicians will need to activate the KSS within the care record system. Patient consent for use of the KSS will not be sought and is not required as approved by the Ethics Committee. The reason for this is that the KSS intervention is focused on clinicians – the KSS will provide clinicians with further information. It will be up the clinician to decide whether to access or not the KSS, prescribe an antibiotic or not. The KSS makes information easily accessible to the clinicians and will not provide treatment recommendations. Studies very similar to our proposal also did not seek informed consent from patients, but, like our study, required consent by participating clinicians.[24,25]

Device classification of the KSS

The UK regulator MHRA produced guidance on how to comply with the legal requirements for interventions including software. Based on a reasonable interpretation of the MHRA guidance, BRIT2 is not considered a device for the following reasons: the KSS will not apply 'automated reasoning' to the clinician's decision to prescribe an antibiotic or not. There will be no 'if then reasoning', no direct inferences can be drawn from the BRIT2 information provided. There will be no provision of a treatment threshold; it will be up to the clinician to decide whether a BRIT2 risk estimate of infection-related complications of e.g., 1% is relevant to the patient and whether this would require an antibiotic. BRIT2 will provide 'reference information to enable a clinician to make a clinician decision'.

Technical description of the Knowledge Support System

The initial version of the KSS has been written to target the EMIS Health's EMIS Web EHR system as EMIS Health provide EHR services to the majority of general practices in the trial region. This was a strategic decision aimed at controlling the complexity of the software and managing the delivery schedule while maximising the potential targets for the KSS randomisation. Future iterations of the KSS will be platform agnostic and present data in the presentation layer of the application in a consistent way regardless of EHR software running on site and early design decisions support this future requirement.

DCMB compliance

As the KSS is to be used to support the real-time direct care of patients, a rigorous clinical risk assessment was conducted to ensure compliance with the DCB0129 standards. As developed by NHS Digital, this standard "*provides a set of requirements suitably structured to promote and ensure the effective application of clinical risk management by those organisations that are responsible for the development and maintenance of Health IT Systems for use within the health and care environment*" [https://digital.nhs.uk/data-and-information/information-standards/information-standards-and-data-collections-including-

For peer review only - http://bmjopen.bmj.com/site/about/guidelines.xhtml

extractions/publications-and-notifications/standards-and-collections/dcb0129-clinical-risk-management-its-application-in-the-manufacture-of-health-it-systems]. On evidence of compliance with the DCB0129 standards, approval was granted by EMIS to establish an API connection between KSS and the live patient record.

Acknowledgements The authors thank all the reviewers, clinicians and BRIT2 Patient Engagement Group for their assistance and support.

Contributors TPvS, ET, IB devised the project, and the main conceptual ideas. VP, NG, WW, CA, NG, WW, DA, RE, CS, CJA, GM and PC were involved in the study design. TPvS, FJ, ET, CS were involved in drafting the manuscript. AS and JT provided general practice advice and AS and FJ are involved in consenting general practice. AF, HC, and XZ are responsible for analysing data. All authors reviewed the manuscript including the revisions, approved the final manuscript and accept accountability for the accuracy and integrity of the work.

Funding This work is supported by the National Institute for Health Research (HS&DR Project: NIHR130581, NIHR Manchester Biomedical Research Centre and NIHR Greater Manchester Patient Safety Research Collaboration. The views expressed in this publication are those of the authors and not necessarily those of NIHR or the Department of Health and Social Care.

The ethics application (IRAS 290050) and trial registry (ISRCTN16230629) concern two funded projects including the NIHR funded cluster trial (as described in this study protocol) and an observational study funded by Health Data Research UK (HDR UK Better Care Programme for frail elderly) with data analysis & feedback for frail elderly. These two projects were combined in the same ethics research approval and study registration as they were to share the same data and dashboard infrastructure; interventions and study populations were to be different and both studies were separately described in the ethics application. Due to delays in getting data access, the frailty project did not progress to implementing the (dashboard) interventions. Sample size calculations were conducted separately for each project (including in the ethics application) although study registration reported overall target number of 300 for practice recruitment.

Competing interests None declared.

Patient consent for publication Not required.

Provenance and peer review Not commissioned; externally peer reviewed.

Supplemental material This content has been supplied by the author(s). It has not been vetted by BMJ Publishing Group Limited (BMJ) and may not have been peer-reviewed. Any opinions or recommendations discussed are solely those of the author(s) and are not endorsed by BMJ. BMJ disclaims all liability and responsibility arising from any reliance placed on the content. Where the content includes any translated material, BMJ does not warrant the accuracy and reliability of the translations (including but not limited to local regulations, clinical guidelines, terminology, drug names and drug dosages), and is not responsible for any error and/or omissions arising from translation and adaptation or otherwise.

For peer review only - http://bmjopen.bmj.com/site/about/guidelines.xhtml

Open access This is an open access article distributed in accordance with the Creative Commons Attribution Non Commercial (CC BY-NC 4.0) license, which permits others to distribute, remix, adapt, build upon this work non-commercially, and license their derivative works on different terms, provided the original work is properly cited, appropriate credit is given, any changes made indicated, and the use is non-commercial. See: http://creativecommons.org/licenses/by-nc/4.0/.

REFERENCES

1 Grossmann C, Sanders J, English RA, *et al.* Large Simple Trials and Knowledge Generation in a Learning Health System: Workshop Summary. Washington: : National Academies Press 2013. http://www.iom.edu/Reports/2013/Large-Simple-Trials-and-Knowledge-Generation-in-a-Learning-Health-System.aspx

2 Friedman CP, Rubin JC, Sullivan KJ. Toward an Information Infrastructure for Global Health Improvement. *Yearb Med Inform* 2017;**26**:16–23. doi:10.15265/IY-2017-004

3 Ainsworth J, Buchan I. Combining Health Data Uses to Ignite Health System Learning. *Methods Inf Med* 2015;**54**:479–87. doi:10.3414/ME15-01-0064

4 Longhurst CA, Harrington RA, Shah NH. A 'Green Button' For Using Aggregate Patient Data At The Point Of Care. *Health Aff* 2014;**33**:1229–35. doi:10.1377/hlthaff.2014.0099

5 Curtis CE, Al Bahar F, Marriott JF. The effectiveness of computerised decision support on antibiotic use in hospitals: A systematic review. *PLoS One* 2017;**12**:e0183062. doi:10.1371/journal.pone.0183062

6 English surveillance programme for antimicrobial utilisation and resistance (ESPAUR). 2022.

7 Stacey D, Légaré F, Lewis K, *et al.* Decision aids for people facing health treatment or screening decisions. *Cochrane Database Syst Rev* Published Online First: 12 April 2017. doi:10.1002/14651858.CD001431.pub5

8 Palin V, Tempest E, Mistry C, *et al.* Developing the infrastructure to support the optimisation of antibiotic prescribing using the learning healthcare system. *BMJ Heal Care Informatics* 2020;**27**. doi:10.1136/bmjhci-2020-100147

9 Palin V, Tempest E, Mistry C, *et al.* Developing the infrastructure to support the optimisation of antibiotic prescribing using the learning healthcare system to improve healthcare services in the provision of primary care in England. *BMJ Heal care informatics* 2020;**27**. doi:10.1136/bmjhci-2020-100147

10 Hurley R, Jury F, van Staa T, *et al.* Clinician acceptability of an antibiotic prescribing knowledge support system for primary care: a mixed-method evaluation of features and contexte. *BMC Heal Serv J* 2023;**23**:367. doi:10.1186/s12913-023-09239-4

For peer review only - http://bmjopen.bmj.com/site/about/guidelines.xhtml

11 Little P, Stuart B, Hobbs FDR, *et al.* Predictors of suppurative complications for acute sore throat in primary care: prospective clinical cohort study. *BMJ* 2013;**347**:f6867. doi:10.1136/bmj.f6867

12 Zhong X, Pate A, Yang Y-T, *et al.* The impact of COVID -19 on antibiotic prescribing in primary care in England: evaluation and risk prediction of appropriateness of type and repeat prescribing. *J Infect* Published Online First: May 2023. doi:10.1016/J.JINF.2023.05.010

13 Fahmi A, Palin V, Zhong X, *et al.* Evaluation of the impact of COVID-19 pandemic on hospital admission related to common infections. *submitted*

14 Kontopantelis E, Stevens RJ, Helms PJ, *et al.* Spatial distribution of clinical computer systems in primary care in England in 2016 and implications for primary care electronic medical record databases: a cross-sectional population study. *BMJ Open* 2018;**8**:e020738. doi:10.1136/bmjopen-2017-020738

15 Gulliford MC, Juszczyk D, Prevost AT, *et al.* Electronically delivered interventions to reduce antibiotic prescribing for respiratory infections in primary care: Cluster RCT using electronic health records and cohort study. *Health Technol Assess (Rockv)* 2019;**23**:1–72. doi:10.3310/hta23110

16 Mistry C, Palin V, Li Y, *et al.* Development and validation of a multivariable prediction model for infection-related complications in patients with common infections in UK primary care and the extent of risk-based prescribing of antibiotics. *BMC Med* 2020;**18**:118. doi:10.1186/s12916-020-01581-2

17 Gulliford MC, Prevost AT, Charlton J, *et al.* Effectiveness and safety of electronically delivered prescribing feedback and decision support on antibiotic use for respiratory illness in primary care: REDUCE cluster randomised trial. *BMJ* 2019;**364**:l236. doi:10.1136/bmj.l236

18 Charlson M, Wells MT, Ullman R, *et al.* The Charlson comorbidity index can be used prospectively to identify patients who will incur high future costs. *PLoS One* 2014;**9**:e112479. doi:10.1371/journal.pone.0112479

19 Evidence standards framework for digital health technologies | Our programmes | What we do | About | NICE. https://www.nice.org.uk/about/what-we-do/our-programmes/evidence-standards-framework-for-digital-health-technologies (accessed 4 Mar2019).

20 Craig J. National Institute for Health and Care Excellenc e. Evidence Standards Framework for Digital Health Technologies Examples of Functional Classifications for Selected Digital Health Technologies. 2019.

21 Barnett S, Jones SC, Bennett S, *et al.* General practice training and virtual communities of practice - a review of the literature. *BMC Fam Pract* 2012;**13**:87. doi:10.1186/1471-2296-13-87

22 Bermejo-Caja CJ, Koatz D, Orrego C, *et al.* Acceptability and feasibility of a virtual community of practice to primary care professionals regarding patient empowerment: a qualitative pilot study. *BMC Health Serv Res* 2019;**19**:403. doi:10.1186/s12913-019-4185-z

23 Campbell MK, Piaggio G, Elbourne DR, *et al.* Consort 2010 statement: Extension to cluster randomised trials. *BMJ* 2012;**345**. doi:10.1136/bmj.e5661

24 Gulliford MC, Prevost AT, Charlton J, *et al.* Effectiveness and safety of electronically delivered prescribing feedback and decision support on antibiotic use for respiratory illness in primary care:

For peer review only - http://bmjopen.bmj.com/site/about/guidelines.xhtml

REDUCE cluster randomised trial. *BMJ* 2019;**364**:l236. doi:10.1136/bmj.l236

25 Gulliford MC, van Staa TP, McDermott L, *et al.* Cluster randomized trials utilizing primary care electronic health records: Methodological issues in design, conduct, and analysis (eCRT Study). *Trials* 2014;**15**. doi:10.1186/1745-6215-15-220

For peer review only

For peer review only - http://bmjopen.bmj.com/site/about/guidelines.xhtml

Figure 1: Architecture of the KSS

Figure 2: Example of a KSS screen after activation within the care record system during consultation highlighting the patient's details (fictional) with the active record and the various screen available to the clinician

For peer review only

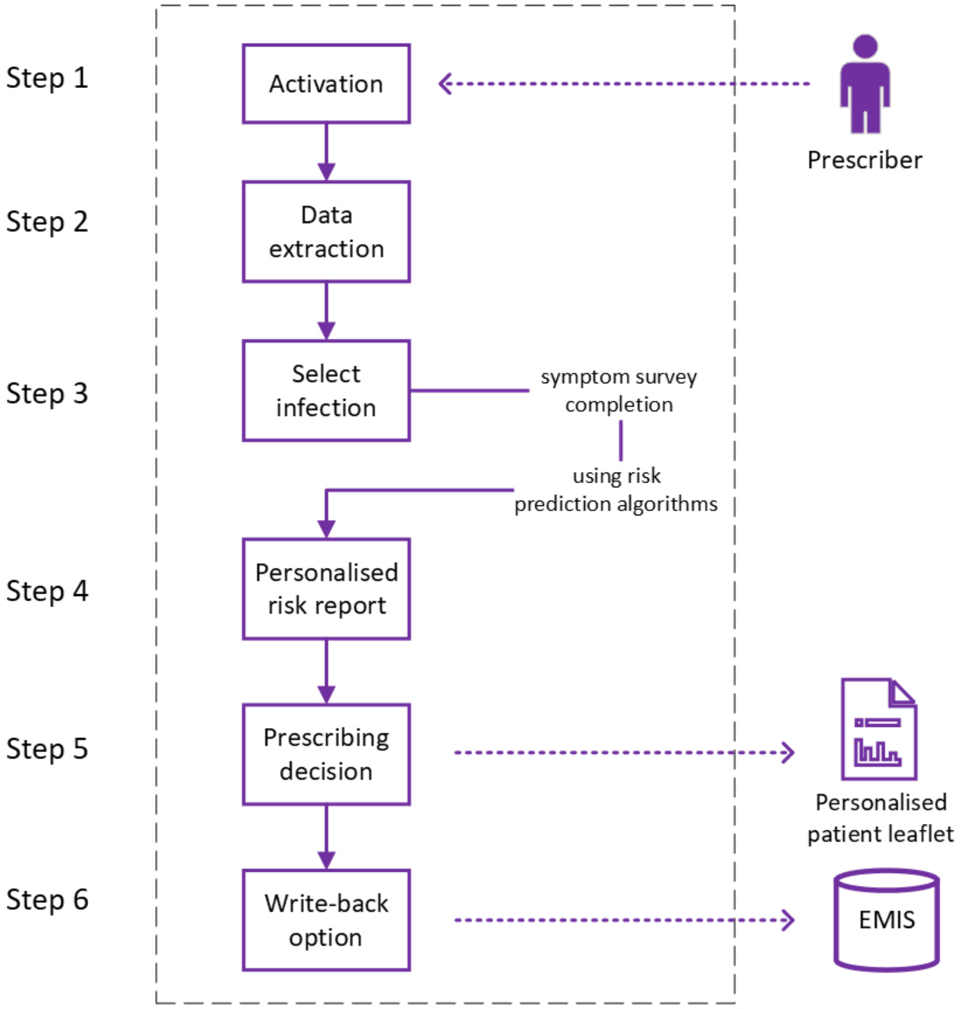

Architecture of the KSS

381x389mm (57 x 57 DPI)

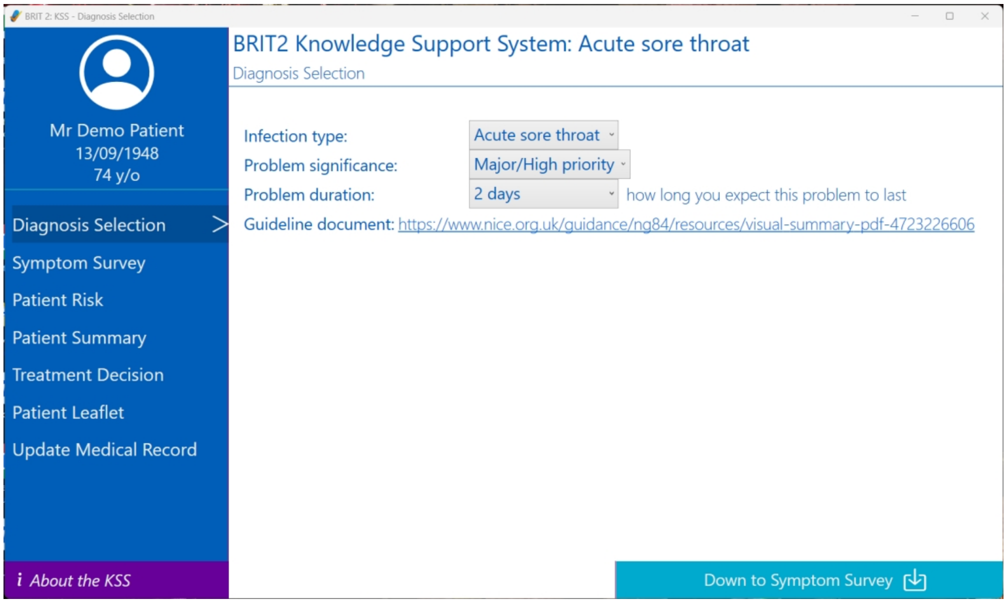

Example of a KSS screen after activation within the care record system during consultation highlighting the patient’s details (fictional) with the active record and the various screen available to the clinician

904x536mm (47 x 47 DPI)

Appendix 1: Examples of dashboards as developed in BRIT project

Benchmarking

Data from large national data sources have been and will be used to get better understanding of the drivers for heterogeneity in care and for the effectiveness of different treatment strategies. The results will provide the baseline content for the dashboards.

A previous project piloted and implemented the IT infrastructure for the Learning Healthcare System (i.e., data analytics, feedback to clinicians) on antibiotic prescribing care in UK primary care, This project (BRIT: Building Rapid Interventions to reduce AMR and over-prescribing of antibiotics) was part of the £20 million Department of Health & Social Care (DHSC) funded Connected Health Cities programme (https://www.connectedhealthcities.org/). The data analytics activities in BRIT consisted of benchmarking current practice in primary care, evaluating the levels of suboptimal antibiotic prescribing and identifying opportunities for improvement. Large variability in antibiotic prescribing was observed between practices and within practices: Change points in prescribing did not reflect updates to national guidelines. Prescribing levels within practices were not consistent for different infectious conditions. BRIT also found high levels of prescribing of potentially inappropriate type of antibiotics which were highest for otitis externa (67.3%) and upper respiratory tract infection (38.7%). BRIT found that that over the last 15 years antibiotic prescribing in primary care was not risk-based: patients with very low risk of infection-related hospital admissions were as likely to receive an antibiotic as patients with higher risks. BRIT also evaluated the effectiveness of treating common infections with antibiotics. The findings also indicate that incidental use of antibiotics is effective in reducing infection-related hospital admissions while repeated courses of antibiotics may have limited benefit and be indicative of adverse outcomes. Of 5.1 million antibiotics prescribed in UK primary care, only 14.8% were given to patients without any antibiotic prescribing in the previous three years and 43.6% are for patients who already received 5+ antibiotic prescriptions in the previous three years. These BRIT findings indicate that optimal antibiotic prescribing in primary care is a complex interplay of a patient's symptoms, age and co-morbidity and previous history of antibiotic use. While incidental use of an antibiotic may reduce the risk of infection-related complications, it may also decrease the effectiveness of the antibiotic for future infections (possibly due to the development of resistance in the patient [1]). This highlights the importance for patient-specific communication, information and algorithms that recommend best course of action.

Recent research found considerable variability between GPs in the case mix of patients consulting and large variability in antibiotic prescribing habits. The majority of clinicians (> 95%) prescribed at least one antibiotic measure that was above the medians of their peers. It concluded that there is a need for a wider range of objectives (using a variety of measures without ranking of clinicians based on a single metric), varying engagement strategies with feedback tailored to each clinician, local context including bespoke

For peer review only

recommendations that could be implemented and proactive support from colleagues and local organisations [2].

The LHS approach is not just about analysis of data and feedback of results to clinicians. An important aspect is around understanding and changing behaviours which is a focus of behavioural sciences [3]. An example of this is the provision of social norm practice-level feedback (i.e., comparing a clinician to other peers). In a UK cluster randomised controlled trial (cRCT), this approach was found to substantially reduce antibiotic prescribing. Every GP in the feedback intervention group was sent a letter from England's Chief Medical Officer [4]. A US cRCT tested the effects of several behavioural interventions and found that peer comparisons resulted in lower rates of inappropriate antibiotic prescribing for acute respiratory tract infection. These comparisons consisted of emails sent to clinicians that compared their antibiotic prescribing rates with those with the lowest inappropriate prescribing rates [5].

The BRIT dashboard summarises information on antibiotic prescribing by infection type and where these prescriptions may deviate from the recommended guidelines, overall prescribing rates with comparision to peers, as well as how a practice may prescribe based on a pratients risk of an infection-related complication. On the hompage users have the option to clickthe lage icons or use the menu bar across the top to navigete to each dashbord.

The first group of visualisations (Supplementary Figure 1A) shows the general practices (GPs) inforation about their antibiotic prescribing by infectious conditions. The first part of the dashboard shows colourful notification boxes with the number of infection-related consultations in a given time period (yellow box), the number of antibiotic prescriptions issued for these consultations (blue box), the number of these prescriptions issed that deviated from the national recommended guidelines (red box) and a nudging notification box (in green) that suggests areas for improvement in their prescribing based on which condition saw more deviating prescriptions. Users reviewing the dashboard, such as prescribing advisor may decide to concentrate their efforts when auditing the practices antibiotic prescribing in a given month by delving deeper into the prescriptions issued for consultations were the system flagged the most inappropriate prescriptions (in this example upper respiratory tract infections; URTI). Next the user will see a box and whisker plot showing national prescribing rates by infection, i.e. for each practice the percentage of consultations that resulted in an antibiotic prescription by infection type (Supplementary Figure 1B). The users of the BRIT dashboard can also see where their practice sits (the black dots overlaid on this plot) compared to the national prescribing rates. For example, Supplementary Figure 1 shows the practice has a higher prescribing rate for breast-related infections compared to the national average but have a lover prescribing rate for ear-related infections (otitis externa and otitis media), highlighting which infections warrant further investigation in their practice. The practice can also see what prescriptions are being prescribed to each infectious condition as a percentage in a sunburst plot, where Supplementary figure 1C shows that when selecting lower respiratiory tract infections

For peer review only

(LRTI) over three quarters of the prescriptions their practice prescribed for this condition were fore amoxicillian, followed by doxycycline and clarithromycin as well as other antibiotic less commonly prescribed to these conditionsn in their practice in a given time period.

The practice can also see how their practice is performing in terms of prescribing appropriateness (Figure 1D). The analysis behind these plots assess wheather each antibioitc prescription in agreement (blue) or disagreement (red) with the recommended first- second- or alternative- medicine. For example this plot shows a hypothetical practice were 24% of the consultations for sinusitis resulted in an antibiotic prescription that may have been inappropriate because it deviated form the recommended guidelines. They can also see for each condition what antibiotic type was issued and flagged as potentially inappropriate (figure not shown). If they were to scroll down on the sape dashboard there are snapshots of the most recent antibiotic guidelines , enabiling a quick comparision of the antibiotics they have prescribed deviating from recommendations and refreshing what antibiotics are currently recommended appropriate for each condition. Again, this analysis highlights areas for review/improvement specific to each practice that may instigate discussion in a practice manegement meeting ensuring prescribers have refreshed their knowledge on the current prescribing guidelines for a particular infection.

A second topic of analysis is looking at how well a practice prescribed based on a patients predicted risk of a poor outcome (e.g., infection-related hospitalisation). The analysis is based on a validated risk prediction model using two national datasets . Supplementary Figure 2 shows the predicted risk (using the prediction model) for every patient presenting with a particular infection (in this case LRTI) in a given time period (6 months), separated into categories from very low predicted risk to very high predicted risk of complication. For each category the actual prescribing rate is caluculated as a percentage and displayed by the bule bars. Practices prescribing according to patients risk of complication would expect to see the height of the blue bars match the red line here, where very low risk patients received prescriptions on occasion, perhapse due to the severity of their symptom and the very high risk patients are receiving a prescription more often. However, this figure shows a flat line across all risk categories again suggesting prescribing for this infection is not well targeted to high risk patients and there is room for improvement. The dashboard also has a built in a risk prediction calculator, allowing users modify various patient characteristics and see which effects the predicted risk more, again adding a training element to the dashboard that can help to identify ways to further optimise their prescribing.

The final topic of analysis is prescribing benchmarking. The traffic-light visulisation (Supplementary figure 3A) shows STAR-PU adjusted prescriing rates for each practice. This is an age-sex adjusted prescribing rate allowing fair comparison between practices. The yellow dots show bottom 25% of practices with a relatively low prescribing rate, the red dots show the top 25% of high prescribing practices and the green is the middle 50% percent of practices with an average prescribing rate. The black dot then shows where the individual

For peer review only

practice sits compared to their peers. The bottom figure (Supplementary figure 3B) shows the practices prescribing rate over time. This is important compare how a practice is prescribing to their peerd but to also observe any rapid changes to their prescribing rates following an intervention; allowing rapid review and uptake of interventions that work and removal of those that do not. The practice can also use these sort of comparison plots to see seasonality trends as well as changes in prescribing due to an outbreak of a pandemic disease, such as COVID-19. Here we can see the prescribing rate in April 2018 and April 2019 for this demostrative practice was 31.5 units of antibiotics for every 1000 registered patients; however following the outbreak of COVID-19 the prescribing rate in April 2020 has reduced to 23.85 units of antibiotics prescribed per 1000 registered patients.

The data in the dashboard is regularly updated providing practices with frequent incited to their own prescribing and how changes they make can alter and optimise their prescribing. The content of the dahboard visualisations also evolving in an iterative manner based on feedback from users and key stakholders, creating a learning health care system that is evolving with the health care our patner practices provide.

For peer review only

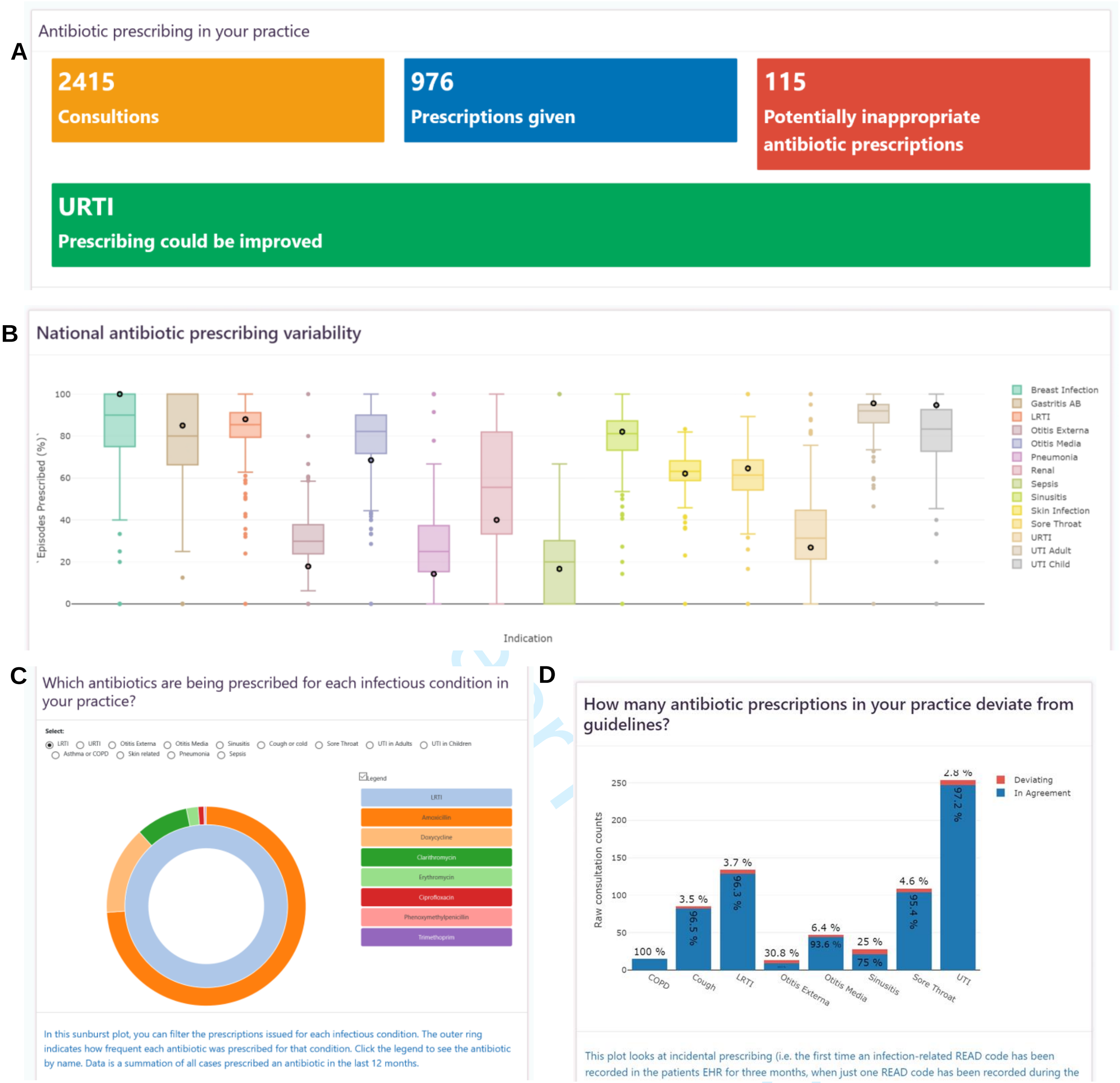

Supplementary Figure 1: Examples of dashboards providing periodic feedback on antibiotic prescribing of practices

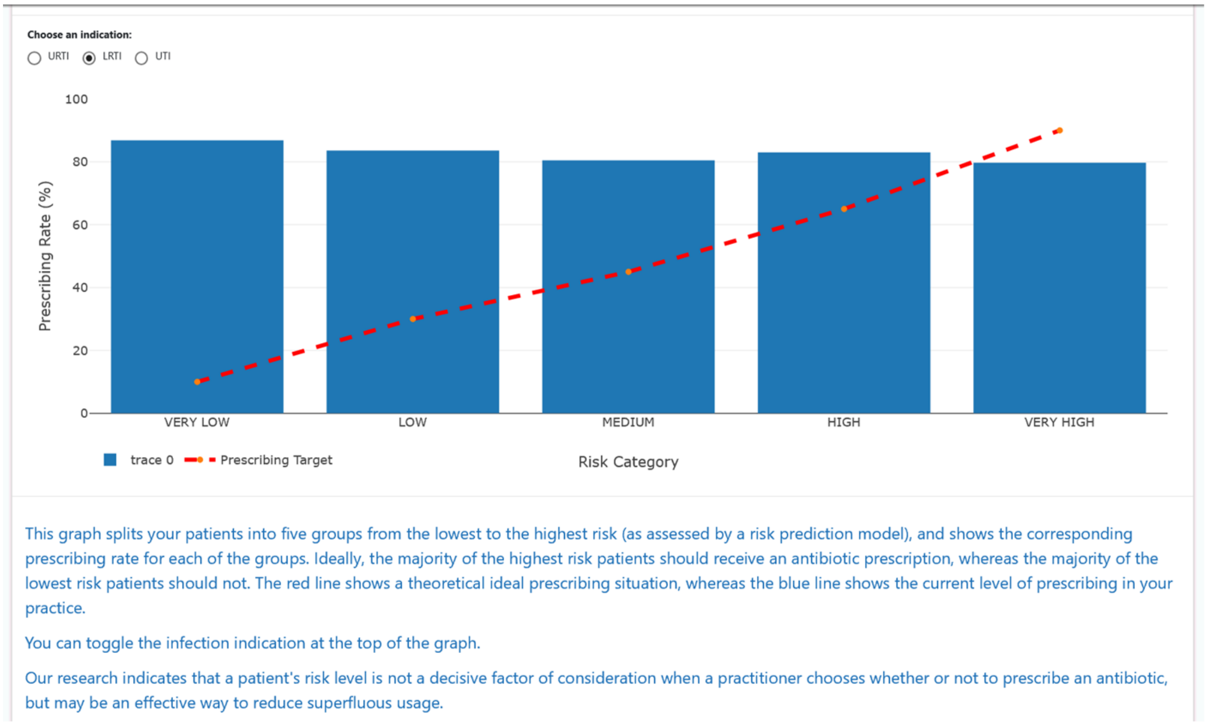

Supplementary Figure 2: dashboards of antibiotic prescribing by patient risk profile

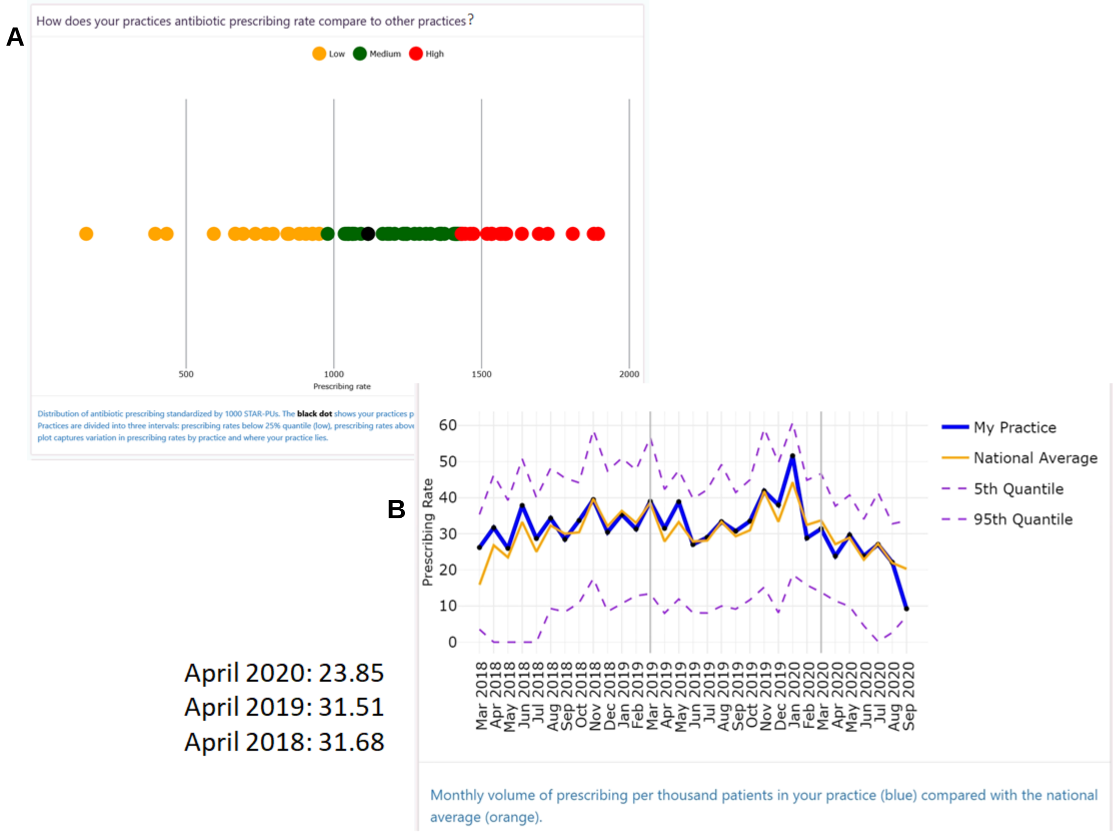

Supplementary Figure 3: Dashboards of practice-level antibiotic prescribing rates

References

1. Costelloe C, Metcalfe C, Lovering A, Mant D, Hay AD. Effect of antibiotic prescribing in primary care on antimicrobial resistance in individual patients: systematic review and meta-analysis. BMJ [Internet]. 2010 [cited 2018 Sep 15];340:c2096. Available from: http://www.ncbi.nlm.nih.gov/pubmed/20483949
2. Van Staa T, Li Y, Gold N, Chadborn T, Welfare W, Palin V, et al. Comparing antibiotic prescribing between clinicians in UK primary care: an analysis in a cohort study of eight different measures of antibiotic prescribing. BMJ Qual Saf [Internet]. BMJ Qual Saf; 2022 [cited 2022 Sep 6];bmjqs-2020-012108. Available from: https://pubmed.ncbi.nlm.nih.gov/35241573/
3. Improving people's health: Applying behavioural and social sciences to improve population health and wellbeing in England [Internet]. 2018. Available from: www.facebook.com/PublicHealthEngland
4. Hallsworth M, Chadborn T, Sallis A, Sanders M, Berry D, Greaves F, et al. Provision of social norm feedback to high prescribers of antibiotics in general practice: a pragmatic national randomised controlled trial. Lancet [Internet]. 2016 [cited 2018 Sep 15];387:1743–52. Available from: http://www.ncbi.nlm.nih.gov/pubmed/26898856
5. Meeker D, Linder JA, Fox CR, Friedberg MW, Persell SD, Goldstein NJ, et al. Effect of behavioral interventions on inappropriate antibiotic prescribing among primary care practices a randomized clinical trial. JAMA - J Am Med Assoc. American Medical Association; 2016;315:562–70.

For peer review only

Appendix 2: Examples of KSS screens and personalised patient leaflet

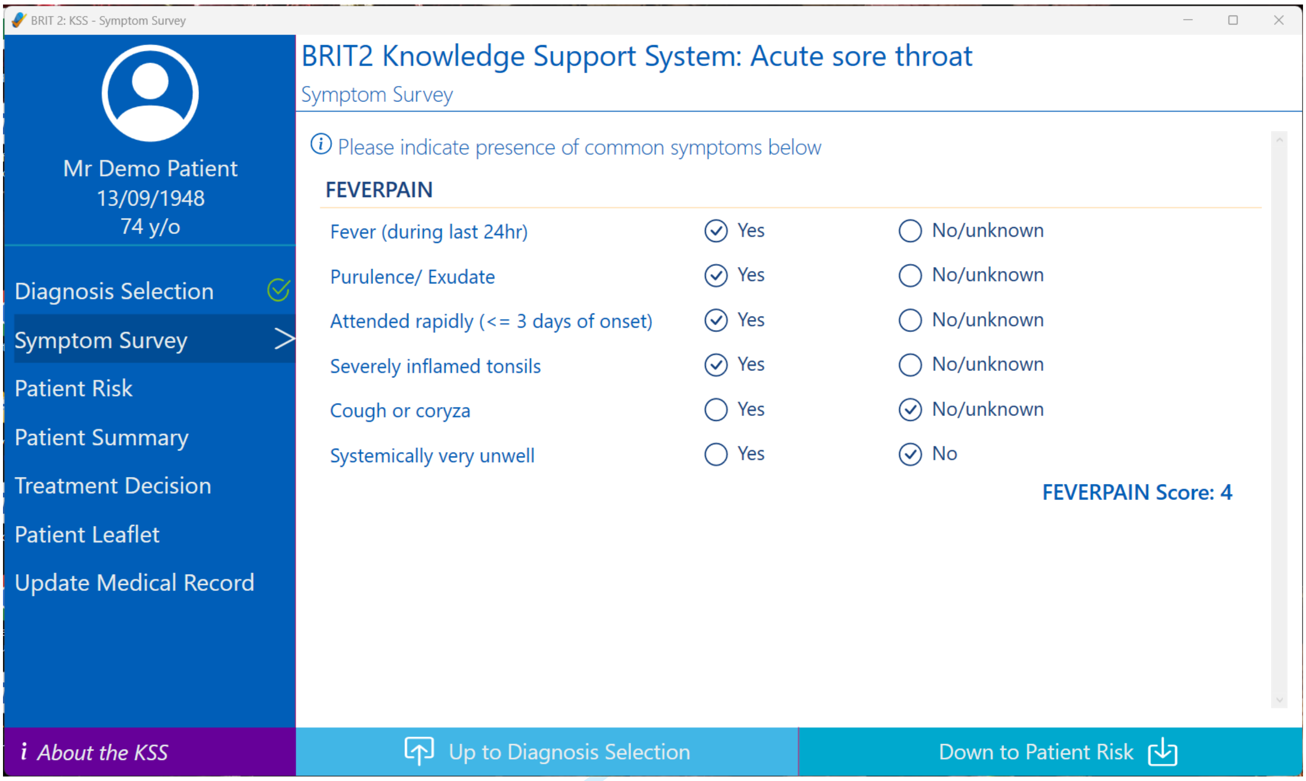

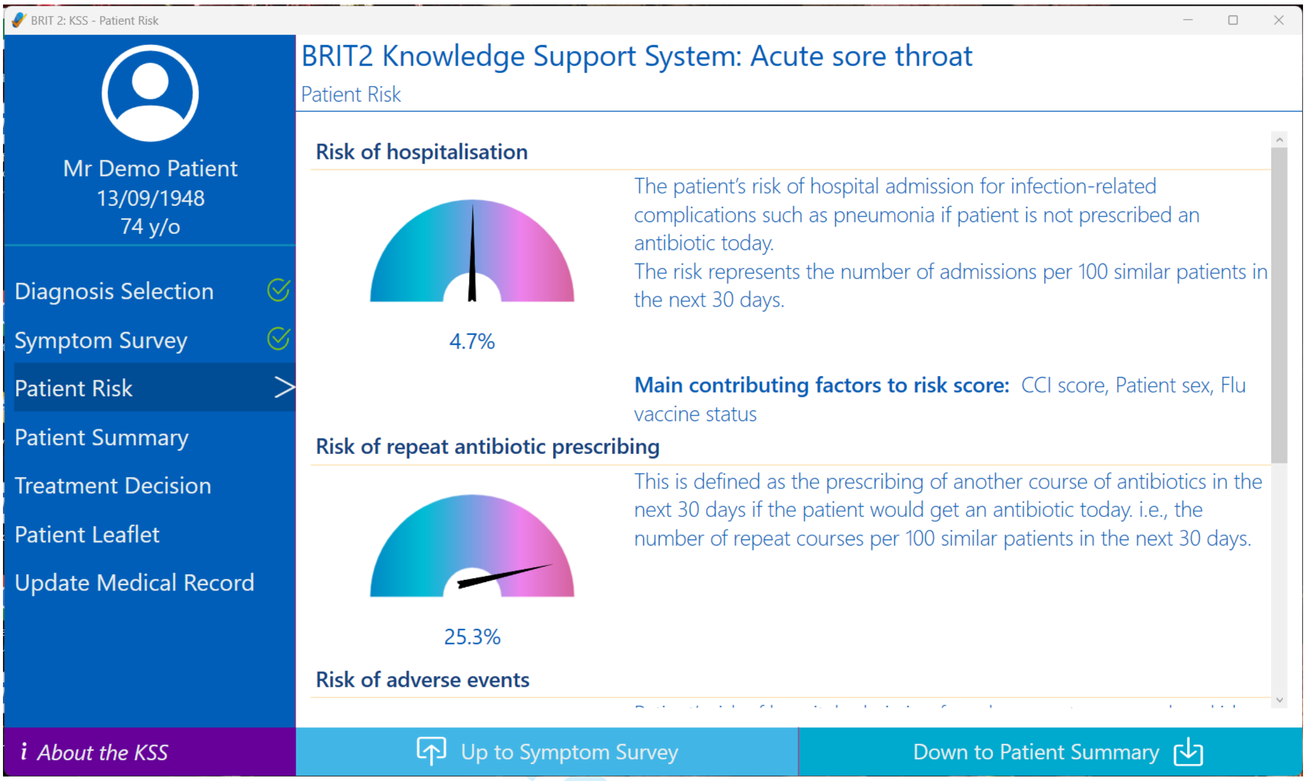

For peer review only

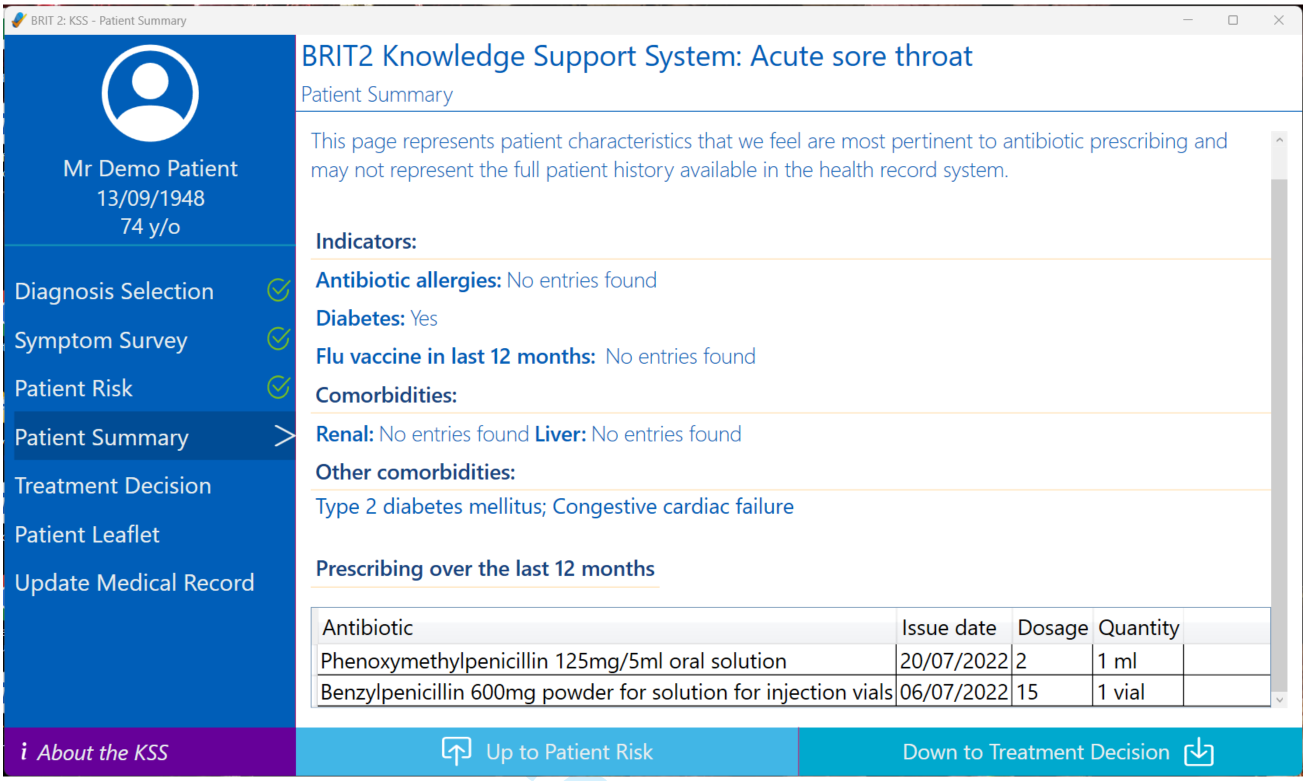

For peer review only

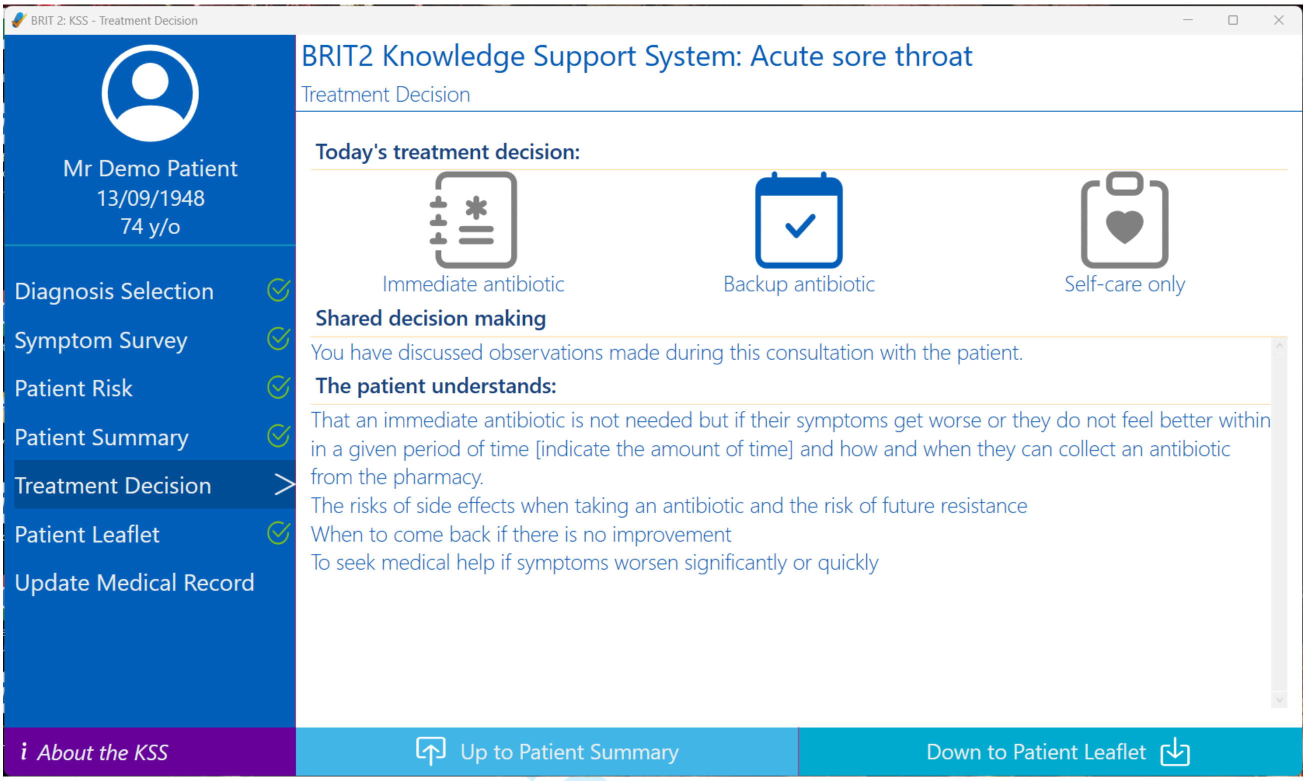
BRIT 2: KSS - Treatment Decision
Mr Demo Patient
13/09/1948
74 y/o
Diagnosis Selection
Symptom Survey
Patient Risk
Patient Summary
Treatment Decision
Patient Leaflet
Update Medical Record
About the KSS
BRIT2 Knowledge Support System: Acute sore throat
Treatment Decision
Today's treatment decision:
Immediate antibiotic
Backup antibiotic
Self-care only
Shared decision making
You have discussed observations made during this consultation with the patient.
The patient understands:
That an immediate antibiotic is not needed but if their symptoms get worse or they do not feel better within in a given period of time [indicate the amount of time] and how and when they can collect an antibiotic from the pharmacy.
The risks of side effects when taking an antibiotic and the risk of future resistance
When to come back if there is no improvement
To seek medical help if symptoms worsen significantly or quickly
Up to Patient Summary
Down to Patient Leaflet

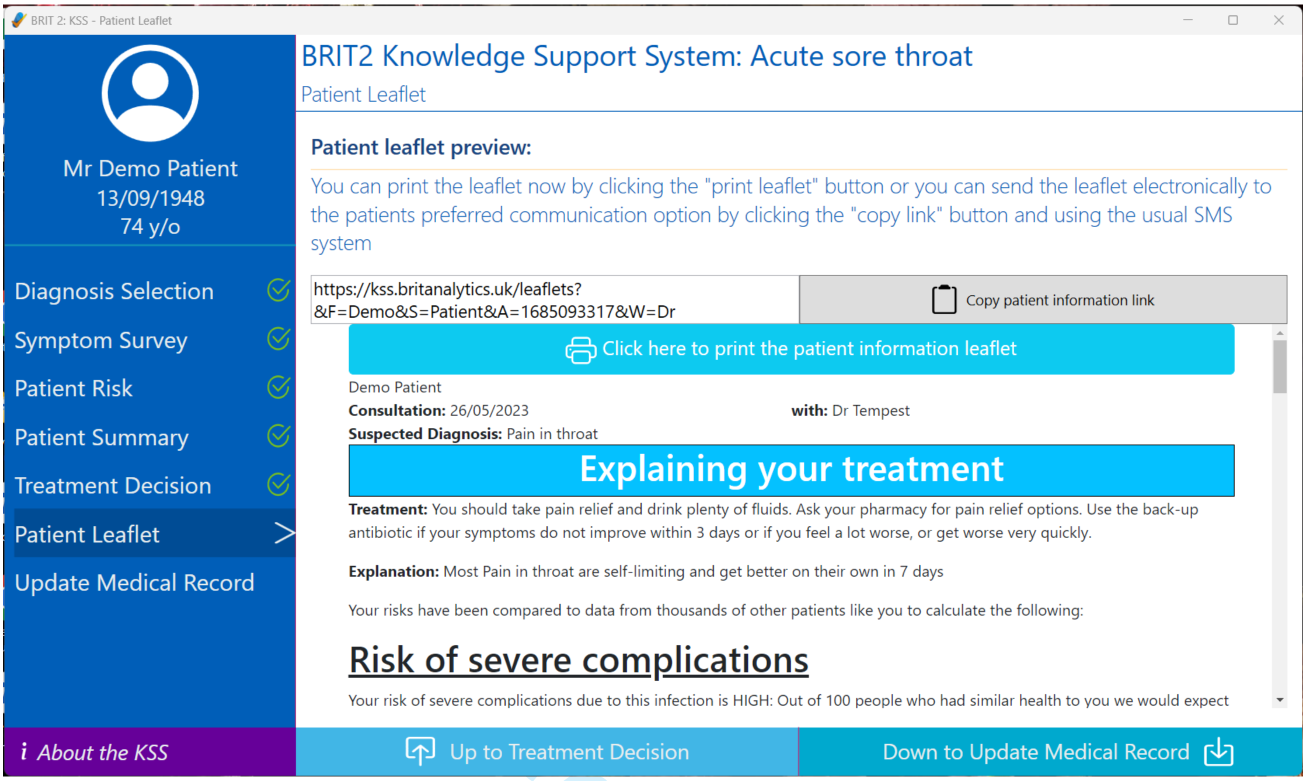
BRIT 2: KSS - Patient Leaflet
BRIT2 Knowledge Support System: Acute sore throat
Patient Leaflet
Mr Demo Patient
13/09/1948
74 y/o
Diagnosis Selection
Symptom Survey
Patient Risk
Patient Summary
Treatment Decision
Patient Leaflet
Update Medical Record
Patient leaflet preview:
You can print the leaflet now by clicking the "print leaflet" button or you can send the leaflet electronically to the patients preferred communication option by clicking the "copy link" button and using the usual SMS system
https://kss.britanalytics.uk/leaflets?&F=Demo&S=Patient&A=1685093317&W=Dr
Copy patient information link
Click here to print the patient information leaflet
Demo Patient
Consultation: 26/05/2023
with: Dr Tempest
Suspected Diagnosis: Pain in throat
Explaining your treatment
Treatment: You should take pain relief and drink plenty of fluids. Ask your pharmacy for pain relief options. Use the back-up antibiotic if your symptoms do not improve within 3 days or if you feel a lot worse, or get worse very quickly.
Explanation: Most Pain in throat are self-limiting and get better on their own in 7 days
Your risks have been compared to data from thousands of other patients like you to calculate the following:
Risk of severe complications
Your risk of severe complications due to this infection is HIGH: Out of 100 people who had similar health to you we would expect
About the KSS
Up to Treatment Decision
Down to Update Medical Record

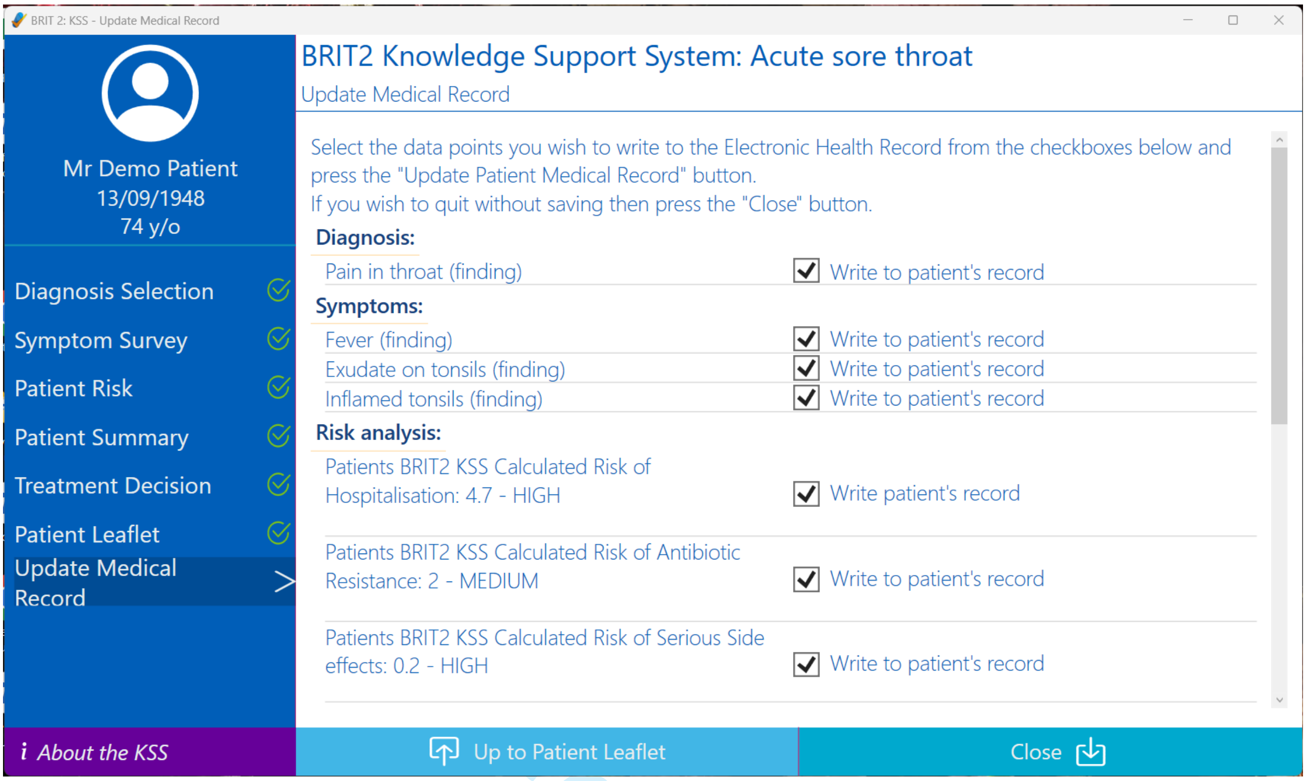

For peer review only

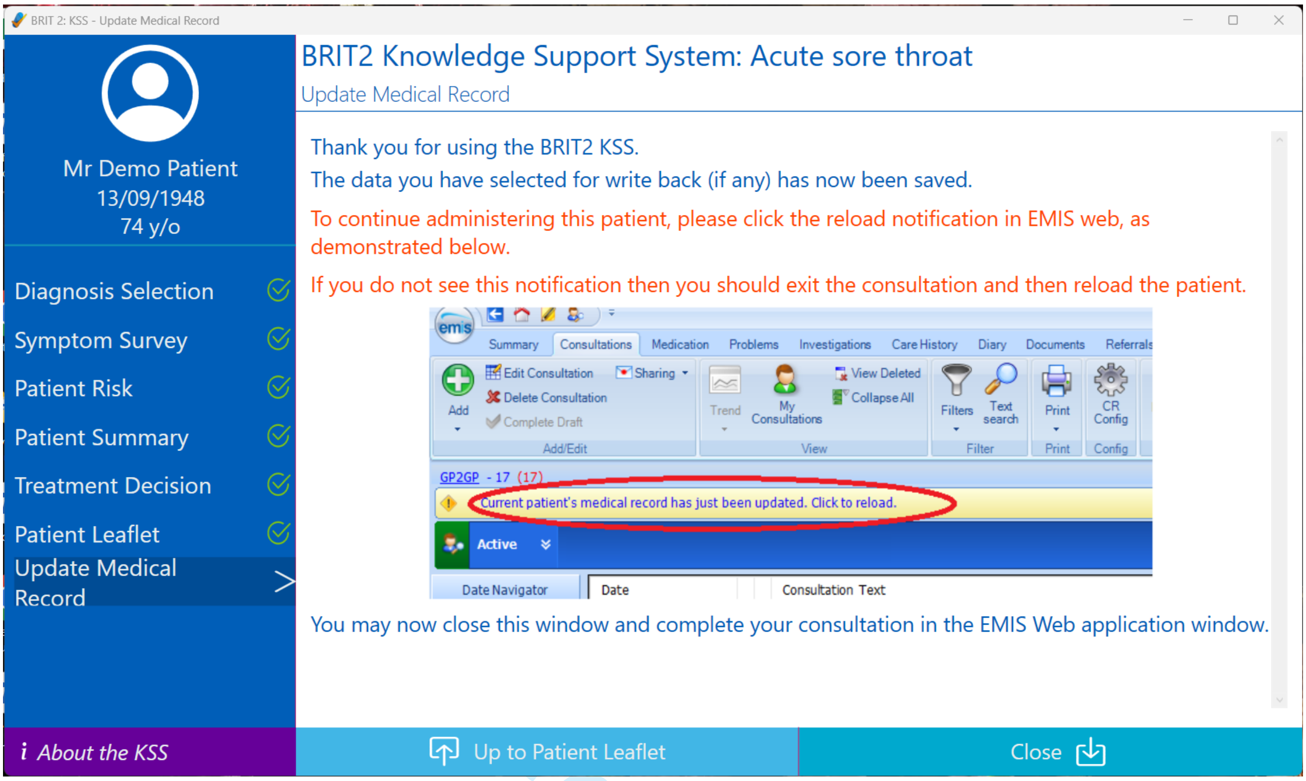

For peer review only

Click here to print the patient information leaflet

Demo Patient
Consultation: 14/12/2022 **with:** Dr Tempest
Suspected Diagnosis: Pain in throat

Explaining your treatment

Treatment: You should take pain relief and drink plenty of fluids. Ask your pharmacy for pain relief options. Use the back-up antibiotic if your symptoms do not improve within 3-5 days (currently 3 days)

Explanation: Most Pain in throat are self-limiting and get better on their own in 7 days. Return in 1 week if not better

Your risks have been compared to data from thousands of other patients like you to calculate the following:

Risk of severe complications

Your risk of severe complications due to this infection is MODERATE : Out of 100 people who had similar health to you we would expect fewer than 29 to have serious complications

Your personal risk factors identified by this analysis are: Age, High BMI, Other medical conditions

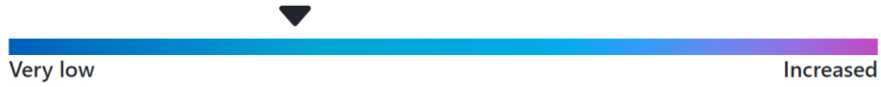

Risk of antibiotic failure

Your risk of antibiotics failing this time is LOW. Around 10 per 100 patients like you will return to their doctor, for further treatment within a month.

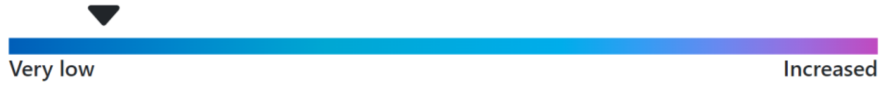

Risk of carrying antibiotic resistant bacteria

You have used 1 courses of antibiotics in the last year. There is a LOW risk that you may carry antibiotic resistant bacteria.

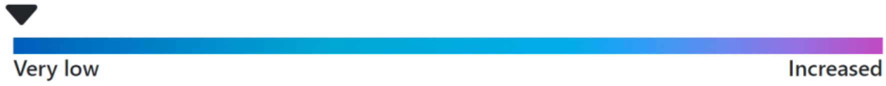

Why was this treatment decision made?

Your health-care provider considers that an antibiotic may not be right for you at this moment. Your care provider has prescribed a back-up antibiotic to be used if you do not feel any better in 7 days. Return in 1 week if not better

Your care provider has considered the benefits of antibiotics as well your risk factors and the severity of this condition to come to this decision with you.

Type of risk	What does this risk mean to me
Complications from your infection (hospitalisation)	**Explanation:** Most common infections are self-limiting and will clear up on their own without medication, but some people may have a higher risk of getting 'complications' (a worse infection). This 'complications' risk score helps your doctor identify people who might have a higher risk
Risk of resistant bacterial infection	**Explanation:** Good bacteria helps to protect the body from infection, but antibiotics can kill all bacteria good and bad. Without good bacteria to help fight infection, antibiotic resistant bacteria can grow and take over. When a bacteria becomes "resistant" to an antibiotic, that antibiotic can no longer be used to fight that infection. The more antibiotics you take, the higher the risk of developing resistant bacteria.
Risk of antibiotic failing	**Explanation:** Antibiotics are effective drugs and long as they are used correctly. They do not work on viral illnesses and could cause you to have more resistant bacteria and side effects. Research has shown that some people return to the GP within a month because the antibiotic treatment has not worked.
Side effects and severe drug reaction	**Explanation:** Antibiotics are not a 'risk free' treatment and they can cause a lot of unpleasant side effect including: sickness/ diarrhoea, rash, thrush, stomach pains, and being sick if you drink alcohol. They can also cause reactions when used with other medication you might be taking

Don't take antibiotics unless your doctor says you need them

What if my symptoms get worse?

If you develop any of the warning signs below, call 999 (or visit your nearest A&E)

- Difficulty swallowing or breathing
- Drooling – this can be a sign of not being able to swallow
- Making a high-pitched sound as you breathe (called stridor)
- Have severe symptoms and are getting worse quickly

This Patient information leaflet has been generated by the BRIT2 project at the University of Manchester. If you have any questions, speak with your GP.

MANCHESTER 1824
The University of Manchester

Building Resources to Improve Treatments (BRIT2)

A study of interventions to improve quality of care and optimisation of antibiotic prescribing using actionable information from advanced analytics.

Information Sheet: GM CARE RECORD

V1.0 12.10.2022

Your practice is being invited to take part in this study because your practice data forms part of the GM Integrated Care Record. Anonymised data from primary care is a valuable source for research, and has been a useful resource in understanding patient pathways in the COVID-19 pandemic. The attached Data Sharing Agreement (DSA) provided by Health Innovation Manchester asks practices to continue to provide anonymised data for research purposes to the BRIT2 research project at the University of Manchester.

The BRIT2 project

GP practices in the North West of England have been identified as high prescribers of antibiotics. Reducing or optimising prescribing will help to address issues of antimicrobial resistance (AMR) but better tools are needed to support GP practices to do this.

Researchers at the University of Manchester have developed a series of dashboards that uses data from GP practices to help understand where to focus efforts to optimise prescribing. The data the researchers get access to is anonymised at source, and remains in the datacente (authorised access provided to researchers). However, the GP practices get to see an identifiable version which allows a drill down to individual patients if necessary. For example, the dashboards can identify a number of patients who were inappropriately prescribed. The practice can look in more detail at who these patients were and understand where the issues might lie.

https://vimeo.com/764472108 (Test version, does not include enhanced dashboards)

The BRIT research team have also co-designed (though GP workshops) and developed a knowledge support system (KSS). The KSS supports the search and retrieval of context-specific knowledge from the Electronic Health Record system, to facilitate decision making at the point of care for common infections where an antibiotic may be considered as therapy. Characteristics of individual patients are also incorporated into risk analyses to generate patient specific risk assessments and are presented to prescribers for consideration. Appropriate NICE guidelines are provided for reference. Personalised patient leaflets can be generated to support patient understanding of prescribing decisions.

For peer review only - http://bmjopen.bmj.com/site/about/guidelines.xhtml

MANCHESTER 1824
The University of Manchester

The KSS is a software package that has been built to integrate with the electronic health record system provided by EMISHealth. EMIS are the leading GP clinical IT system provider in the North West of England where the BRIT2 study is taking place. The KSS has been fully tested and validated by EMISHealth before being used in this study.

https://vimeo.com/766587482 (example, not final version)

Taking part in the BRIT2 research project.

We are offering the practice level dashboards to any practice who would like to see them – there is no obligation to take part in the study. Practices can use the dashboards as long as the study runs.

For the research study, We are looking for practices who would support the trial of the KSS and individual prescriber dashboards in a randomised controlled trial. A nominated individual in the practice will need to be a point of contact for the research team.

Randomisation will be to one of two groups, either A+B (Practice level and individual dashboards or A+B+C (includes the KSS)

All practices and individuals will receive a reminder once a quarter whilst the study is ongoing to remind them to view the dashboards. Training videos and support from the research team will be provided in the use of the KSS and navigating the dashboards.

Researchers will be able to see when dashboards and the KSS have been activated. This is an observational function of the study to allow researchers to report back to the funder if these systems are used, and the effectiveness of the systems. It does not allow researchers to see how the user is interacting with the dashboard or the KSS, only that they have opened it.

How long will the trial last?

The full trial is expected to last for 12 months (Dashboard) with use of the KSS for 9 months from July23 to March 24 to span the flu season.

Is there any payment to take part?

There is a payment of £250 to each practice who takes part in the trial, to cover associated research costs (time to read and sign documentation etc)

There is an additional payment of up to £1611 to each practice randomised to the KSS arm to cover time taken to use the KSS in consultation with patients.

For peer review only - http://bmjopen.bmj.com/site/about/guidelines.xhtml

MANCHESTER 1824
The University of Manchester

What happens next?

If you agree to be part of this study, you will be asked to sign the attached:

1. Data Sharing agreement – this authorises use of anonymised data so that the practice can have their enhanced dashboards created.
2. Organisational Information Document: - this is a contract between the University of Manchester and the practice which allows the University to pay the research costs detailed above

Once documents have been received practices will need to request access to dashboards (the process for this will be provided)

Practices will then be randomised into the 2 arms of the study and will be informed of which arm they are in.

Those practices randomised into the KSS arm will be contacted by the IT service provider for their practice to allow installation of the KSS onto the practice systems. This is a tested and validated process that will not affect practice systems.

Anonymised data in the Trustworthy research environment will be accessed on a monthly basis by researchers as part of the research study.

Further information

This study is being organised and managed by the University of Manchester and funded by Public Health England (PHE), the National Institute of Health Research (NIHR) and Health Data Research UK (HDRUK). All patient-level data to be used for analysis will be anonymised and held in a secure Trustworthy Research Environments at Graphnet Health Ltd. The researchers will not disclose the names of practices or clinicians to people or organisations that have not been approved by the Data Controller. Any publications that are created following this study will use only anonymous information. No references to individual practices or prescribers will be made.

The study has been independently reviewed and given approval by the North East - Newcastle & North Tyneside 2 Research Ethics Committee Committee ref: 21/NE/0103 IRAS 290050

Thank you for reading the information sheet and for considering taking part in this research study.

The research behind the study: Recent research by the University of Manchester evaluated a variety of antibiotic (AB) prescribing measures for individual UK GPs, including overall,

For peer review only - http://bmjopen.bmj.com/site/about/guidelines.xhtml

repeat or incidental AB prescribing, AB types prescribed and extent of risk-based prescribing (for infection-related hospital admissions). Results showed four-fold variability in overall prescribing rate, variability in clinician's propensity to repeat antibiotic prescribing and, variability in use of broad spectrum antibiotics. Almost all (96.4%) of clinicians exceeded the prescribing threshold for at least one prescribing measure. Those at higher risk of hospitalisation from an infection related condition, particularly frail elderly, those on multiple medications for comorbidities, are subject to the most variability in care. Targeting on risk-based prescribing would have the largest impact on optimising use of antibiotics in primary care. The research concluded that there is a need for a wider range of objectives to improve prescribing and quality of care, including varying engagement strategies with feedback tailored to each clinician, local context including bespoke recommendations that could be implemented and proactive support from colleagues and local organisations. Previously Public health England (PHE) have sought to impact AB prescribing using a variety of measures, including letters from the Chief Medical Officer to GPs in the top 20% of prescribers, which led to a 3.3-3.7% reduction in prescribing. In collaboration with GP's, AHPs prescribing advisors, patients, NICE and Public Health England (UK_HSA) , the BRIT2 research team have developed a series of dashboards that aim to help GPs adapt their prescribing practice to optimise the use of antibiotics, reduce inappropriate prescribing and use data to assist in evaluation and reporting antibiotic prescribing.

Relevant publications from this research group:

van der Zande, M.M., Dembinsky, M., Aresi, G. et al. General practitioners' accounts of negotiating antibiotic prescribing decisions with patients: a qualitative study on what influences antibiotic prescribing in low, medium and high prescribing practices. BMC Fam Pract 20, 172 (2019). https://doi.org/10.1186/s12875-019-1065-x

Victoria Palin, Anna Mölter, Miguel Belmonte, Darren M Ashcroft, Andrew White, William Welfare, Tjeerd van Staa, Antibiotic prescribing for common infections in UK general practice: variability and drivers, Journal of Antimicrobial Chemotherapy, Volume 74, Issue 8, August 2019, Pages 2440–2450, https://doi.org/10.1093/jac/dkz163

Magdalena Nowakowska, Tjeerd van Staa, Anna Mölter, Darren M Ashcroft, Jung Yin Tsang, Andrew White, William Welfare, Victoria Palin, Antibiotic choice in UK general practice: rates and drivers of potentially inappropriate antibiotic prescribing, Journal of Antimicrobial Chemotherapy, Volume 74, Issue 11, November 2019, Pages 3371–3378, https://doi.org/10.1093/jac/dkz345

Li, Y., Sperrin, M., Belmonte, M. et al. Do population-level risk prediction models that use routinely collected health data reliably predict individual risks? Sci Rep 9, 11222 (2019). https://doi.org/10.1038/s41598-019-47712-5

Yan Li, Anna Mölter, Andrew White, William Welfare, Victoria Palin, Miguel Belmonte, Darren M Ashcroft, Matthew Sperrin, Tjeerd Pieter van Staa. Relationship between prescribing of antibiotics and other medicines in primary care: a cross-sectional study. British Journal of General Practice 2019; 69 (678): e42-e51. DOI: 10.3399/bjgp18X700457

For peer review only - http://bmjopen.bmj.com/site/about/guidelines.xhtml

MANCHESTER 1824
The University of Manchester

Anna Mölter, Miguel Belmonte, Victoria Palin, Chirag Mistry, Matthew Sperrin, Andrew White, William Welfare, Tjeerd Van Staa, Antibiotic prescribing patterns in general medical practices in England: Does area matter?, Health & Place, Volume 53, 2018, Pages 10-16, ISSN 1353-8292,https://doi.org/10.1016/j.healthplace.2018.07.004.

van Staa, T.P., Palin, V., Li, Y. et al. The effectiveness of frequent antibiotic use in reducing the risk of infection-related hospital admissions: results from two large population-based cohorts. BMC Med 18, 40 (2020). https://doi.org/10.1186/s12916-020-1504-5

Tjeerd Pieter van Staa, Victoria Palin, Benjamin Brown, William Welfare, Yan Li, Darren M Ashcroft, The Safety of Delayed Versus Immediate Antibiotic Prescribing for Upper Respiratory Tract Infections, Clinical Infectious Diseases, Volume 73, Issue 2, 15 July 2021, Pages e394–e401, https://doi.org/10.1093/cid/ciaa890

Victoria Palin, William Welfare, Darren M Ashcroft, Tjeerd Pieter van Staa, Shorter and Longer Courses of Antibiotics for Common Infections and the Association With Reductions of Infection-Related Complications Including Hospital Admissions, Clinical Infectious Diseases, Volume 73, Issue 10, 15 November 2021, Pages 1805–1812, https://doi.org/10.1093/cid/ciab159

Van Staa T, Li Y, Gold N, et al; Comparing antibiotic prescribing between clinicians in UK primary care: an analysis in a cohort study of eight different measures of antibiotic prescribing. BMJ Quality & Safety Published Online First: 03 March 2022. doi: 10.1136/bmjqs-2020-012108

Mistry, C., Palin, V., Li, Y. et al. Development and validation of a multivariable prediction model for infection-related complications in patients with common infections in UK primary care and the extent of risk-based prescribing of antibiotics. BMC Med 18, 118 (2020). https://doi.org/10.1186/s12916-020-01581-2

Ruth, Hurley, et al. "Clinician acceptability of an antibiotic prescribing knowledge support system for primary care: a mixed-method evaluation of features and context." (2022). https://doi.org/10.21203/rs.3.rs-1839693/v1

For peer review only - http://bmjopen.bmj.com/site/about/guidelines.xhtml

CONSORT 2010 checklist of information to include when reporting a cluster randomised trial

Section/Topic	Item No	Standard Checklist item	Extension for cluster designs	Section and Paragraph Number in Manuscript
**Title and abstract**				
	1a	Identification as a randomised trial in the title	Identification as a cluster randomised trial in the title	Title
	1b	Structured summary of trial design, methods, results, and conclusions (for specific guidance see CONSORT for abstracts)[1,2]	See table 2	Abstract
**Introduction**				
**Background and objectives**	2a	Scientific background and explanation of rationale	Rationale for using a cluster design	Introduction, Paragraphs 1 and 2
	2b	Specific objectives or hypotheses	Whether objectives pertain to the cluster level, the individual participant level or both	Introduction, Paragraph 3
**Methods**				
**Trial design**	3a	Description of trial design (such as parallel, factorial) including allocation ratio	Definition of cluster and description of how the design features apply to the clusters	Page 7
	3b	Important changes to methods after trial commencement (such as eligibility criteria), with reasons		N/A
**Participants**	4a	Eligibility criteria for participants	Eligibility criteria for clusters	Page 6
	4b	Settings and locations where the data were collected		Page 5
**Interventions**	5	The interventions for each group with	Whether interventions pertain to the cluster level, the	Pages 6-7

		sufficient details to allow replication, including how and when they were actually administered	individual participant level or both	
**Outcomes**	6a	Completely defined pre-specified primary and secondary outcome measures, including how and when they were assessed	Whether outcome measures pertain to the cluster level, the individual participant level or both	Pages 7-8
	6b	Any changes to trial outcomes after the trial commenced, with reasons		NA
**Sample size**	7a	How sample size was determined	Method of calculation, number of clusters(s) (and whether equal or unequal cluster sizes are assumed), cluster size, a coefficient of intracluster correlation (ICC or *k*), and an indication of its uncertainty	Page 7
	7b	When applicable, explanation of any interim analyses and stopping guidelines		N/A
**Randomisation:**				
**Sequence generation**	8a	Method used to generate the random allocation sequence		Page 9
	8b	Type of randomisation; details of any restriction (such as blocking and block size)	Details of stratification or matching if used	Page 7
**Allocation concealment mechanism**	9	Mechanism used to implement the random allocation sequence (such as sequentially numbered containers), describing any steps taken to conceal the sequence until interventions were assigned	Specification that allocation was based on clusters rather than individuals and whether allocation concealment (if any) was at the cluster level, the individual participant level or both	Page 7

For peer review only

**Implementation**	10	Who generated the random allocation sequence, who enrolled participants, and who assigned participants to interventions	Replace by 10a, 10b and 10c	Page 9
	10a		Who generated the random allocation sequence, who enrolled clusters, and who assigned clusters to interventions	Page 9
	10b		Mechanism by which individual participants were included in clusters for the purposes of the trial (such as complete enumeration, random sampling)	Page 7
	10c		From whom consent was sought (representatives of the cluster, or individual cluster members, or both), and whether consent was sought before or after randomisation	Page 11
**Blinding**	11a	If done, who was blinded after assignment to interventions (for example, participants, care providers, those assessing outcomes) and how		Page 9
	11b	If relevant, description of the similarity of interventions		**N/A**
**Statistical methods**	12a	Statistical methods used to compare groups for primary and secondary outcomes	How clustering was taken into account	Page 7
	12b	Methods for additional analyses, such as subgroup analyses and adjusted analyses		N/A
**Results**				
**Participant flow (a diagram is**	13a	For each group, the numbers of participants	For each group, the numbers of clusters that were randomly	N/A

**strongly recommended)**		who were randomly assigned, received intended treatment, and were analysed for the primary outcome	assigned, received intended treatment, and were analysed for the primary outcome	
	13b	For each group, losses and exclusions after randomisation, together with reasons	For each group, losses and exclusions for both clusters and individual cluster members	N/A
**Recruitment**	14a	Dates defining the periods of recruitment and follow-up		N/A
	14b	Why the trial ended or was stopped		N/A
**Baseline data**	15	A table showing baseline demographic and clinical characteristics for each group	Baseline characteristics for the individual and cluster levels as applicable for each group	N/A
**Numbers analysed**	16	For each group, number of participants (denominator) included in each analysis and whether the analysis was by original assigned groups	For each group, number of clusters included in each analysis	N/A
**Outcomes and estimation**	17a	For each primary and secondary outcome, results for each group, and the estimated effect size and its precision (such as 95% confidence interval)	Results at the individual or cluster level as applicable and a coefficient of intracluster correlation (ICC or k) for each primary outcome	Page 7
	17b	For binary outcomes, presentation of both absolute and relative effect sizes is recommended		N/A-
**Ancillary analyses**	18	Results of any other analyses performed, including subgroup analyses and adjusted analyses, distinguishing pre-specified from exploratory		N/A-

**Harms**	19	All important harms or unintended effects in each group (for specific guidance see CONSORT for harms[3])		N/A-
**Discussion**				
**Limitations**	20	Trial limitations, addressing sources of potential bias, imprecision, and, if relevant, multiplicity of analyses		N/A
**Generalisability**	21	Generalisability (external validity, applicability) of the trial findings	Generalisability to clusters and/or individual participants (as relevant)	N/A-
**Interpretation**	22	Interpretation consistent with results, balancing benefits and harms, and considering other relevant evidence		N/A-
**Other information**				
**Registration**	23	Registration number and name of trial registry		Page 2
**Protocol**	24	Where the full trial protocol can be accessed, if available		N/A
**Funding**	25	Sources of funding and other support (such as supply of drugs), role of funders		Page 12

For peer review only
