# Supplementary material for: Knowledge support for optimising antibiotic prescribing for common infections in general practices: evaluation of the effectiveness of periodic feedback, decision support during consultations and peer comparisons in a cluster randomised trial (BRIT2) – study protocol
Source: BMJ Open. 2023 Aug 22;13(8):e076296. doi: 10.1136/bmjopen-2023-076296 (PMC10445367; doi:10.1136/bmjopen-2023-076296)
Supplement: Reviewer comments [file bmjopen-2023-076296.reviewer_comments.pdf]

PEER REVIEW HISTORY

BMJ Open publishes all reviews undertaken for accepted manuscripts. Reviewers are asked to complete a checklist review form (**http://bmjopen.bmj.com/site/about/resources/checklist.pdf**) and are provided with free text boxes to elaborate on their assessment. These free text comments are reproduced below.

ARTICLE DETAILS

**TITLE (PROVISIONAL)**	Knowledge support for optimising antibiotic prescribing for common infections in general practices: evaluation of the effectiveness of periodic feedback, decision support during consultations and peer comparisons in a cluster randomised trial (BRIT2): study protocol
**AUTHORS**	van Staa, Tjeerd; Sharma, Anita; Palin, Victoria; Fahmi, Ali; Cant, Harriet; Zhong, Xiaomin; Jury, Francine; Gold, Natalie; Welfare, William; Ashcroft, Darren; Tsang, Jung Yin; Elliott, Rachel; Sutton, Christopher; Armitage, Chris; Couch, Philip; Moulton, Georgina; Tempest, Edward; Buchan, Iain

This article was not externally reviewed at BMJ Open. Protocols that have been independently assessed prior to submission to BMJ Open are usually fast-tracked to publication on the grounds that further substantial changes will not be possible. This independent assessment will usually be external, independent review for both a substantial grant award from a non-commercial or government funder and ethics approval.
